# Structure- and conformation-activity studies of nociceptin/orphanin FQ receptor dimeric ligands

**DOI:** 10.1038/srep45817

**Published:** 2017-04-06

**Authors:** Salvatore Pacifico, Alfonso Carotenuto, Diego Brancaccio, Ettore Novellino, Erika Marzola, Federica Ferrari, Maria Camilla Cerlesi, Claudio Trapella, Delia Preti, Severo Salvadori, Girolamo Calò, Remo Guerrini

**Affiliations:** 1 Department of Chemical and Pharmaceutical Sciences and LTTA, University of Ferrara, 44121 Ferrara, Italy; 2 Department of Pharmacy, University of Naples “Federico II”, 80131 Naples, Italy; 3 Department of Agraria (QuaSic.A.Tec.), Università Mediterranea di Reggio Calabria, 89122 – Reggio Calabria, Italy; 4 Department of Medical Sciences, Section of Pharmacology and National Institute of Neuroscience, University of Ferrara, 44121 Ferrara, Italy

## Abstract

The peptide nociceptin/orphanin FQ (N/OFQ) and the N/OFQ receptor (NOP) constitute a neuropeptidergic system that modulates various biological functions and is currently targeted for the generation of innovative drugs. In the present study dimeric NOP receptor ligands with spacers of different lengths were generated using both peptide and non-peptide pharmacophores. The novel compounds (12 peptide and 7 nonpeptide ligands) were pharmacologically investigated in a calcium mobilization assay and in the mouse vas deferens bioassay. Both structure- and conformation-activity studies were performed. Results demonstrated that dimerization did not modify the pharmacological activity of both peptide and non-peptide pharmacophores. Moreover, when dimeric compounds were obtained with low potency peptide pharmacophores, dimerization recovered ligand potency. This effect depends on the doubling of the C-terminal address sequence rather than the presence of an additional N-terminal message sequence or modifications of peptide conformation.

The peptide nociceptin/orphanin FQ (N/OFQ) and the N/OFQ receptor (NOP) are the last discovered member of the opioidergic system. The NOP receptor was identified from a human cDNA library on the basis of its sequence homology with classical opioid receptors^1^,^2^. Soon after, the 17-amino acid N/OFQ neuropeptide was purified from rat^3^ or porcine^4^ brain extracts and identified as the natural ligand of the NOP receptor. This was the first successful example of reverse pharmacology^5^. The N/OFQ-NOP receptor system has been demonstrated to be involved in the modulation of several peripheral and central nervous system functions including nociception, locomotion, stress and anxiety, food intake, neuroendocrine secretion, learning and memory, drug addiction, and smooth musculature tone in the cardiovascular, respiratory, gastrointestinal, and urogenital systems^6^,^7^. Despite high primary sequence homology (about 60%) between classical opioid and NOP receptors, N/OFQ activates with high affinity and selectivity the NOP receptor and opioid ligands do not interact with NOP^6^. The reasons for such distinct pharmacology of NOP compared to classical opioid receptors have been recently unraveled at atomic level since the 3D structure of NOP and opioid receptors were solved^8^,^9^,^10^,^11^. In particular, crucial structural rearrangements were evident by comparing NOP with the kappa opioid receptor where the replacement of only a few key residues in helices V and VI promoted an extensive reshaping of the binding pocket associated with an alternative coordination of water molecules^8^.

Since the beginning of modern pharmacology, G protein-coupled receptors (GPCRs) have been considered to exist and exert their biological actions as monomers. However, in the past years a growing number of studies suggested that GPCRs are able to cross-react, forming homo- and heterodimers and/or oligomers; this process might be important in modulating different receptor functions^12^,^13^,^14^.

In the opioid receptor field, evidence for delta opioid receptor homodimers^15^ as well as heterodimers (e.g. delta-kappa^16^, delta-mu^17^, kappa-mu^18^) has been reported. These studies suggested that oligomerization of opioid receptors plays a role in receptor activation and internalization and generates novel properties of ligand binding. In parallel, Portoghese and co-workers identified dimeric ligands for opioid receptor heterodimers delta-kappa^19^ (KDN series) and delta-mu^20^ (MDAN series) that were of great value for studying the biological effects associated with opioid receptor oligomerization. The KDN series was obtained combining the delta antagonist pharmacophore naltrindole and the kappa antagonist guanidinonaltrindole while the MDAN series was obtained by combining together the mu agonist oxymorphone with the delta antagonist naltrindole. Flexible spacers with length spanning from 15 to 23 atoms have been employed to link the different pharmacophores. Surprisingly, in both series of compounds the best results were obtained with compounds (KDN-21 and MDAN-21) with a spacer of 21 atoms.

As far as opioid peptide ligands are concerned, delta receptor homodimeric ligands generated using the enkephalin tetrapeptide Tyr-Gly-Gly-Phe and the opioid related sequence Tyr-D-Ala-Gly showed an increased delta receptor potency and selectivity compared with the corresponding monomers^21^,^22^. Finally, using NOP and mu receptor co-transfected cells^23^,^24^,^25^ and rat dorsal root ganglia lysate^24^ the existence of mu-NOP heteromers have been postulated. mu-NOP heterodimers might be implicated in NOP and mu receptor trafficking^24^ and can be considered as a novel pharmacological target for the development of analgesics without the classical side effects of opioid drugs^25^.

A series of peptide and non-peptide dimeric compounds were designed, synthesized and pharmacologically characterized in order to investigate the impact of ligand dimerization on NOP receptor activation. In particular, 12 peptide and 7 non-peptide dimeric ligands obtained, respectively, by dimerization of N/OFQ related sequences and of the NOP agonist 8-acenaphthen-1-yl-1-phenyl-1,3,8-triaza-spiro[4,5]decan-4-one^26^ (Ro 65-6570), are described. The novel ligands were assayed in calcium mobilization studies performed in cells coexpressing the recombinant human NOP coupled with the chimeric protein Gα_qi5_^27^ and a subset of compounds were examined in bioassay studies performed with the electrically stimulated mouse vas deferens^28^. To shed light on the unexpected recovery of activities shown by some of the developed dimers (**11** and **15**), a conformational analysis was also performed on selected peptides.

## Results

### Ligand design and synthesis

As recently and nicely reviewed^29^, the choice of the pharmacophore, the attachment point selected for linking the two pharmacophores, and the length of the spacer are crucial parameters for the design of bivalent ligands. In a first series of compounds (compounds **1**–**8**, Table 1), the smallest fragment maintaining the same potency and efficacy of the natural peptide^28^,^30^ i.e. N/OFQ(1-13) has been employed as a peptide pharmacophore. As far as the attachment point is concerned, extensive structure activity studies demonstrated that the C-terminal part of N/OFQ is an appropriate attachment point for peptide modifications^31^,^32^. Regarding the spacer length, C-terminal elongation of the N/OFQ(1-13) sequence with Gly, Ala, β-Ala, Gaba and Cys in various combinations was used to produce dimeric N/OFQ(1-13) derivatives with spacers from 18 to 32 atoms. These spacer lengths were selected in order to cover the range that has been demonstrated to be optimal for targeting putative dimeric opioid receptors^19^,^20^,^33^. The amino acids employed in the spacer were selected in order to progressively increase the spacer length by two atoms. It is worth to mention that the various spacers not only differ in length but also in flexibility.

To explore the possible differences in the pharmacological activity of monomeric vs dimeric peptide pharmacophores, in a second series of dimeric compounds the pharmacophore N/OFQ(1-13) has been shortened by C-terminal deletion of Lys^13^ and Arg^12^ (compounds **11** and **13**, Table 2). These chemical modifications have been previously reported to induce a progressive loss of peptide affinity and potency^30^,^34^,^35^. In addition, to interpret the results obtained with the aforementioned dimeric ligands, the heterodimeric derivative N/OFQ(1-12)-N/OFQ(2-12) has been synthesized (compound **15**, Table 2). In this series, the dimers contained the same 2,2′-disulfanediyldiethanamine spacer.

In order to compare chemically different NOP pharmacophores, a series of dimeric molecules, containing as NOP pharmacophore the non-peptide ligand Ro 65-6570, have been designed (compounds **19–25**, Table 3). Molecular modeling investigations^36^,^37^ suggested that NOP ligands with 1,3,8-triaza-spiro[4.5]-decane scaffold are able to bind the orthosteric binding pocket of the NOP receptor. This proposal is corroborated by pharmacological studies demonstrating that different NOP receptor antagonists displayed a competitive behavior and very similar potency when challenged against N/OFQ and Ro 65-6570^38^. The use of non-peptide ligands, which are smaller and less flexible compared to peptides, for generating dimeric compounds would permit a more precise investigation of the spacer length required for bridging the two binding pockets in a putative NOP receptor homodimer. As detailed below, Ro 65-6570 was used as racemate; we did not made efforts to resolve the two enantiomers since the original paper^26^ reporting the compound demonstrated that the difference in NOP affinity between the two enantiomers is less than ten fold (with the R isomer showing the higher affinity). For generating Ro 65-6570 dimeric ligands, the molecule was functionalized in position 3 of the 1,3,8-triaza[4.5]-spirodecane moiety with an heptanoic acid. This attachment point was selected based on previous studies demonstrating that this position can be functionalized with an acetic acid methyl ester moiety without affecting the biological activity of this class of NOP ligands^39^,^40^.

**Figure 1:**
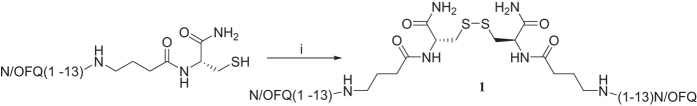
Synthesis of homodimer **1**: (i) CH_3_CN/H_2_O 50:50, NaHCO_3_ (cat), rt, 12 h. Compounds **2**–**9**, **11**, **13** were obtained under the same experimental conditions starting from the corresponding monomer.

As far as ligand synthesis is concerned, homodimeric ligands with peptide nature, compounds **1–9**, **11**, **13**, were obtained by linking via disulphide bridge two monomers in which a thiol moiety has been added at the C-terminal. Homodimeric compounds were generated by air oxidation of the monomer in the presence of NaHCO_3_ as catalyst^41^ (Fig. 1). Monomers were synthesized by solid phase peptide synthesis techniques, purified by preparative HPLC, solubilized in a mixture CH_3_CN/H_2_O 50:50 with the addition of NaHCO_3_ 5% and then stirred in an open flask at room temperature. The reaction mixture was monitored by analytical HPLC and the oxidation goes to completion in about 12 h. Under the same analytical HPLC conditions, the dimeric derivatives showed an increase in retention time of about 1.5 min compared to the parent monomer.

**Figure 2:**
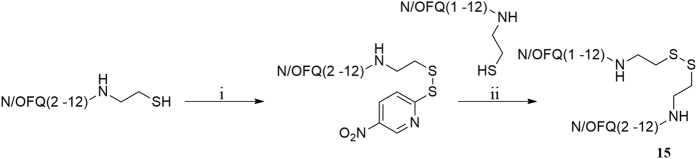
Synthesis of compound **15**: (i) 2,2′-dithiobis(5-nitropyridine), CH_3_COOH/H_2_O 3:1, rt, 5 h; (ii) 1 M ammonium acetate buffer, rt, 30 min.

Heterodimeric compound **15** was generated by using the 5-nitro-2-pyridinesulfenyl group as activating agent of the thiol function^42^. In fact, for the formation of an asymmetric disulphide bond, the activation of the thiol function of one sequence, followed by the addition of the other sequence in the free thiol form is mandatory (Fig. 2). Equimolar amount of activated and free thiol peptides were reacted in an ammonium acetate buffer solution at room temperature for 30 minutes to give the desired products in a quantitative yield.

**Figure 3:**
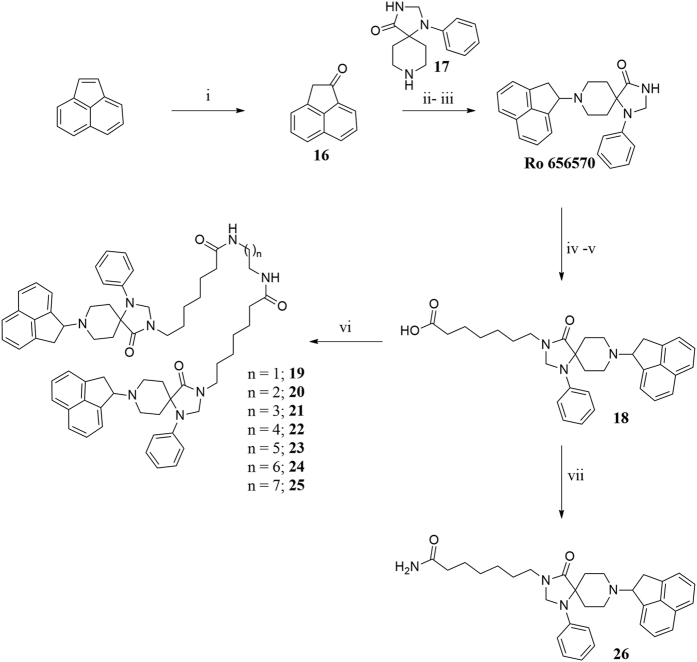
Synthesis of non-peptide homodimers 19–25: (i) *m*-chloroperbenzoic acid, CH_2_Cl_2_, rt, 24 h; (ii) Titanium (V)isopropoxide; (ii) NaBH_3_CN, EtOH, rt, 2 h; (iv) Ethyl 7-bromoheptanoate, NaH, DMF, 60 °C 2 h; (v) aq NaOH 15% w/v, Dioxane, rt, 5 h; (vi) diamine, HATU, DIPEA, DMF, rt, 5 h; (vii) NH_3_, HATU, DMF, −20 °C to rt, 12 h.

Ro 65-6570 dimeric ligands were synthesized following procedures depicted in Fig. 3. Ketone **16** was obtained by oxidation of acenaphtylene with m-chloroperbenzoic acid according with experimental conditions described in literature^43^. Reductive amination of compound **16** with the spiropiperidine **17** in the presence of titanium (IV) isopropoxide as Lewis acid and solvent provided the desired compound Ro 65-6570 that was employed in the next synthetic steps as racemate. The alkylation in position 3 of the 1,3,8-triaza[4.5]-spirodecane with an heptanoic acid moiety provided the desired arm for the further dimerization reaction. Dimerization of the NOP pharmacophore Ro 65-6570 was achieved reacting **18** with a series of linear aliphatic diamine that generated two symmetrical amide bonds and final dimeric compounds **19**–**25** with spacer length spanning from 18 to 24 atoms. Finally, intermediate **18** was also amidated (compound **26**) in order to investigate the importance of the N-3 alkylation of Ro 65-6570 for NOP binding and activation.

### Pharmacological studies

Table 1 summarized the results obtained in chinese hamster ovary (CHO_NOP_) cells coexpressing the chimeric Gα_qi5_ protein with a series of dimeric derivatives of N/OFQ(1-13)-NH_2_ characterized by a spacer length of 18–32 atoms. N/OFQ(1-13)-NH_2_ evoked a concentration dependent stimulation of calcium release displaying high potency and maximal effects (Table 1). The concentration response curve to N/OFQ(1-13)-NH_2_ was virtually superimposable to that of natural peptide N/OFQ (Table 1). Dimeric N/OFQ(1-13)-NH_2_ derivatives (compounds **1–8**) displayed maximal effects similar to N/OFQ(1-13)-NH_2_ thus behaving as NOP full agonists. The potency of these derivatives was slightly lower (3–5 fold) than that of the standard N/OFQ(1-13)-NH_2_ (Table 1). Thus the biological activity of this series of compounds is comparable to that of the standard pharmacophore and not affected by ligand dimerization, nor spacer length.

Table 2 summarized the results obtained in the calcium assay as well as in the electrically stimulated mouse vas deferens with homodimeric and heterodimeric derivatives of N/OFQ related peptides (compounds **9–15**). As already said, the N/OFQ(1-13)-NH_2_ pharmacophore displayed similar maximal effect and potency as the natural peptide both in the calcium mobilization and in the mouse vas deferens assay. The deletion of the C terminal Lys^13^ did not modify the action of the peptide in the calcium mobilization assay, although conversely causing a 27 fold loss of potency in the mouse vas deferens assay (peptide **10**). Further deletion of Arg^12^ caused a reduction of peptide potency in both assays (peptide **12**). In line with results reported in Table 1, dimerization of N/OFQ(1-13)-NH_2_ with a spacer different from those previously employed did not modify the biological activity of the peptide **9**. Similar results were obtained in the calcium mobilization assay with the dimeric derivative (compound **11**) of N/OFQ(1-12)-NH_2_ (compound **10**). However, the dimerization of the N/OFQ(1-12)-NH_2_ sequence restored full biological activity in the mouse vas deferens assay. Similar results were obtained comparing the action of compounds **13** and **12** in the calcium assay although in this case the recovery of biological activity was not complete. To investigate the mechanism by which dimerization elicits the recovery of the biological activity of N/OFQ(1-12)-NH_2_, compound **15** was synthesized and tested. This compound is generated by linking N/OFQ(1-12)-NH_2_ with the biologically inactive sequence N/OFQ(2-12)-NH_2_ (**14**). As shown in Table 2, compound **15** elicited similar effects as compound **11** in both the assays.

Table 3 summarized the results obtained in the calcium assay with Ro 65-6570 and its dimeric derivatives (compounds **19–25**). Ro 65-6570 evoked a concentration dependent stimulation of calcium release displaying high potency and maximal effects. The amidation of the heptanoic acid linked to the *N*^3^ of Ro 65-6570 (compound **26** in Fig. 3) did not modify ligand efficacy and potency. Dimeric Ro 65-6570 derivatives displayed slightly lower maximal effects compared to the standard Ro 65-6570 (Table 3). As far as potency is concerned, the most potent dimeric derivative was compound **19** that showed a small reduction in potency compared to Ro 65-6570. The increase in spacer length produced a progressive decrease in potency with compound **24** being 23 fold less potent than Ro 65-6570. It is however worth to mention that the overall impact of both ligand dimerization and spacer length on biological activity was limited.

### Conformational studies

The unexpected activity of compounds **11** and **15** could arise from conformational rearrangement of these dimeric peptides. To test this hypothesis, the conformations of N/OFQ(1-13)-NH_2_, and compounds **10**, **11**, and **15** were analyzed.

**Figure 4:**
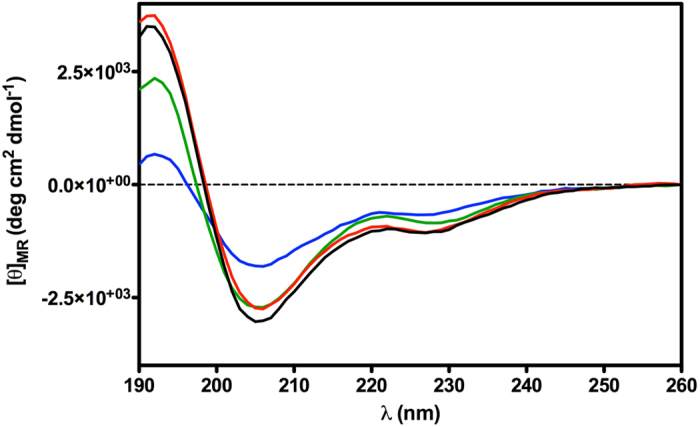
CD spectra of peptide N/OQF(1–13) (black line), peptide **10** (red line), peptide **11** (green line), peptide **15** (blue line) in SDS micelle solution.

Monomeric compounds N/OFQ(1-13)-NH_2_, and **10**, and dimeric compounds **11**, **15** were investigated by CD spectroscopy in sodium dodecylsulphate (SDS) micelle solution (Fig. 4). CD spectra of compounds N/OFQ(1-13)-NH_2_, **10** and **11** are very similar. Minima at 205 and 227 nm, and maximum at 193 nm are indicative of helical structure and minimum at 205 is also typical of turns.

In particular spectra of monomeric compounds N/OFQ(1-13)-NH_2_ and **10** are almost overlapping, while the spectrum of **11** shows some reduction of the maximum at 193 and minimum at 227 pointing to a reduced helical content. Finally, CD spectrum of the dimeric compound **15** also displays a reduction of the minimum at 205 nm, which indicates a partial loss of turn structures. Compound **11** was also investigated by solution NMR in SDS micelles. Diagnostic NMR parameters (Hα chemical shift deviations, ^3^*J*_αN_ coupling constants, dipolar couplings) ensured that the C-terminal peptide segment (from Ala^7^ to Arg^12^) prefers a helical conformation (Tables S4 and S5, and Figure S1 in the Supporting Information). Up-field shifts for Hα relative to random coil values are generally found for residues implicated in α-helix or in turn structures, and downfield shifts for those in β-sheets^44^. As shown in Figure S1, Hα atoms from residue 7 to residue 12 experience up-field shifts of the NMR signals compared to those observed for the same amino acids in random coil state. The ^3^*J*_αN_ coupling constants provide a measure of the φ angle. The ^3^*J*_αN_ values typically range between 4–6 Hz (−70 < φ < −30) for α-helices and a series of three or more coupling constants in this range are indicative of the α-helical structure^45^. ^3^*J*_αN_ coupling constants for residues 7–10 (Table S4) are in the range expected for α-helical peptides while ^3^*J*_αN_ values for residues 11 and 12 are slightly larger (6.2 and 6.8 Hz, respectively) indicating the presence of a helical structure along residues 7–12 with some degree of flexibility at the last residues (see also below). Additionally, NOE contacts of the types d_αN_(i, i + 3) between Hα of residue 8 and HN of residue 11, and between Hα of residue 9 and HN of residue 12; and d_αβ_(i, i + 3) between Hα of residue 7 and Hβ of residue 10, diagnostic for helix structure^46^, can be observed (Table S5). Temperature coefficients of amide protons (Table S4) more positive than −4.6 ppb/K for residue 9–12, predictive of a hydrogen bond^47^, are in accordance with the helical structure.

Considering the N-terminus, inter-residual NOE between Gly^2^ Hα and Phe^4^ HN is diagnostic of a β-turn structure. In fact, the distance of Gly^2^ Hα and Phe^4^ HN (3.8 Å) is typical for the turn structure, while the average distance of these types of protons within a helix is 4.4 Å and it is >5 Å in extended structures. Furthermore, the temperature coefficients of amide protons of residue 4 (Δδ/ΔT = −4.1 ppb/K) is in accordance with the turn structure.

**Figure 5:**
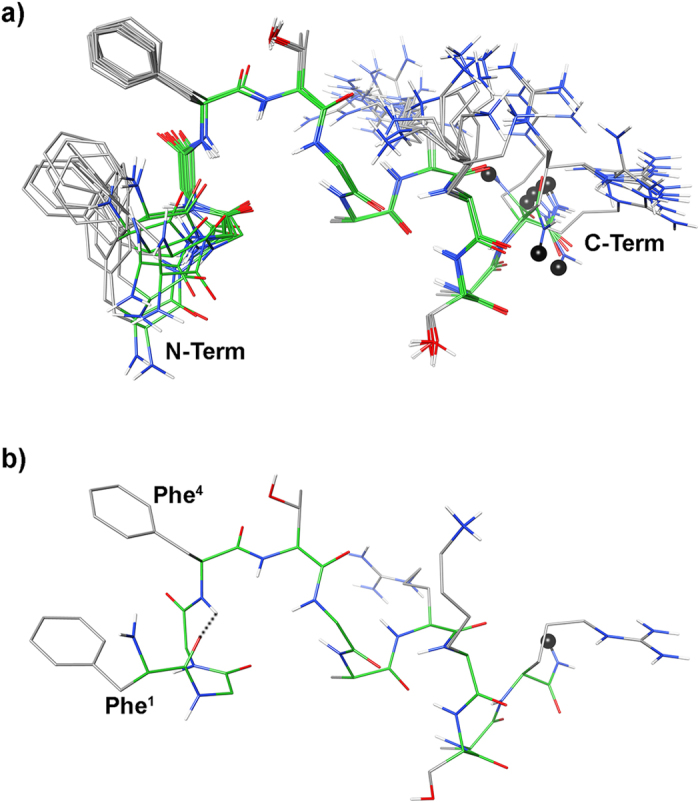
(**a**) Superposition of the ten lowest energy conformers of peptide **11**. Structure models were superimposed using the backbone heavy atoms. Spacer position is indicated by a black ball. (**b**) Lowest energy conformer of family #1 structure. H-bond in the message region was evidenced as dotted line.

Further NOE’s observed between the aromatic protons of Phe^1^ and Phe^4^, and the up-field shift of the aromatic proton signals of Phe^1^ demonstrate the spatial proximity of these aromatic rings. Using the NOE derived data as input, structure calculations by restrained simulated annealing gave the conformers of peptide **11** shown in Fig. 5. Structures display a well defined C-terminal region (backbone rmsd 0.10 Å for residues 6–12) and analysis of the ensemble dihedral angles (Table S6, Supporting Information) provides the presence of canonical α-helix from residue 7 to 10 flanked by distorted β-turns^48^ (type IV, i.e. they are not included in any canonical beta-turn structure) along residues 5–8 and 8–11.

**Figure 6:**
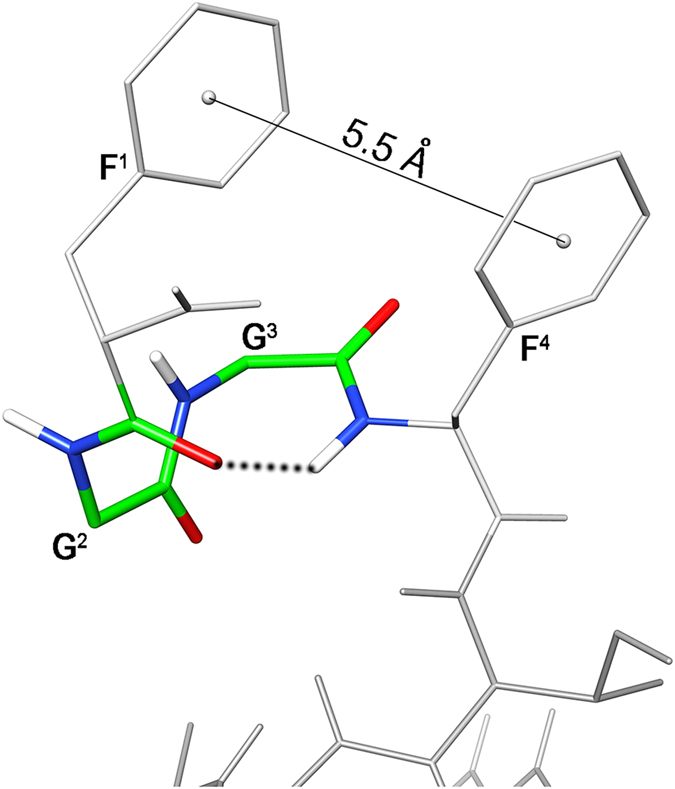
Lowest energy conformer of peptide **11** . Key atoms of the N-terminal turn structure and the distance between the aromatic ring centroids are evidenced. Hydrogen bond stabilizing the N-terminal turn is displayed as dotted line.

Small but consistent violations of some consecutive d_αN_(i,i + 1) NOEs in the helical region (Table S5) point to some degree of flexibility in this part. Residue 12 is more flexible and the following link region is completely flexible in accordance with the loss of NOE’s connecting these regions. Considering the message region (residues 1–4), about one half of the ensemble conformers displays a distorted type I β-turn with average dihedral angles of the central residues φ_i+1_ = −98 ± 18 (−60), ψ_i+1_ = −1 ± 6 (−30), φ_i+2_ = −92 ± 2 (−90), ψ_i+2_ = 26 ± 1 (0); where canonical type I β-turn^48^ are reported in parenthesis. The turn is stabilized by an H-bond between the carbonyl oxygen of residue 1 and the amide proton of residue 4 (Fig. 6). These structures are indicated as family 1 in Table S6. N-terminus of the other structures (family 2 in Table S6) is more flexible. In both families, the distance between the two phenyl rings is within 5.5 Å, considering the ring centroids, forming a π-stacking interaction (Fig. 6).

## Discussion and Conclusions

This study reports on the design, synthesis, pharmacological activity, and conformation/activity relationship of dimeric ligands of the NOP receptor. The dimeric ligands were generated using as pharmacophores both the peptide sequence of N/OFQ and the non-peptide NOP ligand Ro 65-6570. These pharmacophores were linked with spacers of different length covering the range that has been previously and successfully employed for the generation of dimeric ligands for opioid receptors. Dimerization did not modify the pharmacological activity of the pharmacophores. However, when dimeric compounds were obtained with low potency peptide pharmacophores, dimerization recovered ligand potency; these effects depend on the doubling of the C-terminal address sequence rather than the presence of an additional N-terminal message sequence or modifications of peptide conformation.

In the calcium assay the peptide (N/OFQ(1-13)-NH_2_) and non-peptide (Ro 65-6570) NOP pharmacophores displayed full agonist activity and values of potencies in line with previous studies^27^,^38^. The pharmacological activity of N/OFQ(1-13)-NH_2_ has been compared with that of a series of homodimeric derivatives (compounds **1–8**) with a spacer spanning from 18 to 32 atoms. No significant differences were measured both in terms of efficacy and potency between the dimeric derivatives and the pharmacophore. Very similar results were obtained using as NOP receptor pharmacophore the non-peptide ligand Ro 65-6570 (compounds **19–25**). These results demonstrated that the attachment points selected for generating dimeric derivatives does not affect the pharmacological activity of the pharmacophores. Of note, an increase in agonist potency was reported for opioid receptor homo^21^,^22^ and heterodimers^19^,^20^ linked with spacers of a similar length as those used in the present investigation. On the contrary, the dimeric ligands we generated displayed similar potency as the single pharmacophores. Interestingly, similar findings were previously obtained with a dimeric derivative of [Arg^14^Lys^15^]N/OFQ^49^. Collectively this evidence suggests that despite their dimeric nature these compounds bind the NOP receptor as the single pharmacophores. In other terms we were not able to provide pharmacological evidence for the presence of NOP receptor homodimers in the preparations under study.

To investigate the possibility to generate novel NOP ligands by the design of dimeric derivatives of shorter peptide pharmacophores, compounds **9–15** were synthesized and assayed. In line with previous findings^30^,^34^,^35^ shortening of N/OFQ sequence to 12 and 11 amino acids produced a progressing loss of agonist potency underlining the crucial role of Arg^12^-Lys^13^ for NOP binding. Of note, in the mouse vas deferens assay the drop in ligand potency was evident already with N/OFQ(1-12) (compound **10**) while in the calcium assay it was clear with N/OFQ(1-11) (compound **12**). This difference is most probably due to the high vs low stimulus/response coupling that characterizes the calcium vs vas deferens assay, respectively^27^. Interestingly enough, the dimerization of compounds **10** and **12** produced a recovery in ligand potency by 30 fold (compound **11**) in the vas deferens and 4 fold in the calcium assay (compound **13**). This recovery in ligand potency may in theory derive from the doubling, in the dimeric compounds, of the N-terminal (F^1^G^2^G^3^F^4^) or C-terminal (T^5^G^6^A^7^R^8^K^9^S^10^A^11^R^12^) sequences that were previously defined, in analogy with opioid studies^50^, as N/OFQ message and address, respectively^30^. This issue has been addressed by synthesizing and testing the heterodimeric compound **15** that lacks the Phe^1^ in the extra message domain; this chemical modification is known to fully eliminate NOP binding^34^,^51^,^52^. Compound **15** displayed similar efficacy and potency as compound **11** thus demonstrating the importance of the extra address rather than message sequence in the recovery of ligand potency induced by dimerization. This finding also underlines the importance of the use of negative controls (i.e. the use of an inactive pharmacophore) to deeply investigate the pharmacological activity of dimeric molecules. This allows to correctly interpret the changes in activity as due to the bivalent nature vs the overall chemical structure and/or conformation of the new compound.

Next we considered the hypothesis that the recovery of ligand potency induced by dimerization may derive from conformational reasons. To investigate this possibility the conformations of N/OFQ(1-13)-NH_2_, compounds **10**, **11**, and **15** were analyzed. Such analysis was made in SDS micelle solution. The employment of SDS micelles to investigate the conformational properties is justified on the basis of their interaction with a membrane receptor. For peptides that bind membrane receptors, such as GPCR, the use of membrane mimetic solution is suggested, hypothesizing a membrane-assisted mechanism of interactions between the peptides and their receptors^53^. According to this model, the membrane surface plays a key role in facilitating the transition of the peptide from a random coil conformation adopted in the extracellular environment to a conformation that is recognized by the receptor. The increase of the local concentration and the reduction of the rotational and translational freedom of the neuropeptide are membrane-mediated events acting as determinant steps for the conformational transition of the peptide. Hence micelle solutions have been extensively used for peptide hormones conformational studies including opioids^54^,^55^, and N/OFQ^56^,^57^.

First of all, the almost perfect overlap of the CD spectra (Fig. 4) of the pharmacophore N/OFQ(1-13)-NH_2_, and of the low active homologous N/OFQ(1-12)-NH_2_ (**10**) indicates that the drastic reduction of activity in **10** is only due to the loss of positively charged Lys^13^ residue and not to the loss of secondary structures. Moving to active homodimer **11**, it shows, from CD spectrum, similar conformation of its parent monomer **10** with some loss of helical character. Again, conformational matter cannot explain the observed change in pharmacological activity. From well-established SAR information^58^, and from direct comparison of peptides N/OFQ(1-13) and **10** it is clear that positively charged residues at C-terminus in N/OFQ analogues are mandatory. Hence, positively charged residue(s) in the dimeric part of peptides **11** and **15** are likely to be the functionality indispensable for the activity. Flexibility of the spacer should help the appropriate interactions to occur between this residue(s) and the receptor. This flexibility need is confirmed by the conformational behavior of peptide **15**. From the CD spectrum, peptide **15** loses part of the helical and turn structures while it remains as active as **11**. Clearly, the loss of the Phe^1^ from one side of the molecule is not important as far as positively charged residues are still present and correctly orientable by the flexible link.

Detailed conformational analysis of **11** by NMR revealed that address segment of the peptide is in helical conformation (Fig. 5) in accordance with previous conformational studies on the full length N/OFQ^56^,^57^. Consistent violation of a few NOE-derived distances and the comparison of the Hα chemical shift deviation of peptide **11** and N/OFQ (Figure S1) indicate that the helix is not completely stable. Regarding the N-terminal message region, all calculated structures display a π-stacking between the phenyl rings of the Phe^1^ and Phe^4^ (Fig. 6). The shielding effect of Phe^4^ on Phe^1^ is particularly evident considering the chemical shift of the aromatic protons of the last (especially H_ζ_, with Δδ ~ −0.40 ppm compared to the corresponding proton in an unshielded phenylalanine)^59^. Interestingly, similar shielding is observed for the NMR signals of N/OFQ (H_ζ_ of Phe1: Δδ ~ −0.60 ppm)^57^, nonetheless, this interaction that regards key pharmacophore residues has never been described.

Moreover considering the backbone arrangement of the message segment, two equipopulated conformational families were observed (Table S6). A distorted type I β-turn along residues 1–4 stabilized by an H-bond between carbonyl oxygen of residue 1 and amide hydrogen of residue 4 (Fig. 5b) can be observed in family 1, while nonstandard secondary structure features family 2. Whether the active conformation of N/OFQ and of its analogues is similar to the β-turn containing family 1 or family 2 or any of them, and whether it shows off the aromatic π-stacking observed in **11** has to be established by further investigations.

Collectively the conformation activity analysis described above demonstrated that the recovery of ligand potency induced by dimerization cannot be ascribed to changes in ligand conformation. Thus we propose that this recovery is due to the presence in the extra address sequence of the dimeric ligand of basic residues that possibly take the place of the Arg^12^-Lys^13^ sequence of the native peptide. In fact, molecular modeling studies^37^,^60^,^61^,^62^ demonstrated that the Arg^12^-Lys^13^ sequence forms ionic interactions with acidic residues in particular Asp195, Glu196, Glu197, Glu199 of the ELII of the NOP receptor that are crucial for ligand receptor interaction. Moreover, it is known that the addition of an extra couple of Arg-Lys in position 14 and 15 of N/OFQ sequence generate a NOP agonist more potent than the natural peptide^63^,^64^. This same chemical modification has been also applied to the NOP antagonist sequence [Nphe^1^]N/OFQ producing no change in pharmacological activity and increase in antagonist potency thus, generating UFP-101 a highly potent NOP antagonist^65^,^66^. Collectively, this evidence suggests that the ionic interaction between basic residues of the N/OFQ address and the acidic residues of the ELII of the NOP receptor are crucial for receptor occupation by peptide ligands. The detailed investigation of the NOP receptor residues interacting with the basic amino acids of peptide **11** and **15** (and more in general of N/OFQ related peptides) by docking studies is prevented by the high flexibility that characterizes these peptide sequences.

In conclusion, the present study demonstrated that peptide as well as non-peptide homodimeric NOP ligands displayed a pharmacological activity similar to the single pharmacophores. Hence, at least under the present experimental conditions, the results obtained with the present series of molecules did not provide evidence for NOP receptor homo-dimerization. Recovery of activity shown by some dimers points to the importance of the presence of positively charged residues within the address region of the ligands rather than major conformational changes of the peptide structure. The information generated by the present structure- and conformation-activity studies may be useful for the design of novel peptide NOP ligands.

## Methods

### Chemical Materials and Methods

Amino acid derivatives, reagents and solvents were purchased from Sigma Aldrich (Steinheim, Germany) or Bachem (Bubendorf, Switzerland). The purity of the tested compounds was assessed by RP-HPLC. All compounds showed >95% purity. MS analyses were performed on an ESI-Micromass ZMD 2000 or with a high resolution mass spectrometer Agilent ESI-QTOF LC/MS 6520. Chromatography was performed on Merck 230–400 mesh silica gel. ^1^H- and ^13^C-NMR data were determined in CDCl_3_ solutions with a Varian VXR 200 spectrometer or a Varian Mercury Plus 400 spectrometer. Peak positions are given in parts per million (*δ*) and *J* values are given in hertz. Splitting patterns are designed as s, singlet; d, doublet; t, triplet; q, quartet; m, multiplet; b, broad. Silica gel (Polygram SIL G/UV254) was used for thin layer chromatography. Flash chromatography was carried out on a silica gel (Merck, 230–400 Mesh). Peptides were synthesized and purified following procedures previously reported^30^. For the synthesis of compounds **9**, **11**, **13** and **15**, cysteamine 2-chlorotrityl resin has been employed as solid support.

### General procedure for the synthesis of homodimeric peptide ligands (compounds 1–9, 11, 13)

The peptide monomer (0.01 mmol) was dissolved in a 1:1 mixture of CH_3_CN/H_2_O (1 mL) and 100 μL of a 5% aqueous solution of NaHCO_3_ were added. The reaction was stirred for 12–18 h in an open flask by monitoring with analytical HPLC. After completion of the reaction, the mixture was purified by preparative HPLC to give the desired homodimer in quantitative yield. For analytical details, see Tables S1-S2 of the Supporting Information.

### Synthesis of the heterodimeric peptide 15

To a stirred solution of N/OFQ(1-12)-NH-CH_2_-CH_2_-SH (20 mg, 0.011 mmol) in a 3:1 mixture of CH_3_COOH/H_2_O (2 mL), 2,2′-dithiobis(5-nitropyridine) (17.6 mg, 0.057 mmol) was added. The suspension was stirred for 5 h at room temperature by monitoring with analytical HPLC. After completion of the reaction, the solid was filtered off and the filtrate concentrated under vacuum. The obtained residue was dissolved in 2 mL of ammonium acetate buffer (1 M), then N/OFQ(2-12)-NH-CH_2_-CH_2_-SH (18 mg, 0.011 mmol) was added. The reaction was stirred at room temperature and product formation was monitored by analytical HPLC. After 30′ the solvent was evaporated and the crude solid residue purified by preparative HPLC to give the desired heterodimer in 82% yield. For analytical details see Table S2 of the Supporting Information.

### 8-(1,2-dihydroacenaphthylen-1-yl)-1-phenyl-1,3,8-triazaspiro[4.5]decan-4-one (Ro 65-6570)

A mixture of acenaphthylen-1(2*H*)-one **16** (168 mg, 1.00 mmol) and 1-phenyl-1,3,8-triaza-spiro[4.5]decan-4-one (**17**) (277 mg, 1.20 mmol) in titanium(IV)isopropoxide (2 mL) was stirred at 60 °C for 30′ to give a red suspension. Then EtOH (20 mL) was added at the same temperature and the reaction allowed reaching room temperature under vigorous stirring. After cooling at 0 °C, NaCNBH_3_ (94 mg, 1.50 mmol) was added and the reaction stirred at room temperature for further 2 h. The yellow mixture was filtered through a celite pad and the filtrate concentrated under vacuum. The obtained residue was suspended in CH_2_Cl_2_ (150 ml) and the organic phase washed with water (3 × 25 ml), dried with Na_2_SO_4_ and evaporated to dryness. The residue was purified by flash chromatography using EtOAc/light petroleum 2:1 as eluent to obtain Ro 65-6570 (288 mg, 75%) as a yellow amorphous solid. ^1^H-NMR (CDCl_3_): *δ* 7.68 (bs, 1H), 7.63–7.20 (m, 8H), 6.87–6.89 (m, 3H), 5.01 (m, 1H), 4.72 (s, 2H), 3.60–3.38 (m, 4H), 3.17–2.90 (m, 2H), 2.65–2.49 (m, 2H), 1.75–1.63 (m, 2H). ^13^C-NMR (CDCl_3_): *δ* 178.30 (CONH), 143.31–131.32 (5 C, Ar), 129.41–115.53 (11 CH, Ar), 68.60 (CH), 60.54 (C), 59.37 (CH_2_), 47.28 (CH_2_), 43.25 (2 CH_2_), 31.39 (2 CH_2_). HRMS: [MH]^+^ calculated = 384.2070; found = 384.2063.

### 7-(8-(1,2-dihydroacenaphthylen-1-yl)-4-oxo-1-phenyl-1,3,8-triazaspiro[4.5]decan-3-yl)heptanoic acid (18)

To a stirred solution of Ro-656570 (383 mg, 1.00 mmol) in DMF (5 ml) cooled at 0 °C, NaH (100 mg, 3.00 mmol) was added in three portions. After 5′, ethyl-7-bromoheptanoate (574 μL, 2,0 mmol) was added and the solution stirred at 60 °C for 2 h. The mixture was concentrated and the residue suspended in CH_2_Cl_2_ (150 mL). The organic phase was washed with H_2_O (2 × 20 mL), dried with Na_2_SO_4_ and evaporated to dryness to give the crude ester derivative that was solubilized without further purification in 1,4-dioxane (5 mL) and NaOH 15% (5 mL). The reaction was stirred at room temperature for 5 h. The organic solvent was evaporated and the aqueous phase, acidified with HCl 6 N and extracted with CH_2_Cl_2_ (80 mL × 3). The combined organic layers were dried with Na_2_SO_4_, concentrated to dryness and the residue purified by flash chromatography using EtOAc/MeOH 9.5:0.5 as eluent to give the desired **18** as brown amorphous solid (321 mg, 63%). ^1^H-NMR (CDCl_3_): *δ* 7.78–7.72 (m, 2H), 7.63–7.55 (m, 2H), 7.47–7.43 (m, 2H), 7.30–7.28 (m, 2H), 6.95–6.93 (m, 2H), 6.82 (t, *J* = 6.8, 1H), 5.39 (m, 1H), 4.61 (s, 2H), 2.82–2.75 (m, 10H), 2.41–2.33 (m, 2H), 1.78–1.55 (m, 6H), 1.42–1.23 (m, 4H). ^13^C-NMR (CDCl3): *δ* 178.65 (COOH), 173.44 (CONH), 142.44–131.28 (5 C, Ar), 129.44–114.58 (11 CH, Ar), 67.08 (CH), 63.41 (PhCH_2_), 59.59 (C), 45.52–25.37 (11 CH_2_). HRMS: [MH]^+^ calculated = 512,29077; found = 512,29111.

### 7-(8-(1,2-dihydroacenaphthylen-1-yl)-4-oxo-1-phenyl-1,3,8-triazaspiro[4.5]decan-3-yl)heptanamide (26)

To a stirred solution of **18** (511 mg, 1 mmol) in DMF (5 mL), HATU (418 mg, 1,1 mmol) was added. The solution was cooled a −20 °C and bubbled with NH_3_ for 3 min. After 30 minutes the solution was warmed a room temperature and stirred for 12 h. The solvent was evaporated to dryness and the residue purified by flash chromatography using EtOAc/MeOH 9.5:0.5 as eluent to give **26** (368 mg, 72%) as a yellow amorphous solid. ^1^H-NMR (CDCl_3_): δ 7.82–7.35 (m, 8H), 7.15–6.55 (m, 3H), 5.47–5.30 (m, 2H), 4.69–4.68 (m, 2H), 4.12–3.54 (m, 4H). 3.50–2.80 (m, 4H), 2.21–2.17 (m, 3H), 1.64–1.59 (m, 7H), 1.43–1.30 (m, 4H). ^13^C NMR (CDCl3): δ = 174.54 (CONH_2_), 173.44 (CONH), 142.39–129.47 (5 C, Ar), 129.46–114.42 (11 CH, Ar), 67.73 (CH), 63.53 (Ph*CH*_*2*_), 58.92(C), 46.62–25.20 (11 CH_2_). HRMS: [MH]^+^ calculated = 511,306753; found = 511,30714.

### General procedure for the synthesis of homodimeric non-peptide ligands (compounds 19–25)

To a stirred solution of **18** (1.13 g, 2.2 mmol) in DMF (7 mL), HATU (836 mg, 2.2 mmol) and DIPEA (435 μL, 2.50 mmol) were added. The solution was stirred at room temperature for 15′ and then the diamine (1.0 mmol) was added. The reaction was monitored by mass spectrometry and after 5 h the solvent evaporated under vacuum. The residue was solubilized with CH_2_Cl_2_ (150 mL) and the organic phase washed with water (2 × 25 mL), dried with Na_2_SO_4_ and concentred to dryness. The residue was purified by preparative HPLC to provide the final compounds **19**–**25** as amorphous yellow solids after lyophilisation. For analytical details see Table S3 of the Supporting Information.

### CD Studies

CD spectra (Fig. 4) were recorded using a JASCO J710 spectropolarimeter at 20 °C between λ = 260–190 nm (1 mm path, 1 nm bandwidth, 4 accumulations, and 100 nm min-1 scanning speed). Measurements were performed with peptides in SDS (20 mM) solution.

### NMR Studies

The samples for NMR spectroscopy were prepared by dissolving the appropriate amount of peptide **11** in 0.45 mL ^1^H_2_O (pH 5.5), 0.05 mL ^2^H_2_O to obtain a concentration 1–2 mM of peptide, and 200 mM d_25_-SDS. NMR spectra were recorded on a Varian INOVA 700 MHz spectrometer equipped with a z-gradient 5 mm triple-resonance probe head. All the spectra were acquired at a temperature of 25 °C. The spectra were calibrated relative to TSP (0.00 ppm) as internal standard. One-dimensional (1D) NMR spectra were recorded in the Fourier mode with quadrature detection. The water signal was abolished by gradient echo^67^. 2D double quantum filtered correlated spectroscopy (DQF-COSY)^68^,^69^, total correlated spectroscopy (TOCSY)^70^, and nuclear Overhauser enhancement spectroscopy (NOESY)^71^ spectra were recorded in the phase-sensitive mode using the method from States^72^. Data block sizes were 2048 addresses in t_2_ and 512 equidistant t_1_ values. Before Fourier transformation, the time domain data matrices were multiplied by shifted sin^2^ functions in both dimensions. A mixing time of 70 ms was used for the TOCSY experiments. NOESY experiments were run with mixing times in the range of 100–200 ms. The qualitative and quantitative analyses of DQF-COSY, TOCSY, and NOESY spectra, were obtained using the interactive program package XEASY^73^. ^3^*J*_HN-Hα_ coupling constants were obtained from 1D ^1^H NMR and 2D DQF-COSY spectra. ^1^H NMR chemical shift assignments were effectively achieved according to the Wüthrich procedure^46^ (Table S4, Supporting Information).

### Structure Calculation

The NOE-based distance restraints were obtained from NOESY spectra acquired with a mixing time of 200 ms. The NOE cross peaks were integrated with the CARA program and were converted into upper distance bounds using the CALIBA program incorporated into the program package DYANA^74^. Only NOE derived constraints (Table S5, Supporting Information) were considered in the annealing procedures. NMR-derived upper bounds were imposed as semiparabolic penalty functions with force constants of 16 Kcal mol^−1 ^Å^−2^. A distance maximum force constant of 1000 Kcal/mol-1 Å^−2^ was used. Dimeric peptide **11** was built using the Insight Builder module (Accelrys Software Inc., San Diego). Atomic potentials and charges were assigned using the consistent valence force field (CVFF)^75^. The conformational space of compound was sampled through 100 cycles of restrained simulated annealing (ε = 1r). In simulated annealing, the temperature is altered in time increments from an initial temperature to a final temperature by adjusting the kinetic energy of the structure (by rescaling the velocities of the atoms). The following protocol was applied: the system was heated up to 1500 K over 2000 fs (time step = 1.0 fs); the temperature of 1500 K was applied to the system for 2000 fs (time step = 1.0 fs) with the aim of surmounting torsional barriers; successively, temperature was linearly reduced to 300 K in 1000 fs (time step = 1.0 fs). Resulting conformations were then subjected to restrained Molecular Mechanics (MM) energy minimization within Insight Discover module (ε = 1r) until the maximum RMS derivative was less than 0.001 kcal/Å, using Conjugate Gradient as minimization algorithm. Finally, conformations were subjected to 200 steps of unrestrained MM Conjugate Gradient energy minimization. From the produced 100 conformations, 10 structures, whose interproton distances best fitted NOE derived distances, were chosen for statistical analysis (Table S6, Supporting Information). The final structures were analyzed using the Insight II program (Accelrys, San Diego, CA). Molecular graphics images of the complexes were realized using the UCSF Chimera package^76^.

### Calcium mobilization assay

CHO cells stably coexpressing human recombinant NOP receptor and the C-terminally modified Gαqi5 chimeric protein were generated as previously described^27^. Cells were cultured in culture medium consisting of Dulbecco’s modified Eagle’s medium (DMEM)/HAMS F12 (1:1) supplemented with 10% fetal calf serum (FCS), penicillin (100 IU/ml), streptomycin (100 μg/ml), geneticin (G418; 200 μg/ml) and hygromycin B (100 μg/ml). Cell cultures were kept at 37 °C in 5% CO_2_/humidified air. When confluence was reached (3–4 days), cells were sub-cultured as required using trypsin/EDTA and used for experimentation. Cells were seeded at a density of 50,000 cells/well into 96-well black, clear-bottom plates. After 24 hours incubation the cells were loaded with: Hank’s Balanced Salt Solution (HBSS) supplemented with 20 mM HEPES (pH 7.4), 2.5 mM probenecid, 3 μM of the calcium sensitive fluorescent dye Fluo-4 AM and 0.01% pluronic acid, for 30 min at 37 °C. Afterwards the loading solution was aspirated and a washing step with 100 μl/well of HBSS, HEPES (20 mM, pH 7.4), 2.5 mM probenecid and 500 μM Brilliant Black was carried out. Subsequently 100 μl/well of the same buffer was added. After placing cell culture and compound plates into the FlexStation II (Molecular Devices, Sunnyvale, CA, USA), fluorescence changes were measured after 10 min of stabilization at 37 °C. On-line additions were carried out in a volume of 50 μl/well. The buffer for dilutions of the compounds is HBSS/HEPES (20 mM) or phosphate buffered solution (both containing 0.005% BSA fraction V).

### Electrically stimulated tissues

The experiments were performed on the mouse vas deferens (mVD). The tissues were taken from male CD-1 mice 16–18 g (Harlan, Ud, Italy). Mice were housed in 425 × 266 × 155 mm cages (Techniplast, Mi, Italy), 8 per cage, all under standard conditions (22 °C, 55% humidity, 12 h light/dark cycle, light on at 7:00 h), with food for mice (4RF, Mucedola, Mi, Italy) and water ad libitum. A mouse red house (Tecniplast, Va, Italy) and nesting materials were present in each cage for mice. The day of the experiment the animals were sacrificed with CO_2_ overdose. The experimental protocol was approved by the Ethical Committee for the Use of Laboratory Animals of the University of Ferrara and by the Italian Ministry of Health (authorization number 9927, 19/04/2013). The animals were treated in accordance with the European Communities Council directives (2010/63/EU) and national regulations (D.L. 26/2014). Bioassay experiments were performed as previously described^28^. The tissues were suspended in 5 ml organ bath containing Krebs solution (composition in mM: NaCl 118.5, KCl 4.7, KH_2_PO_4_ 1.2, NaHCO_3_ 25, CaCl_2_ 2.5, glucose 10). The Krebs solution was oxygenated with 95% O_2_ and 5% CO_2_. The temperature was set at 33 °C. At resting tension 0.3 g was applied to the mVD. Tissues were stimulated through two platinum electrodes with supramaximal rectangular pulse of 1 ms duration, 0.05 Hz frequency, 80 V of amplitude. The electrically evoked contractions were measured isotonically by means of Basile strain gauge transducers (Basile 7006; srl Ugo Basile, Varese, Italy) and recorder with a computer – based acquisition system (Power Lab 8, ADInstruments, Colorado Springs, USA). After an equilibration period of about 60 min, the contractions induced by electrical field stimulation were stable. At this time, cumulative concentration response curve to agonists were performed (0.5 log unit steps). A total number of 16 mice were used for the present *in vitro* studies.

### Data analysis and terminology

The pharmacological terminology adopted in this paper is consistent with IUPHAR recommendations^77^. All data are expressed as the mean ± standard error of the mean (SEM) of n experiments. For potency values 95% confidence limits were indicated (CL_95%_). In calcium mobilization experiments, maximum change in fluorescence, expressed as percent over the baseline fluorescence, was used to determine agonist response. Electrically stimulated tissues data are expressed as % of the control twitch induced by electrical field stimulation. Agonist potencies are given as pEC_50_ i.e. the negative logarithm to base 10 of the molar concentration of an agonist that produces 50% of the maximal effect of that agonist. Concentration-response curves to agonists were fitted to the classical four-parameter logistic nonlinear regression model:

Effect = Baseline + (Emax - Baseline)/(1 + 10^((LogEC_50_ – Log[compound]) HillSlope))

Curves fitting were performed using PRISM 6.0 (GraphPad Software In., San Diego, USA).

## Additional Information

**How to cite this article**: Pacifico, S. *et al*. Structure- and conformation-activity studies of nociceptin/orphanin FQ receptor dimeric ligands. *Sci. Rep.* **7**, 45817; doi: 10.1038/srep45817 (2017).

**Publisher's note:** Springer Nature remains neutral with regard to jurisdictional claims in published maps and institutional affiliations.

## Supplementary Material

Supplementary Information

## Figures and Tables

**Table 1:** Effects of N/OFQ(1-13)-NH_2_ and its dimeric derivatives in calcium mobilization studies performed on CHO cells coexpressing the NOP receptor and the Ga_qi5_ chimeric protein.

cpd	Structure	Length of Spacer	pEC_50_ (CL_95%_)	E_max_ ± SEM
—	N/OFQ		9.43 (9.07–9.79)	155 ± 19
—	N/OFQ(1–13)-NH_2_		9.60 (9.40–9.79)	163 ± 32
1	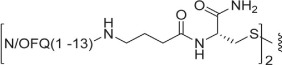	18	9.01* (9.19–8.83)	171 ± 63
2	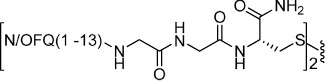	20	8.90* (9.15–8.66)	153 ± 44
3	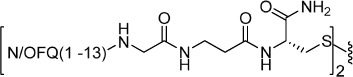	22	9.10* (9.38–8.81)	179 ± 63
4	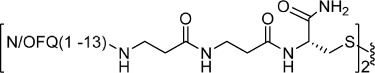	24	9.10* (9.22–8.98)	171 ± 30
5	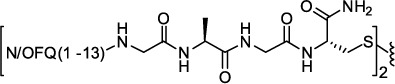	26	9.15* (9.32–8.97)	156 ± 23
6	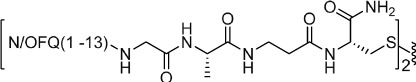	28	9.15* (9.34–8.96)	162 ± 62
7	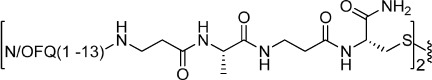	30	9.04* (9.27–8.80)	161 ± 48
8	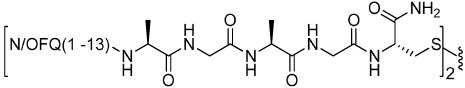	32	9.14* (9.33–8.96)	172 ± 46

Data are the mean ± SEM of at least 4 separate experiments made in duplicate. E_max_ values are expressed as fluorescence intensity in percent over the basal values. *P < 0.05 versus N/OFQ(1-13)-NH_2_ according to ANOVA followed by the Dunnett’s test for multiple comparisons.

**Table 2:** Effects of N/OFQ(1-13)-NH_2,_ C-terminal truncated derivatives, and their homo and heterodimers in calcium mobilization studies performed on CHO cells coexpressing the NOP receptor and the Ga_qi5_ chimeric protein and in the electrically stimulated mouse vas deferens bioassay.

cpd	Structure	Ca^2+^mobilization		mVD	
		pEC_50_ (CL_95%_)	E_max_ ± SEM	pEC_50_ (CL_95%_)	E_max_ ± SEM
—	N/OFQ	9.70 (9.49–9.91)	200 ± 22%	7.11 (7.00–7.22)	87 ± 5%
—	N/OFQ(1-13)-NH_2_	9.83 (9.51–10.16)	199 ± 20%	7.02 (6.94–7.10)	89 ± 3%
9	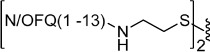	9.40 (9.15–9.65)	213 ± 24%	7.31 (7.14–7.49)	88 ± 3%
10	N/OFQ(1-12)-NH_2_	9.70 (9.41–9.98)	204 ± 16%	5.59* (5.22–5.95)	60 ± 4%^a^
11	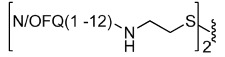	9.38* (9.06–9.70)	205 ± 18%	7.27 (7.09–7.45)	84 ± 5%
12	N/OFQ(1-11)-NH_2_	8.21* (8.11–8.32)	182 ± 34%	5.28* (5.05–5.51)	65 ± 5%^a^
13	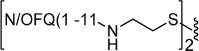	8.87* (8.58–9.16)	193 ± 22%	5.93* (5.81–6.05)	85 ± 4%
14	N/OFQ(2-12)-NH_2_	Inactive		inactive	
15	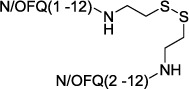	9.49 (9.07–9.91)	205 ± 27%	7.31 (7.18–7.44)	92 ± 3%

Data are the mean ± SEM of at least 4 separate experiments made in duplicate. E_max_ values are expressed as fluorescence intensity in percent over the basal values for calcium mobilization experiments and as percent of the control twitch for mouse vas deferens experiments. *P < 0.05 versus N/OFQ(1-13)-NH_2_ according to ANOVA followed by the Dunnett’s test for multiple comparisons^a^.The concentration response curve was incomplete due to the low potency of the compound.

**Table 3:** Effects of Ro 65-6570 and its dimeric derivatives in calcium mobilization studies performed on CHO cells coexpressing the NOP receptor and the Gα_qi5_ chimeric protein.

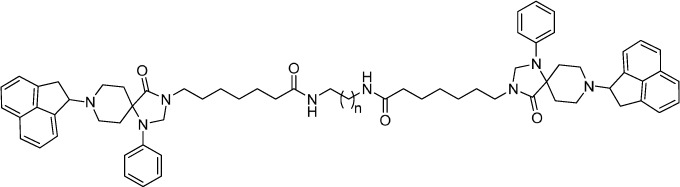				
compound	n	Spacer length	pEC_50_(CL_95%_)	E_max_ ± SEM
Ro 65-6570	—	—	8.21 (8.01 – 8.40)	165 ± 14
**19**	1	18	7.57 (7.23–7.91)	131 ± 12
**20**	2	19	7.45* (7.18–7.73)	125 ± 6
**21**	3	20	7.29* (7.00–7.58)	135 ± 20
**22**	4	21	7.14* (6.71–7.58)	143 ± 18
**23**	5	22	6.99* (6.91–7.08)	132 ± 14
**24**	6	23	6.85* (5.81–7.89)	150 ± 35
**25**	7	24	7.14* (6.30–7.99)	131 ± 21
**26**	—	—	8.24 (7.84–8.65)	143 ± 9

Data are the mean ± SEM of at least 4 separate experiments made in duplicate. E_max_ values are expressed as percent fluorescence intensity over the basal values. *P < 0.05 versus N/OFQ(1-13)-NH_2_ according to ANOVA followed by the Dunnett’s test for multiple comparisons.
