# Supplementary material for: Structure- and conformation-activity studies of nociceptin/orphanin FQ receptor dimeric ligands
Source: Sci Rep. 2017 Apr 6;7:45817. doi: 10.1038/srep45817 (PMC5382891; doi:10.1038/srep45817)
Supplement: Supplementary Information [file srep45817-s1.pdf]

SUPPORTING INFORMATION

Structure- and conformation-activity studies of nociceptin/orphanin FQ receptor dimeric ligands

Salvatore Pacifico, Alfonso Carotenuto, Diego Brancaccio, Ettore Novellino, Erika Marzola, Federica Ferrari, Maria Camilla Cerlesi, Claudio Trapella, Delia Preti, Severo Salvadori, Girolamo Calò and Remo Guerrini

Table of contents

1. HPLC method for the assessment of final purity of final compounds (page S2).
2. Analytical Data of Compounds **1-8** (page S2, Table S1).
3. Analytical Data of Compounds N/OFQ(1-13)-NH_2 and **9-15** (page S3, Table S2).
4. Analytical Data of Compounds RO 65-6570 and **19-25** (page S3, Table S3).
5. HPLC chromatograms and HRMS mass spectra of final compounds of Tables 1-3 (pages S4-S53).
6. NMR Spectra of Compound RO 65-6570 (pages S23-S24)
7. NMR Spectra of Compound **18** (pages S27-S28)
8. NMR Spectra of Compound **26** (pages S31-S32)
9. NMR Resonance Assignments of Peptide **11**, in SDS-d_{25} 200 mM Solution (page S54, Table S4).
10. NOE derived upper limit constraints for peptide **11** (page S55, Table S5).
11. Torsion angles of peptide **11** conformers (page S59, Table S6).
12. Secondary shifts of the α protons of peptide **11** in SDS solution (page S61, Figure S1).

HPLC method for the assessment of final purity of final compounds.

Analytical HPLC analyses were performed on a Beckman 126 liquid chromatograph equipped with a Beckman 168 diode array detector. Analytical purity of the peptides were determined using a Luna C_{18} column (4.6 x 100 mm, 3 μ particle size) with solvents A (0.01% aqueous TFA) and solvent B(60% CH_3CN, 0.01% TFA) programmed at a flow rate of 0.5 mL/min with a linear gradient from 0% to 50% B over 25 min. All analogs showed >95% purity when monitored at 220 nm. Molecular weights of final compounds were determined by a mass spectrometer ESI Micromass ZMD-2000 or by a high resolution mass spectrometer Agilent ESI-QTOF LC/MS 6520.

Retention times (t_R), HPLC purity and calculated and found MH^+ are listed in Tables S1-S3.

Table S1. Analytical Data of Compounds **1-8**.

**Compound**	**t_R**	**HPLC purity**	**MH^+ calculated**	**MH^+ found**
N/OFQ(1-13)-NH_2	10.317	>99.9	1382.59	1382.66
**1**	12.583	>99.9	3138.65	3138.42
**2**	12.200	>99.9	3196.65	3195.30
**3**	12.167	99.2	3224.70	3225.56
**4**	12.033	>99.9	3252.75	3253.52
**5**	12.217	>99.9	3338.80	3340.44
**6**	12.683	>99.9	3366.85	3367.55
**7**	12.550	96.6	3394.91	3395.60
**8**	12.900	>99.9	3480.96	3480.13

Table S2. Analytical Data of Compounds N/OFQ(1-13)-NH_2 and **9-15**.

Compound	t_R	HPLC purity	MH^+ calculated	MH^+ found
N/OFQ(1-13)-NH_2	10.317	>99.9	1382.59	1382.66
**9**	13.000	97.3	2882.39	2881.51
**10**	10.633	97.9	1254.41	1254.56
**11**	13.350	99.3	2626.05	2625.71
**12**	11.150	>99.9	1098.23	1098.31
**13**	13.817	96.8	2313.67	2312.95
**14**	10.767	>99.9	1107.24	1107.86
**15**	12.250	96.4	2478.87	2479.77

Table S3. Analytical Data of Compounds RO 65-6570 and **19-25**.

Compound	t_R	HPLC purity	MH^+ calculated	MH^+ found
RO 65-6570	14.950	97.3	384.2070	384.2063
**19**	15.333	98.2	1047.6219	1047.6216
**20**	15.700	95.4	1061.6375	1061.6365
**21**	16.150	99.2	1075.6532	1075.6454
**22**	16.583	>99.9	545.3381*	545.3391*
**23**	16.667	96.9	1103.6845	1103.6827
**24**	16.917	>99.9	1117.7001	1117.6976
**25**	17.100	99.1	1131.7158	1131.7127

*in this case are reported the calculated and found $[M+2H]^{2+}$

N/OFQ(1-13)-NH_2

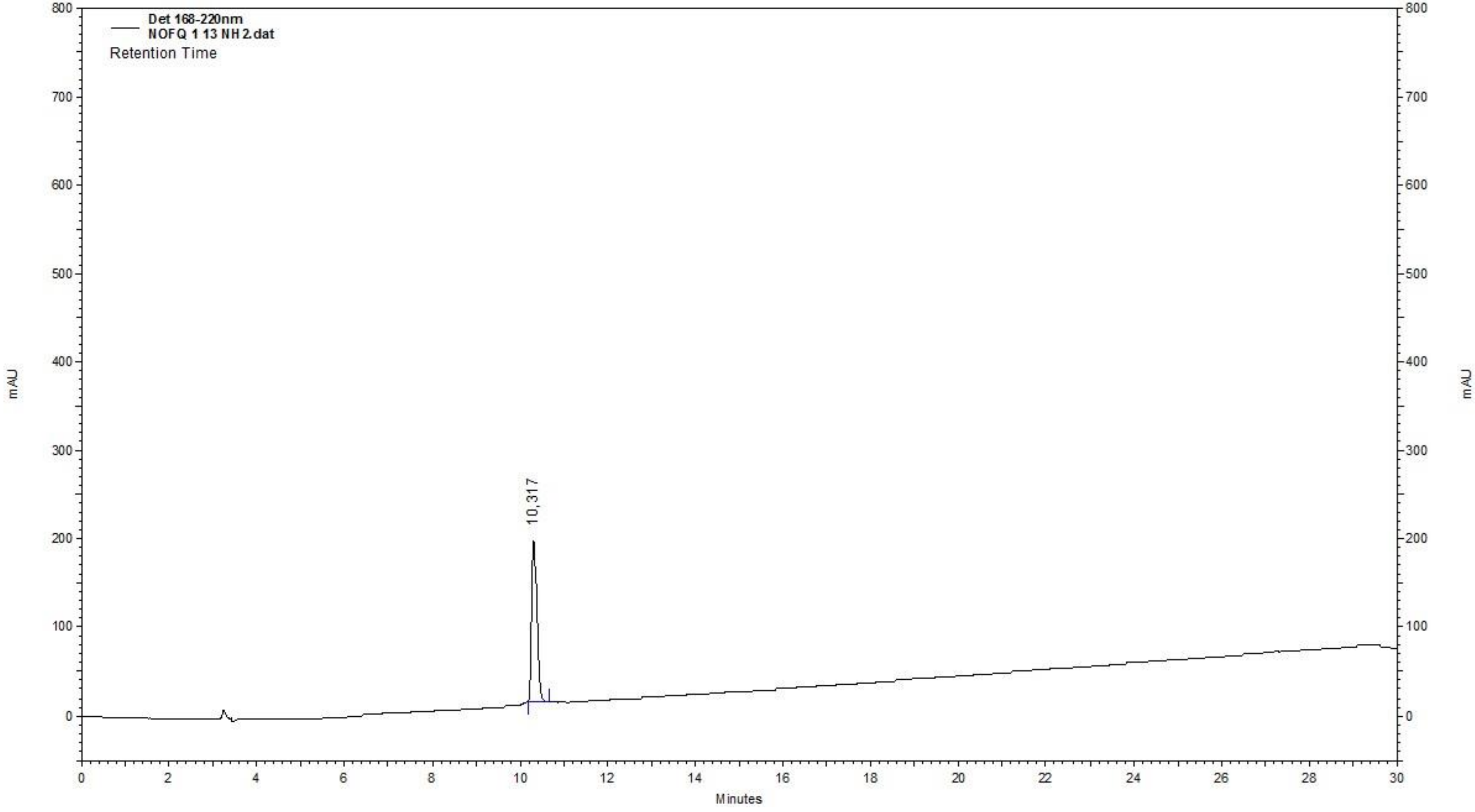
Det 168-220nm
NOFQ 1 13 NH 2.dat
Retention Time
10,317
mAU
mAU
Minutes
800
700
600
500
400
300
200
100
0
0
2
4
6
8
10
12
14
16
18
20
22
24
26
28
30

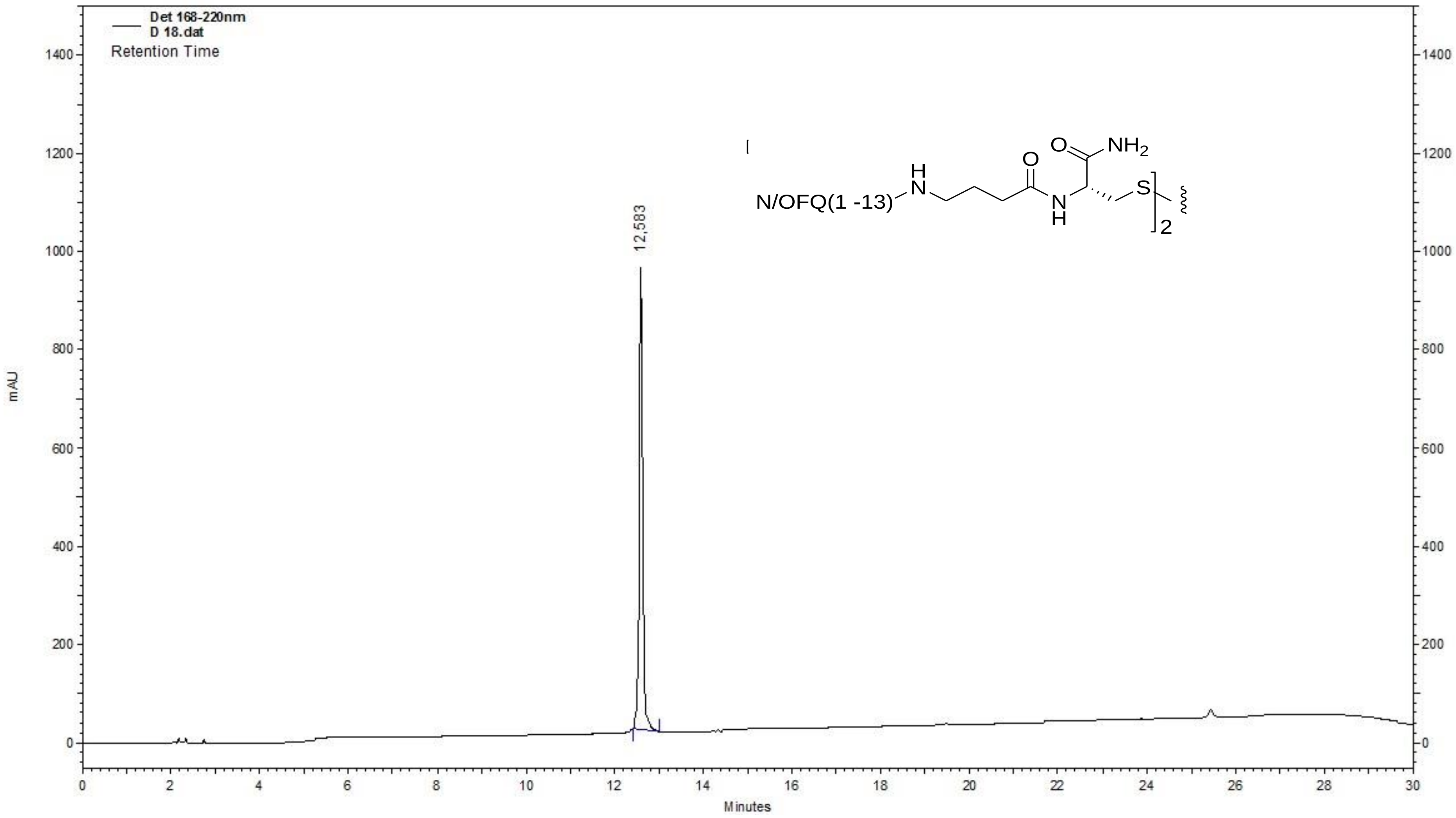
Det 168-220nm
D 18.dat
Retention Time
N/OFQ(1 -13)
H
N
O
NH2
O
N
H
S
2
12,583
mAU
Minutes
0
200
400
600
800
1000
1200
1400
0
2
4
6
8
10
12
14
16
18
20
22
24
26
28
30

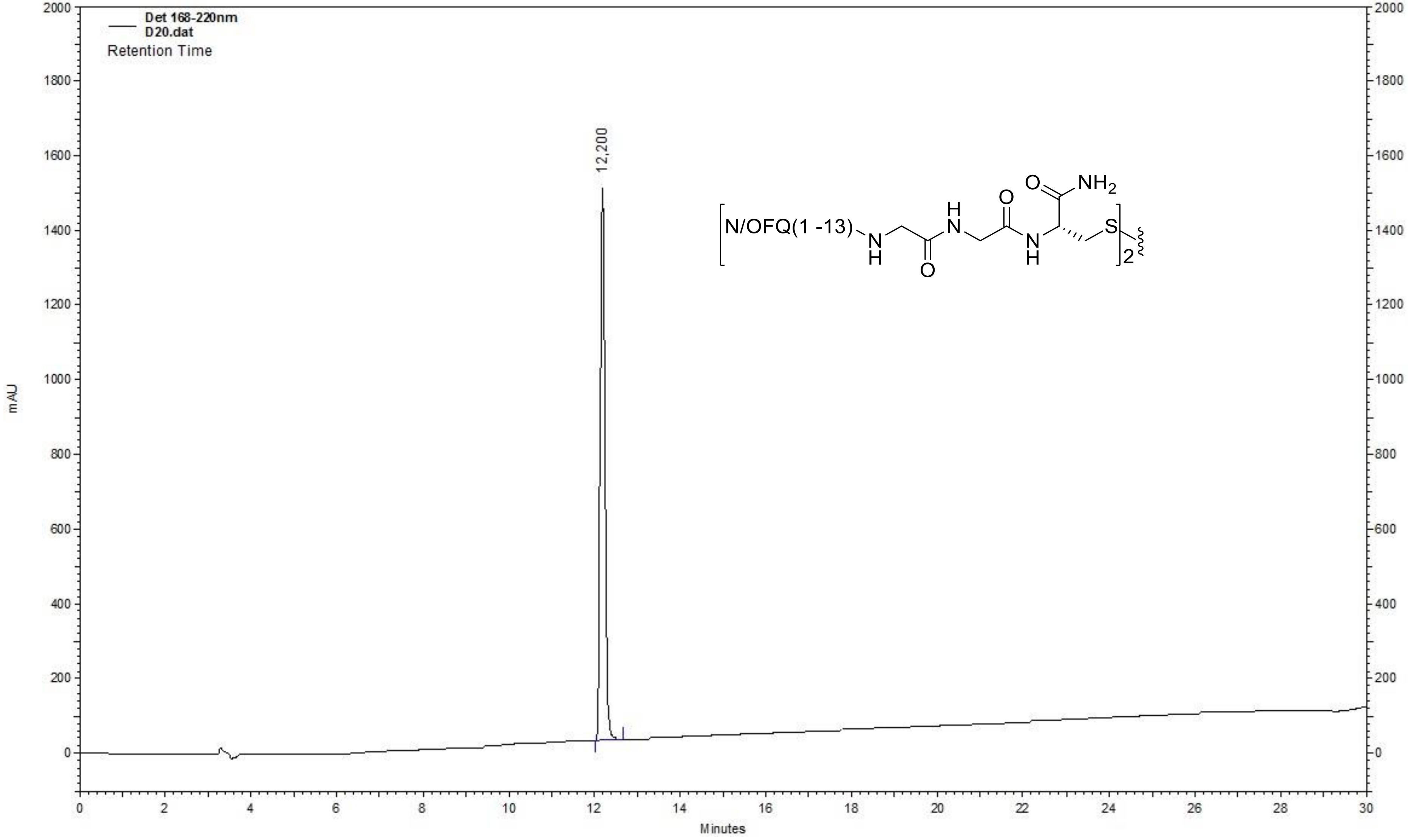
Det 168-220nm
D20.dat
Retention Time
12,200
mAU
Minutes
N/OFQ(1 -13)
NH₂
S
2

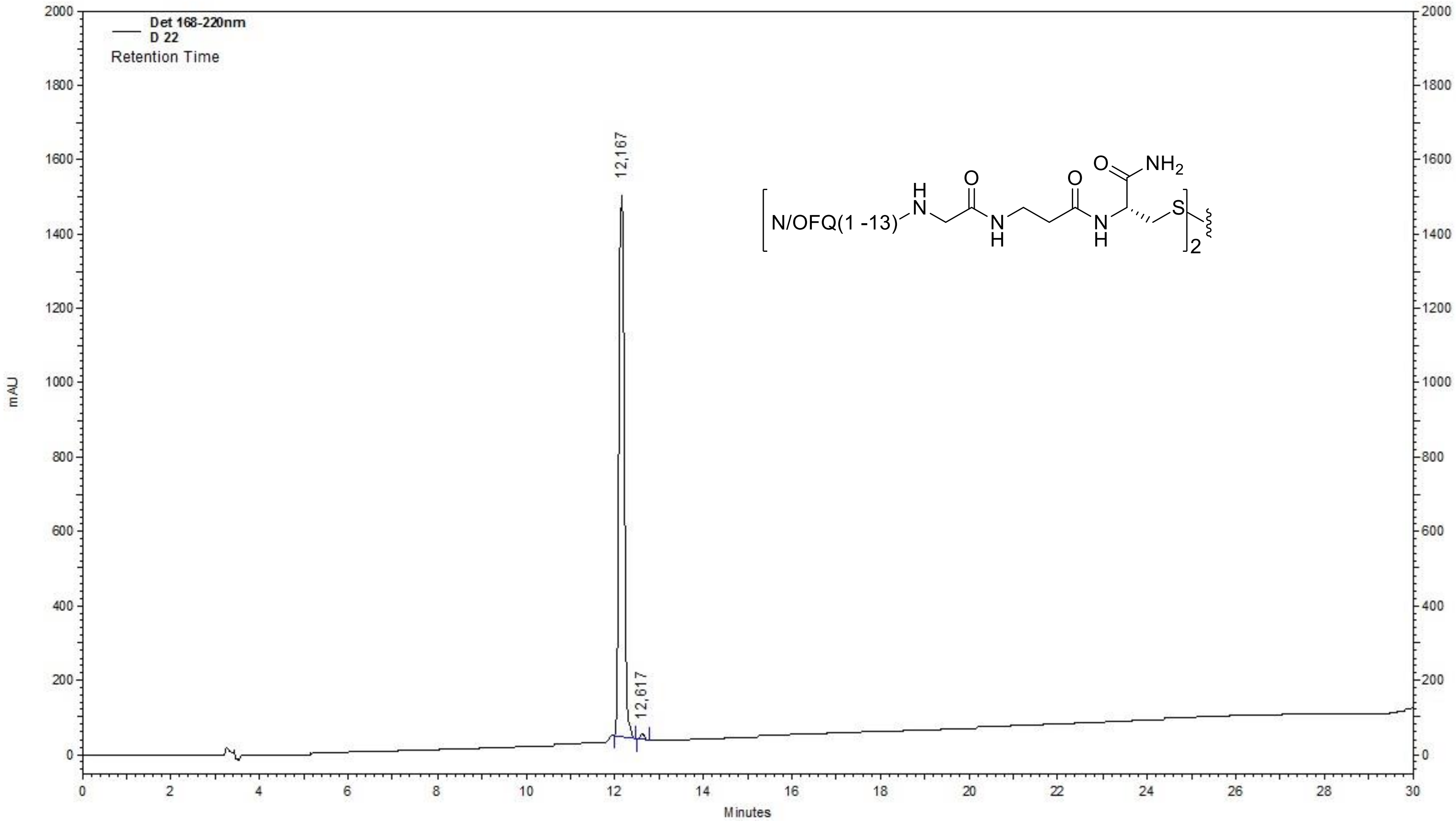

Det 168-220nm
D 22
Retention Time
12,167
12,617
mAU
Minutes
N/OFQ(1 -13)
NH2
S
2

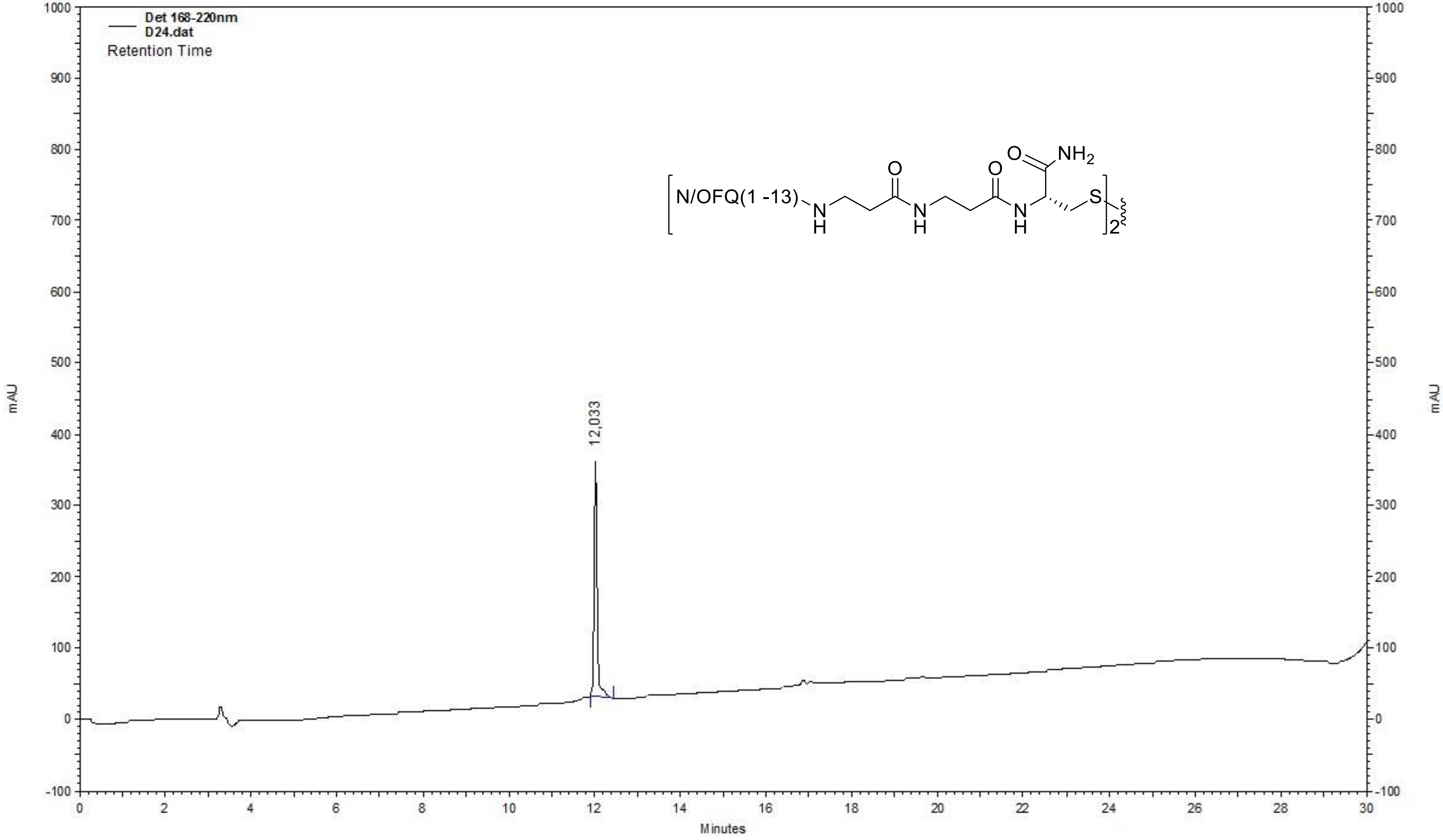
Det 168-220nm
D24.dat
Retention Time
12,033
mAU
Minutes
N/OFQ(1 -13)
NH$_2$

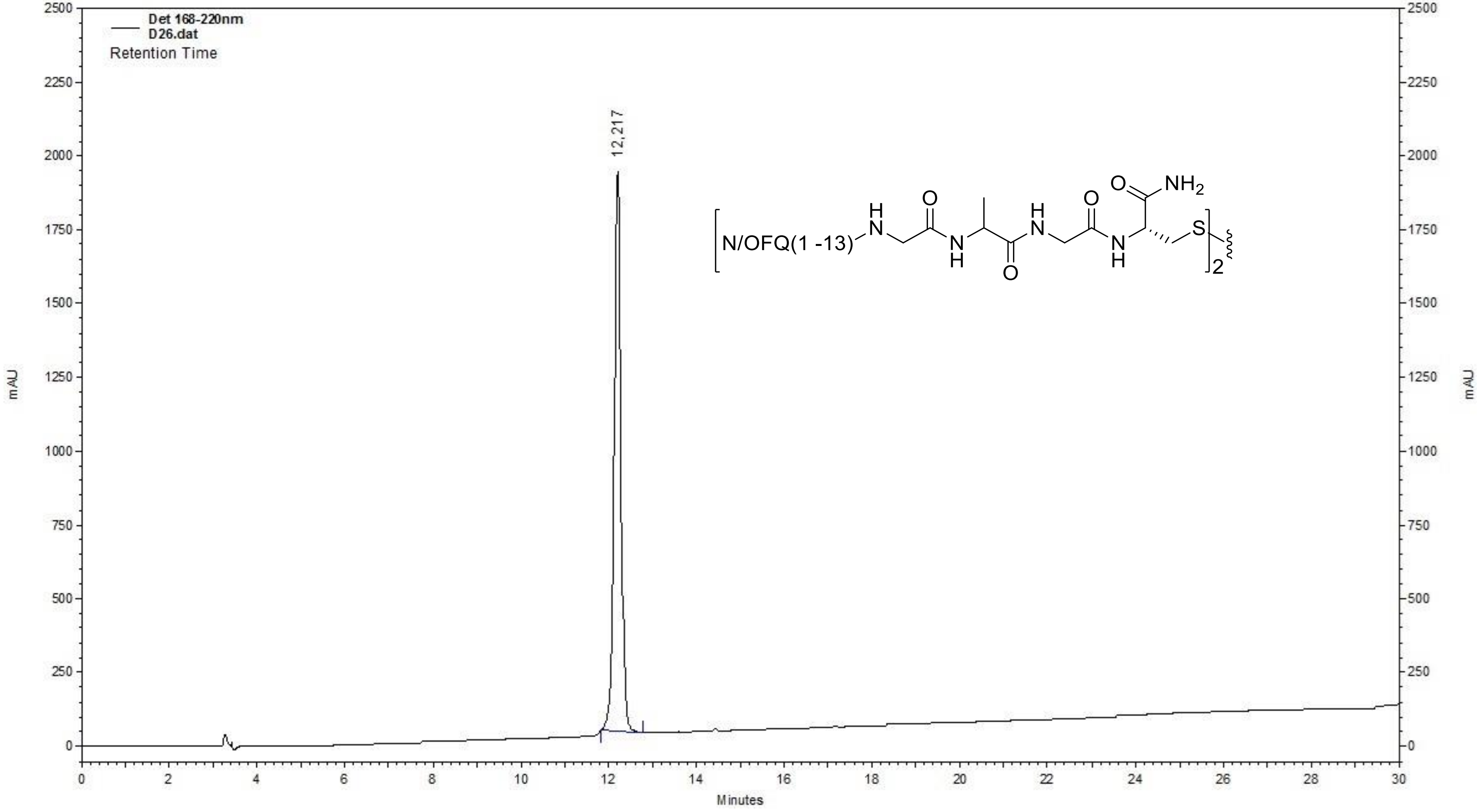

Det 168-220nm
D26.dat
Retention Time
12,217
N/OFQ(1 -13)
NH2
S
2
mAU
Minutes

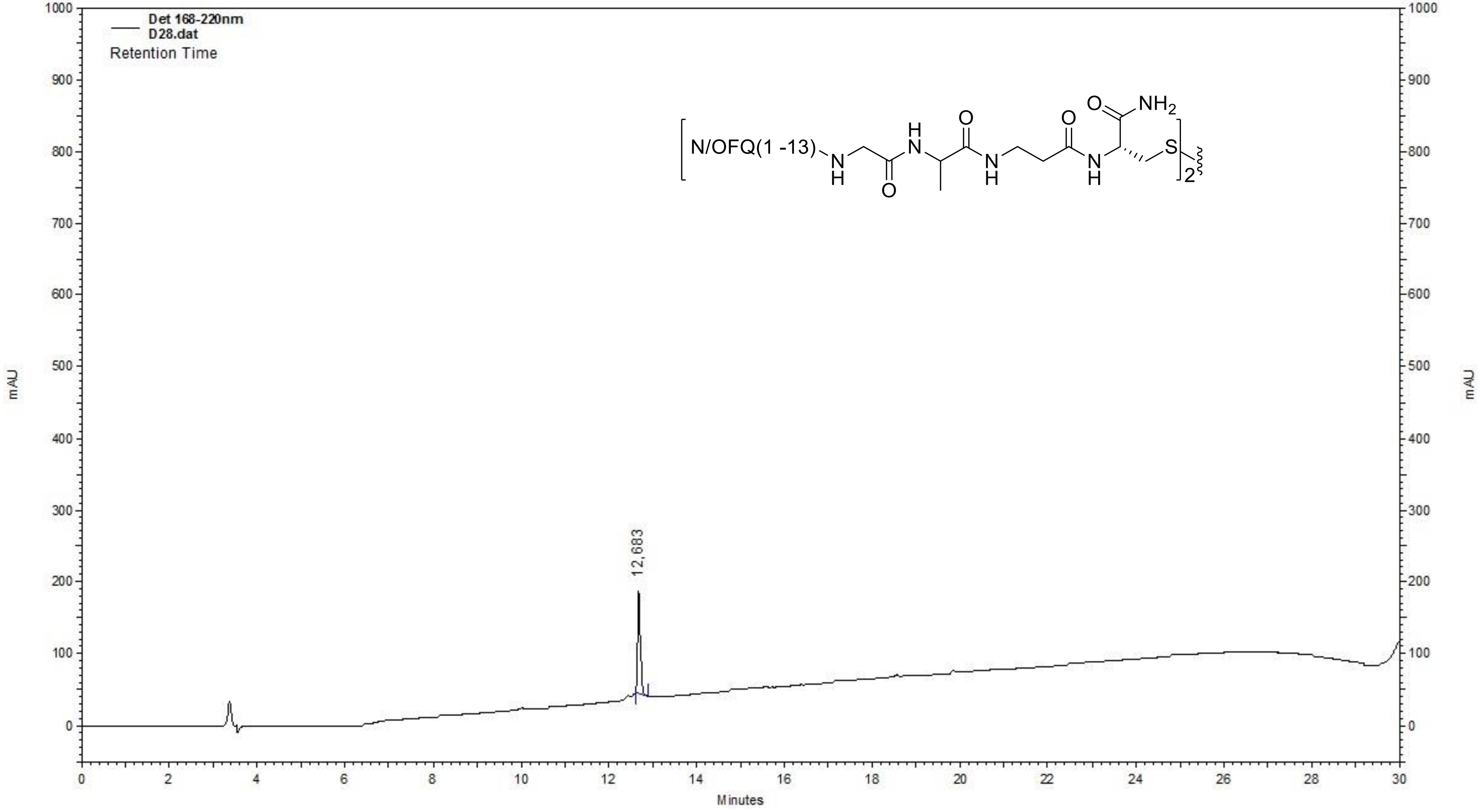
Det 168-220nm
D28.dat
Retention Time
N/OFQ(1 -13)
NH2
S
2
12,683
mAU
Minutes

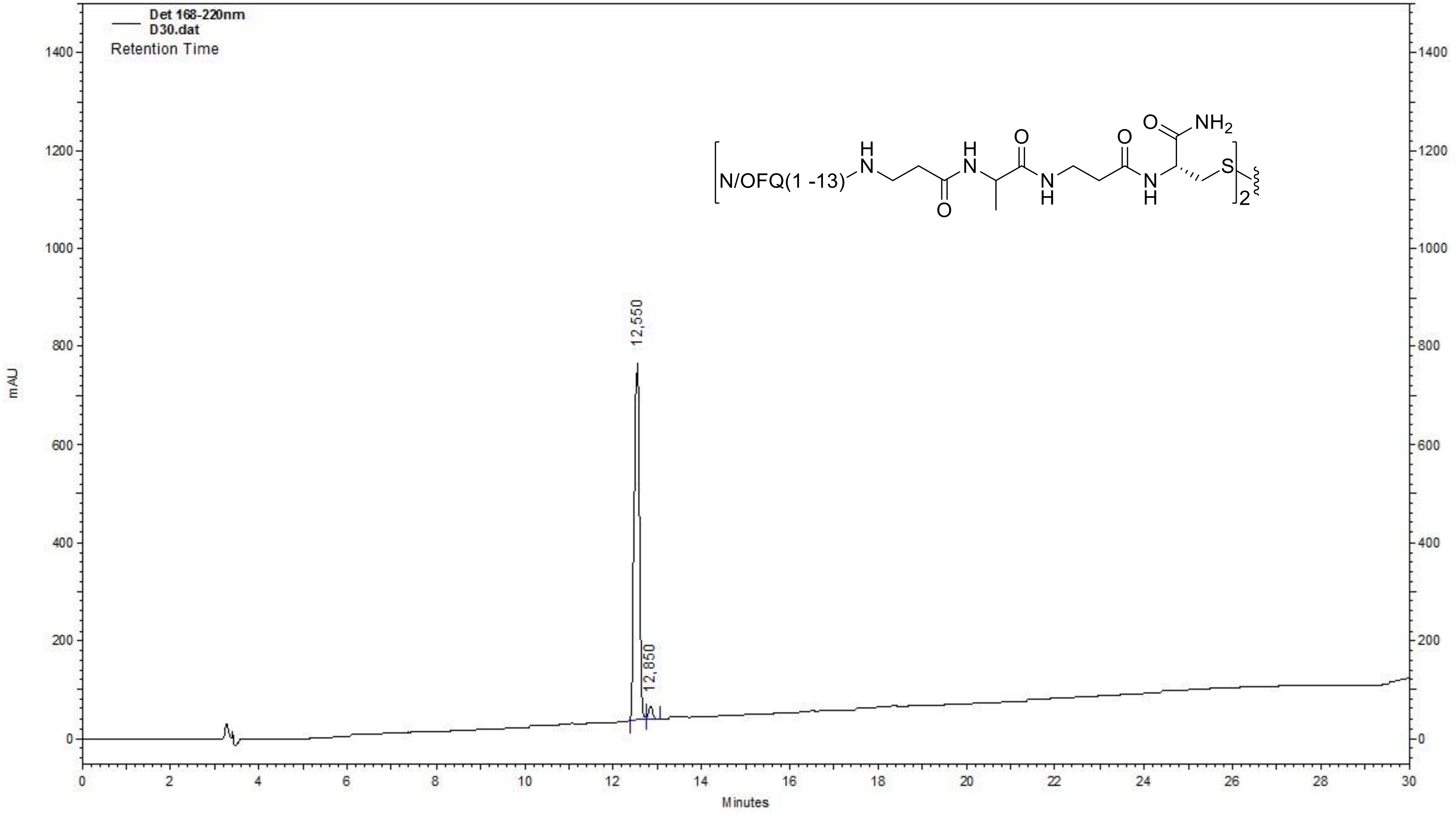

Det 168-220nm
D30.dat
Retention Time
N/OFQ(1 -13)
NH2
2
12,550
12,850
mAU
Minutes

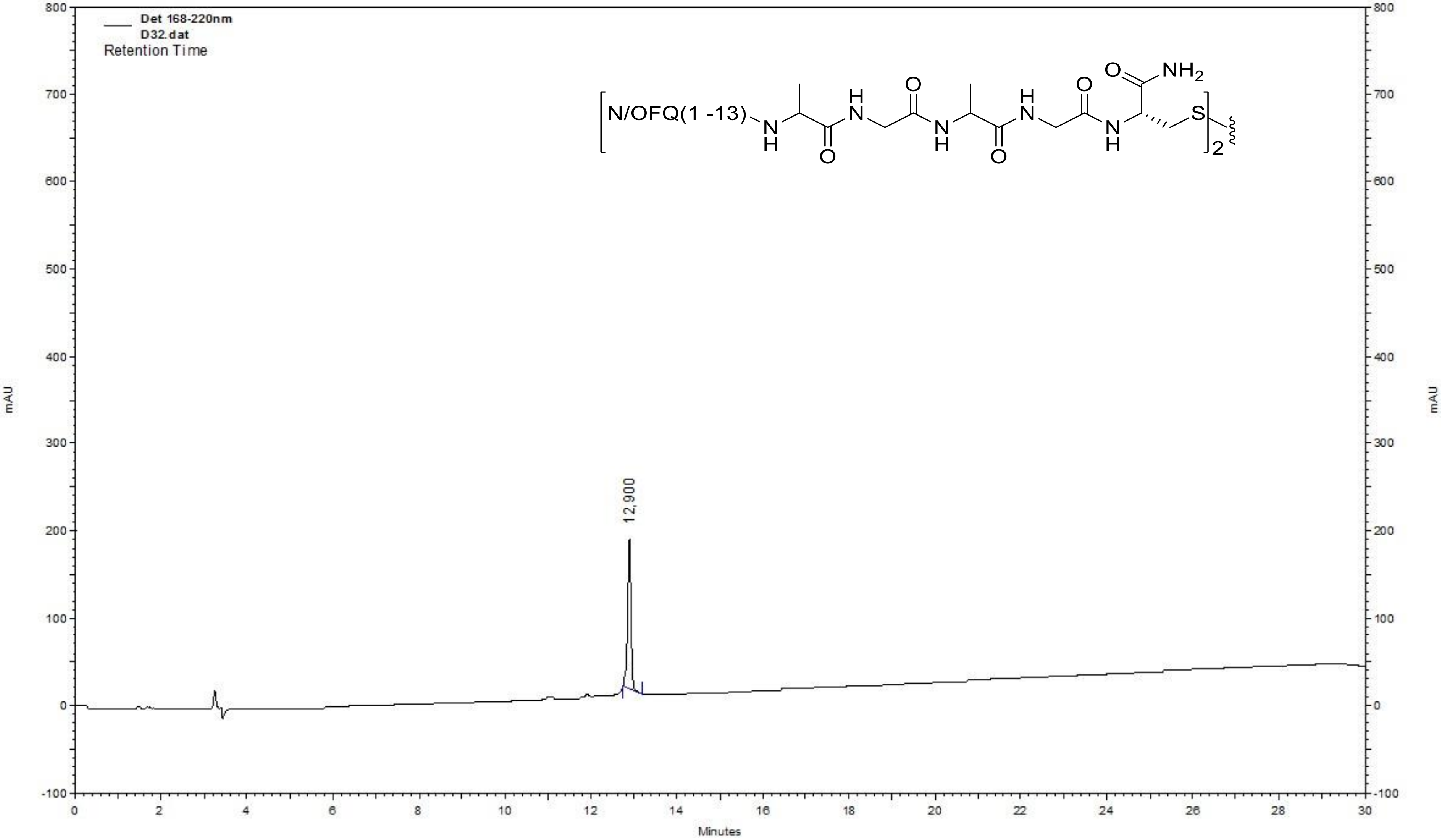

Det 168-220nm
D32.dat
Retention Time
N/OFQ(1 -13)
NH2
12,900
mAU
Minutes

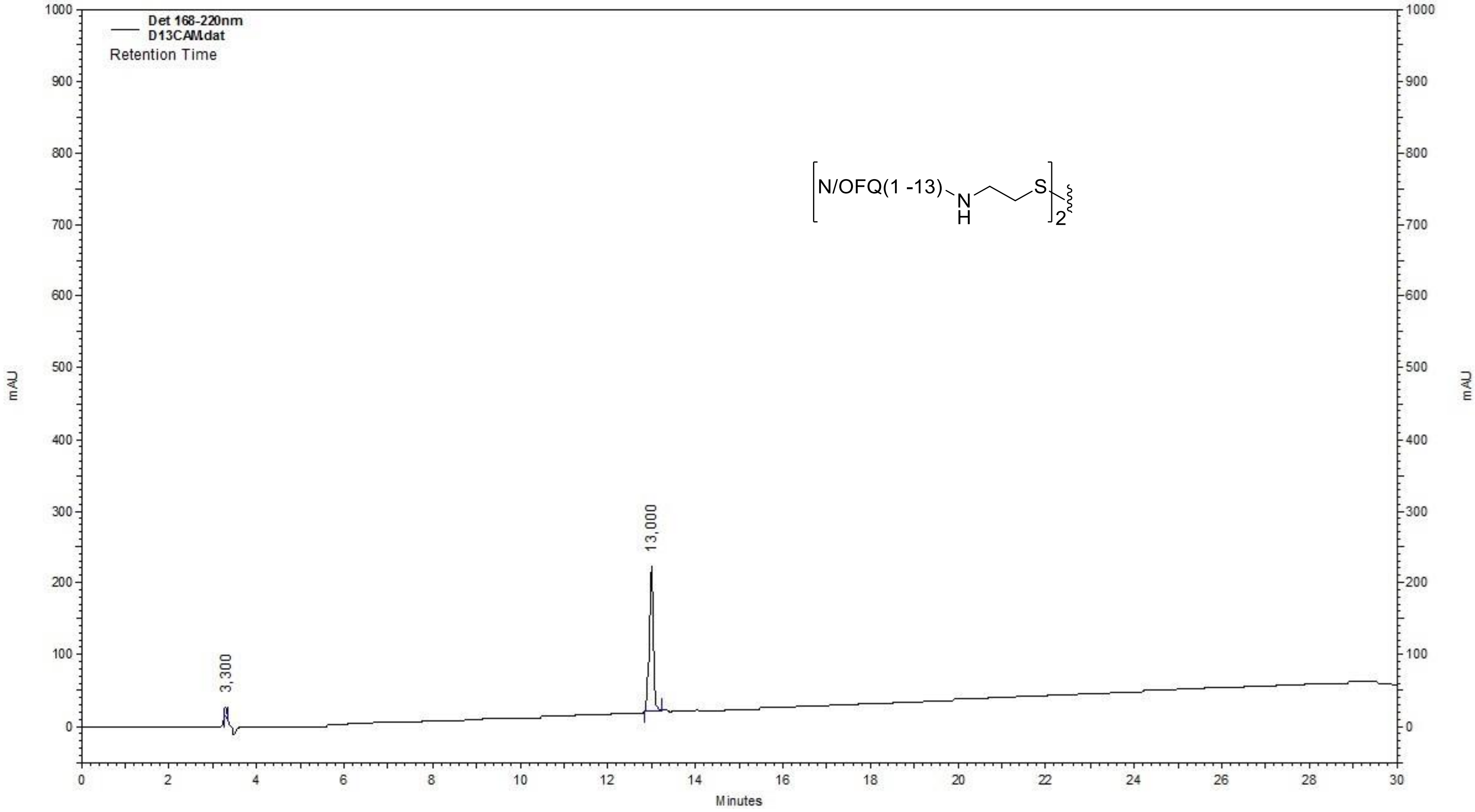

Det 168-220nm
D13CAM.dat
Retention Time
N/OFQ(1 -13)
N
H
S
2
3,300
13,000
mAU
Minutes

N/OFQ(1-12)-NH_2

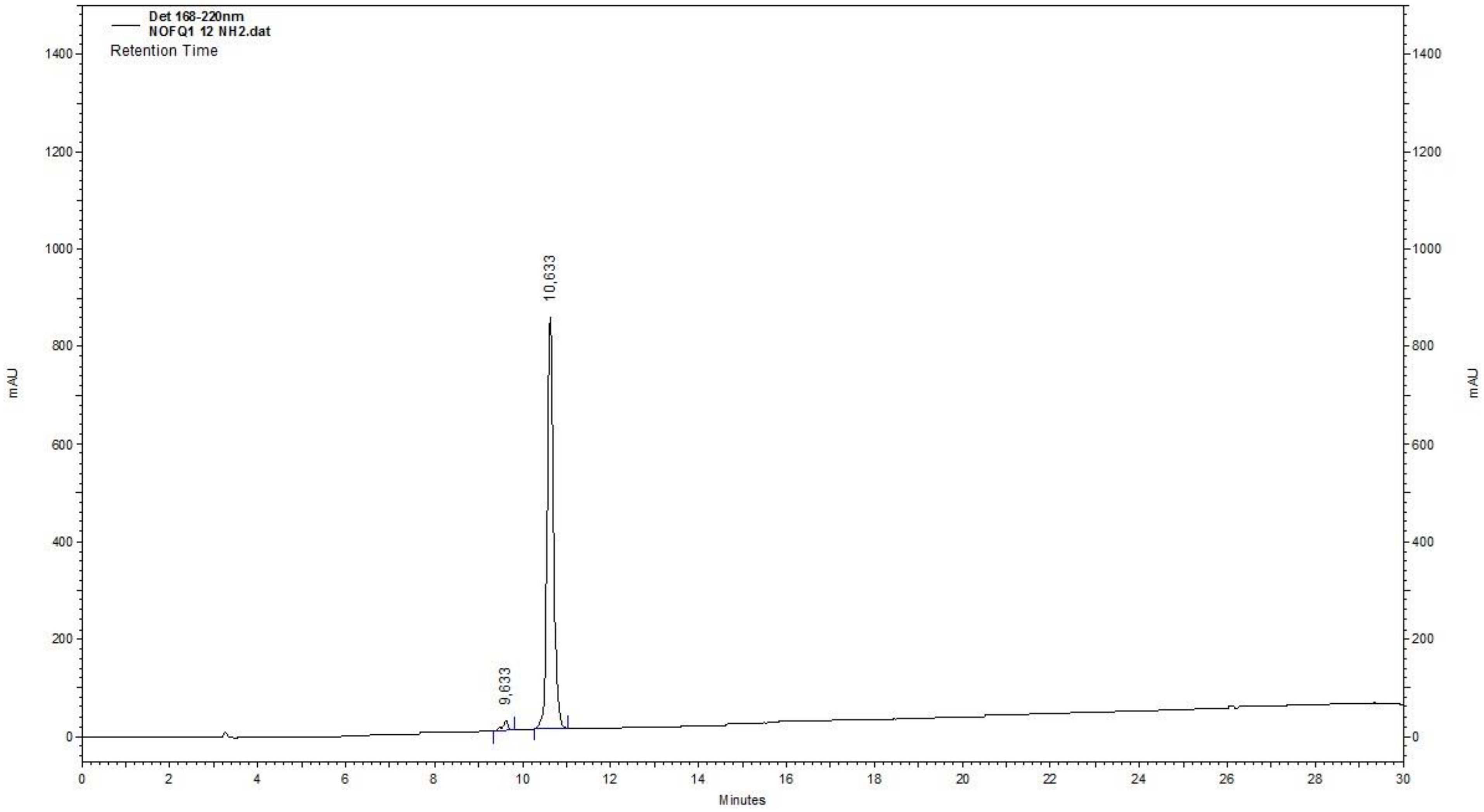

Det 168-220nm
NOFQ1 12 NH2.dat
Retention Time
10,633
9,633
mAU
mAU
Minutes
0
2
4
6
8
10
12
14
16
18
20
22
24
26
28
30
1400
1200
1000
800
600
400
200
0

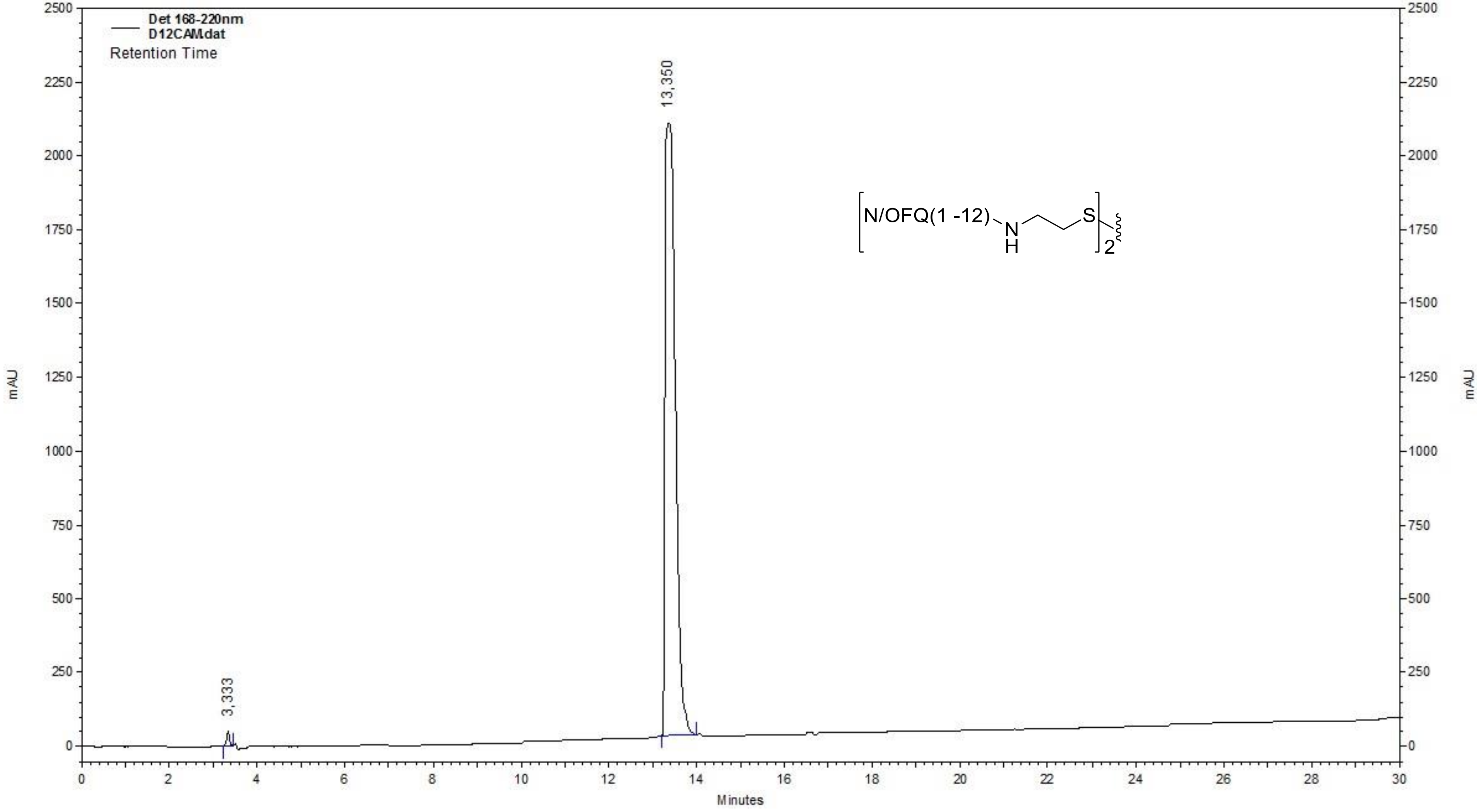

Det 168-220nm
D12CAM.dat
Retention Time
13,350
3,333
N/OFQ(1 -12)
N
H
S
2
mAU
Minutes

N/OFQ(1-11)-NH_2

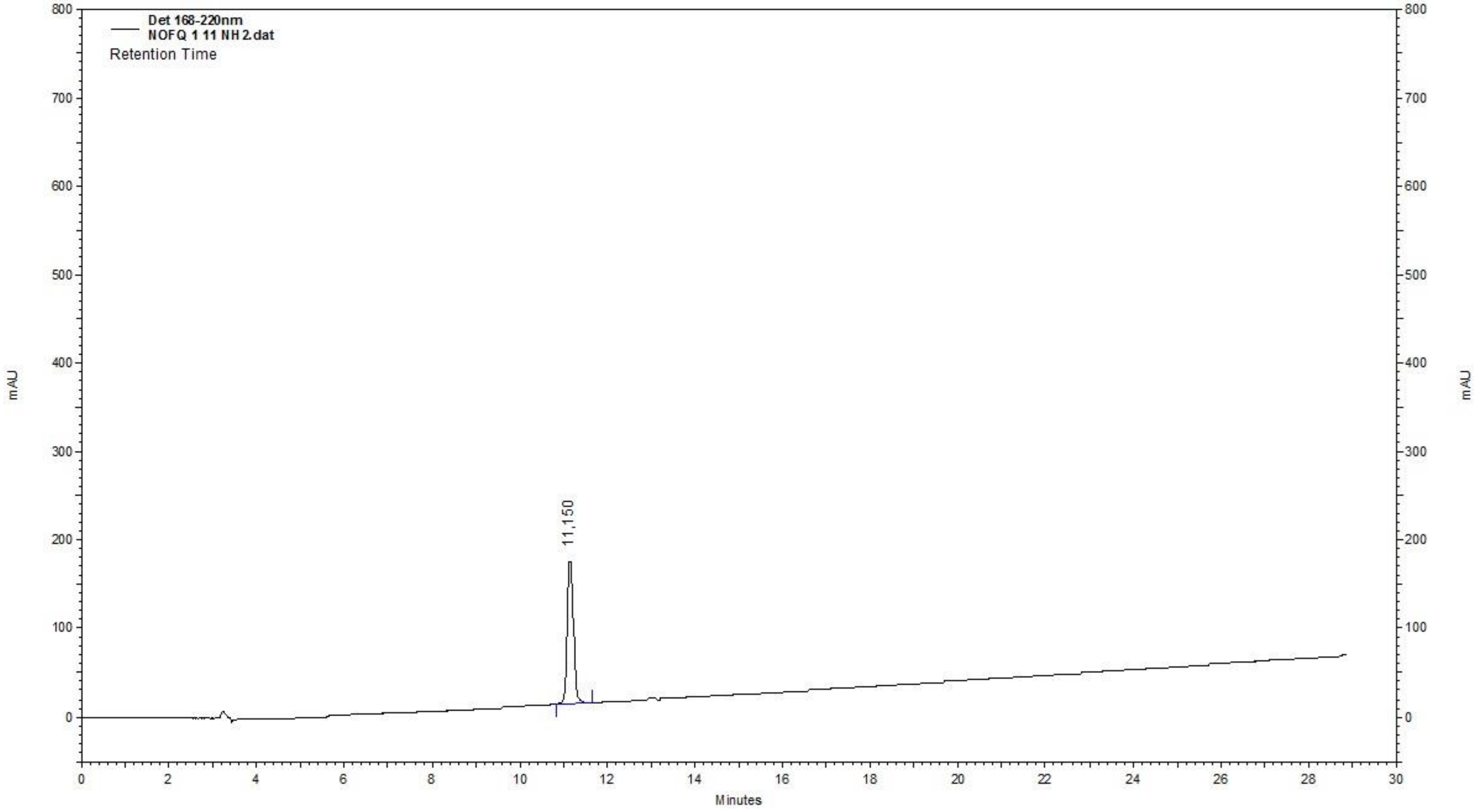

Det 168-220nm
NOFQ 1 11 NH 2.dat
Retention Time
11,150
mAU
Minutes

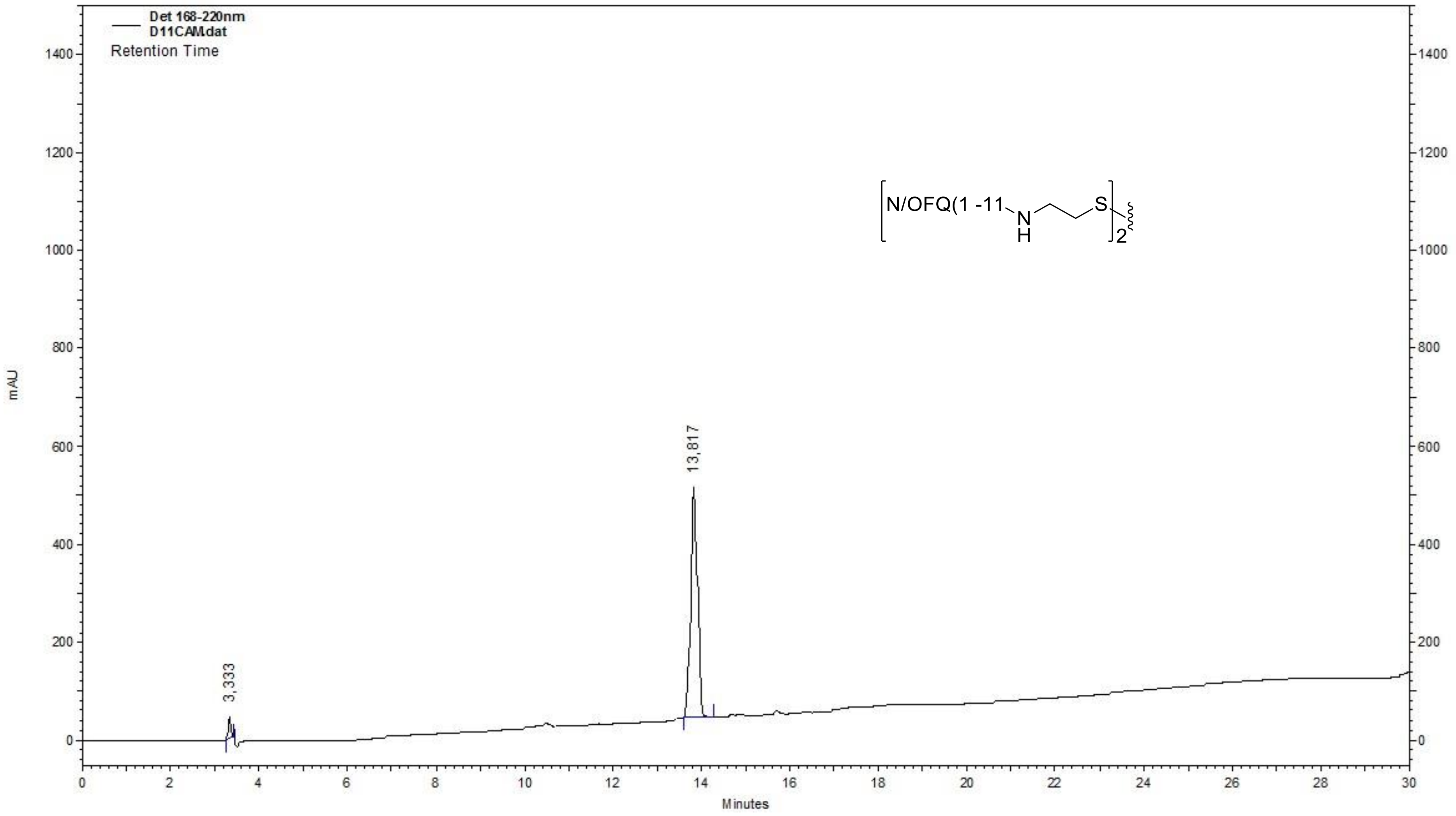

Det 168-220nm
D11CAM.dat
Retention Time
1400
1200
1000
800
600
400
200
0
mAU
3,333
13,817
N/OFQ(1 -11
N
H
S
2
0
2
4
6
8
10
12
14
16
18
20
22
24
26
28
30
Minutes

N/OFQ(2-12)-NH_2

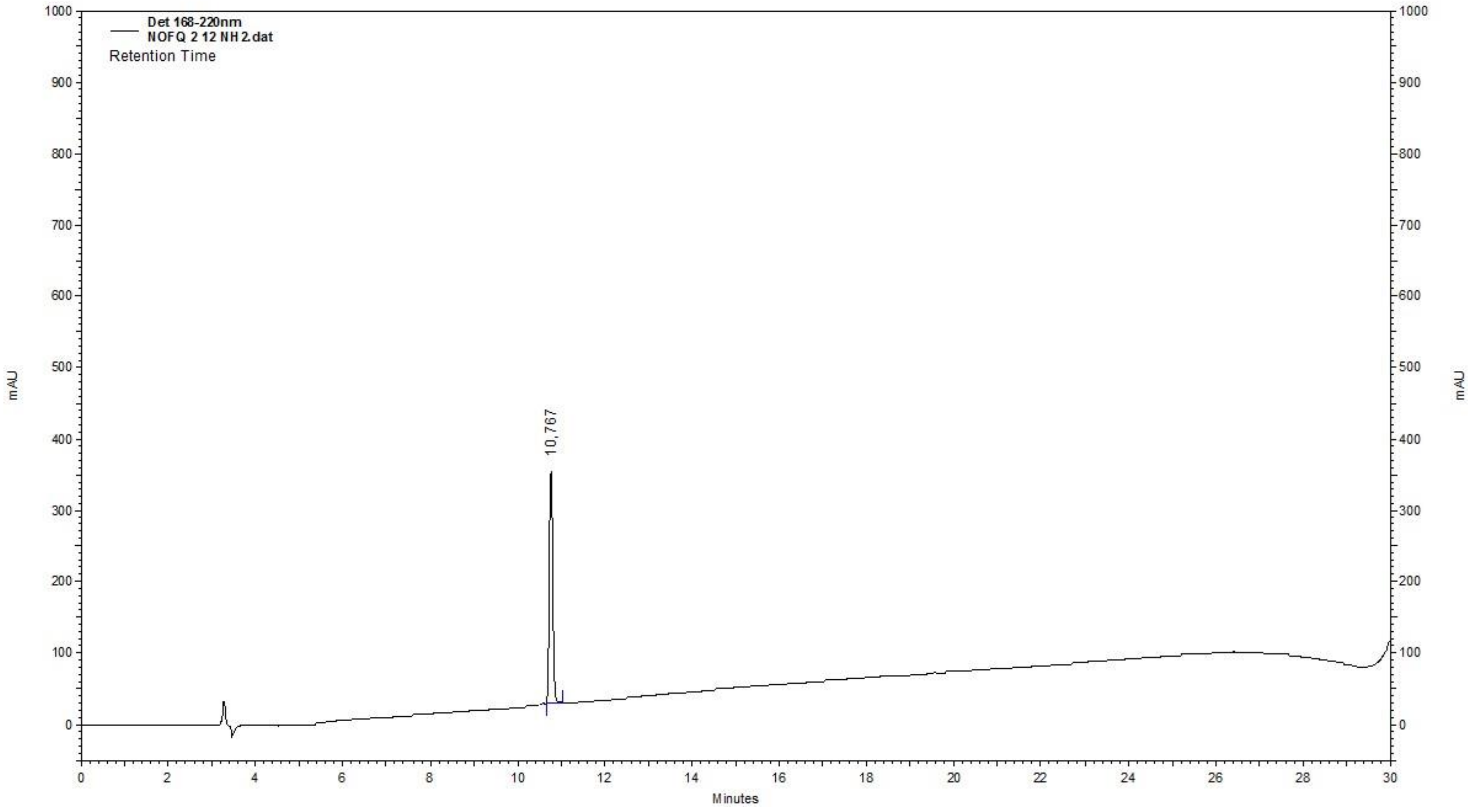

Det 168-220nm
NOFQ 2 12 NH 2.dat
Retention Time
10,767
mAU
mAU
Minutes

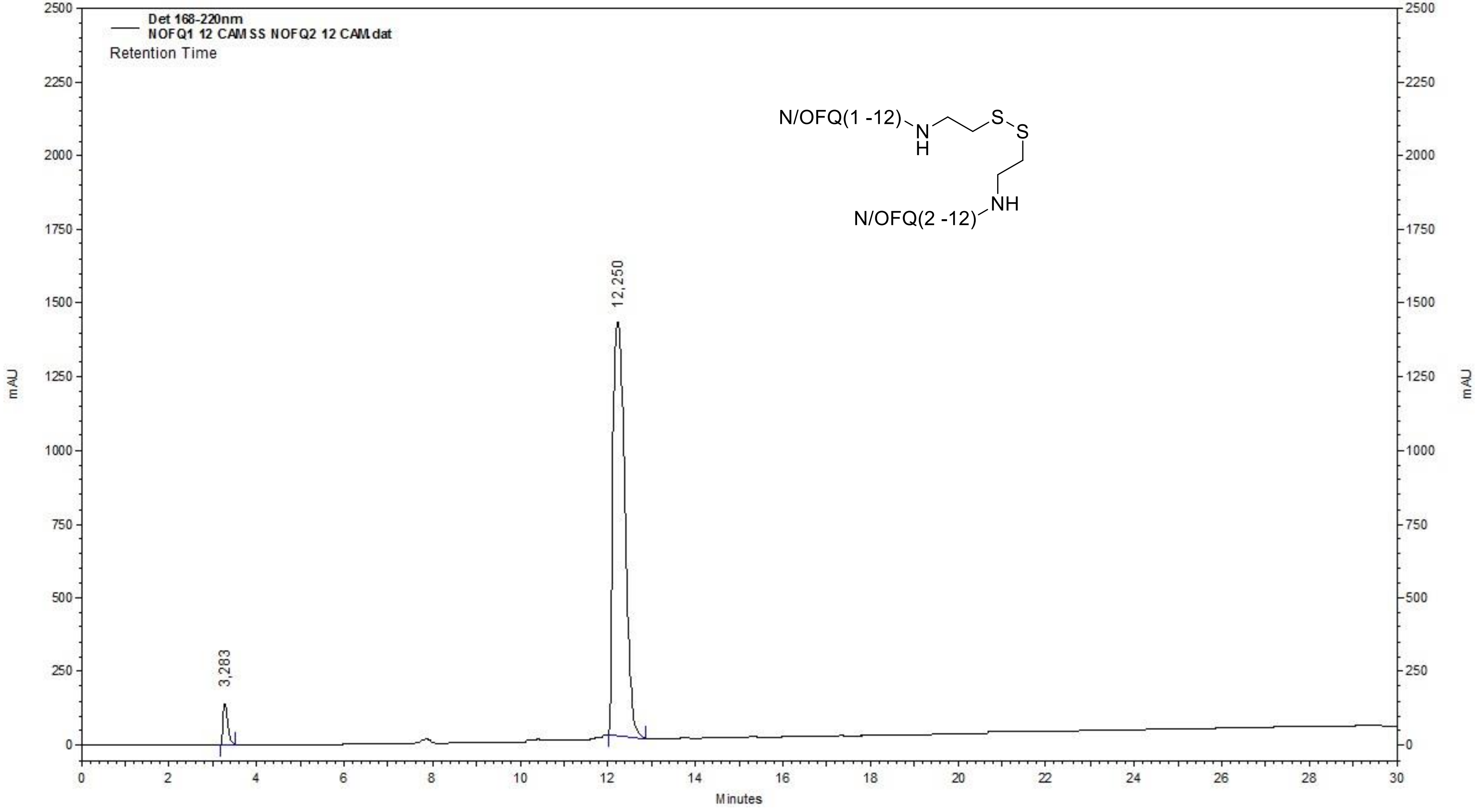
Det 168-220nm
NOFQ1 12 CAM SS NOFQ2 12 CAM.dat
Retention Time
N/OFQ(1 -12)
N
H
S
S
NH
N/OFQ(2 -12)
12,250
3,283
mAU
Minutes

Ro 65-6570

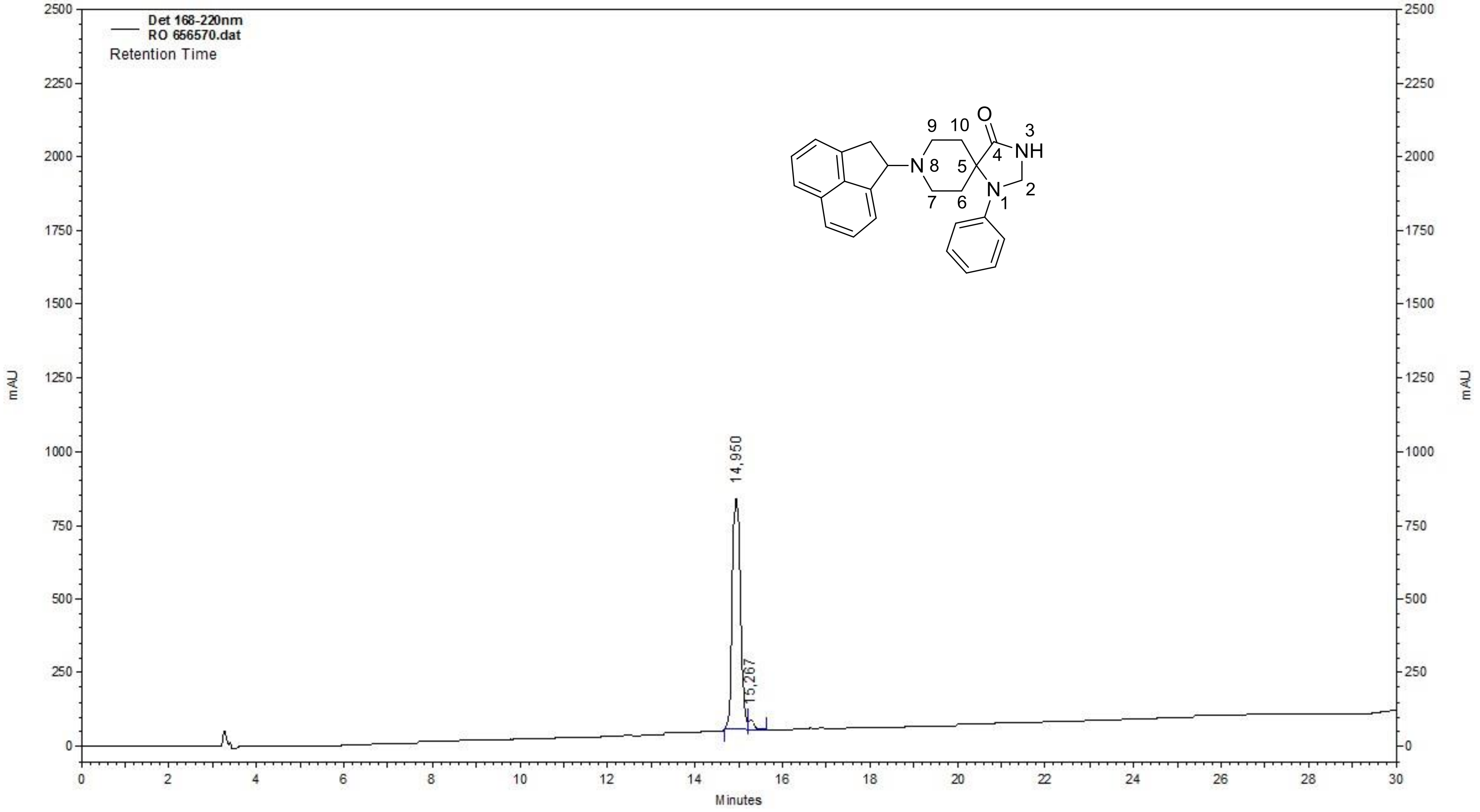

Det 168-220nm
RO 656570.dat
Retention Time
14,950
15,267
mAU
Minutes
O
NH
N
N
1
2
3
4
5
6
7
8
9
10

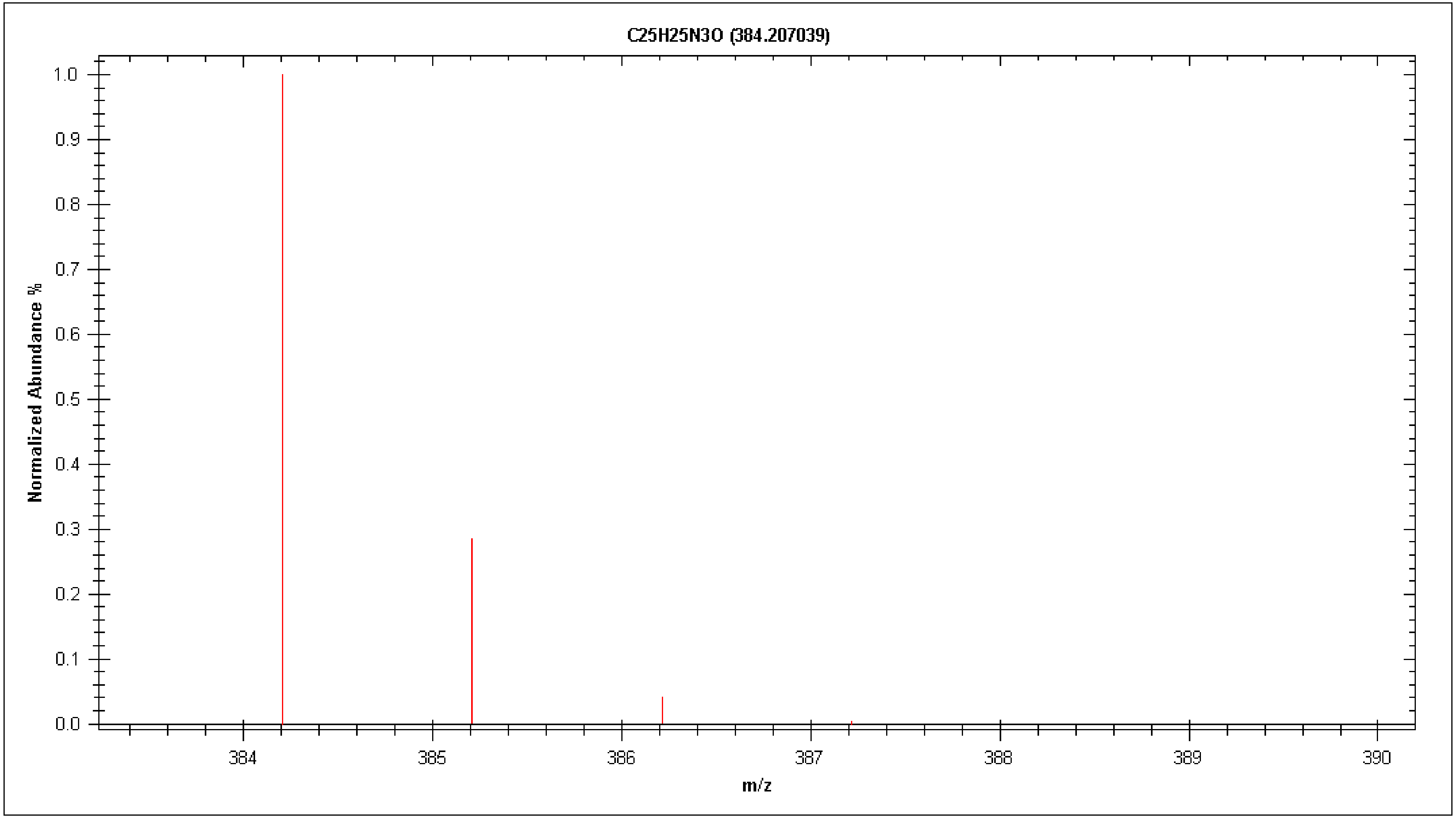
C25H25N3O (384.207039)
1.0
0.9
0.8
0.7
0.6
0.5
0.4
0.3
0.2
0.1
0.0
Normalized Abundance %
384
385
386
387
388
389
390
m/z

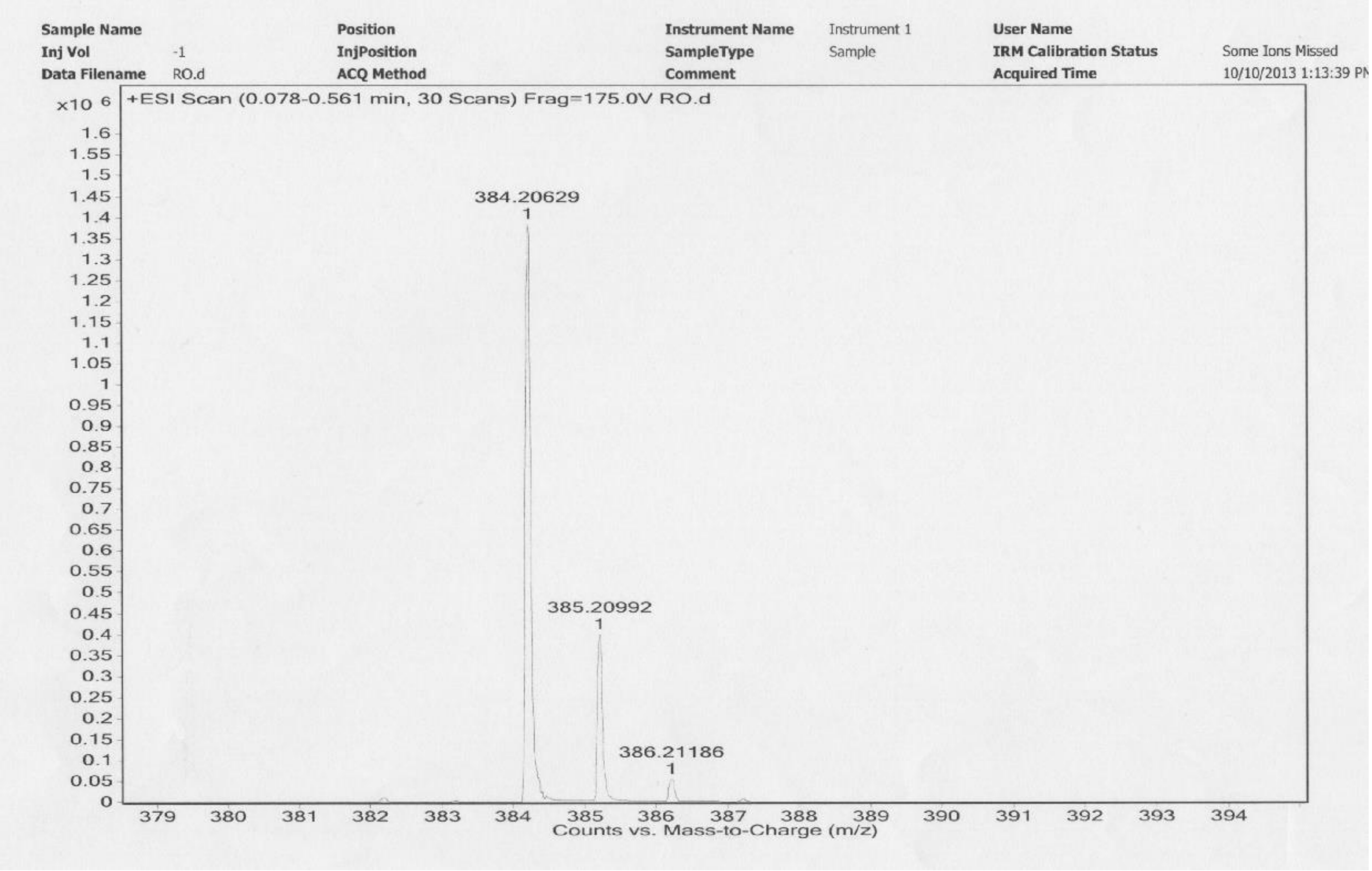
Sample Name
Inj Vol -1
Data Filename RO.d
Position
InjPosition
ACQ Method
Instrument Name Instrument 1
SampleType Sample
Comment
User Name
IRM Calibration Status Some Ions Missed
Acquired Time 10/10/2013 1:13:39 PM
x10 6
+ESI Scan (0.078-0.561 min, 30 Scans) Frag=175.0V RO.d
384.20629
1
385.20992
1
386.21186
1
Counts vs. Mass-to-Charge (m/z)

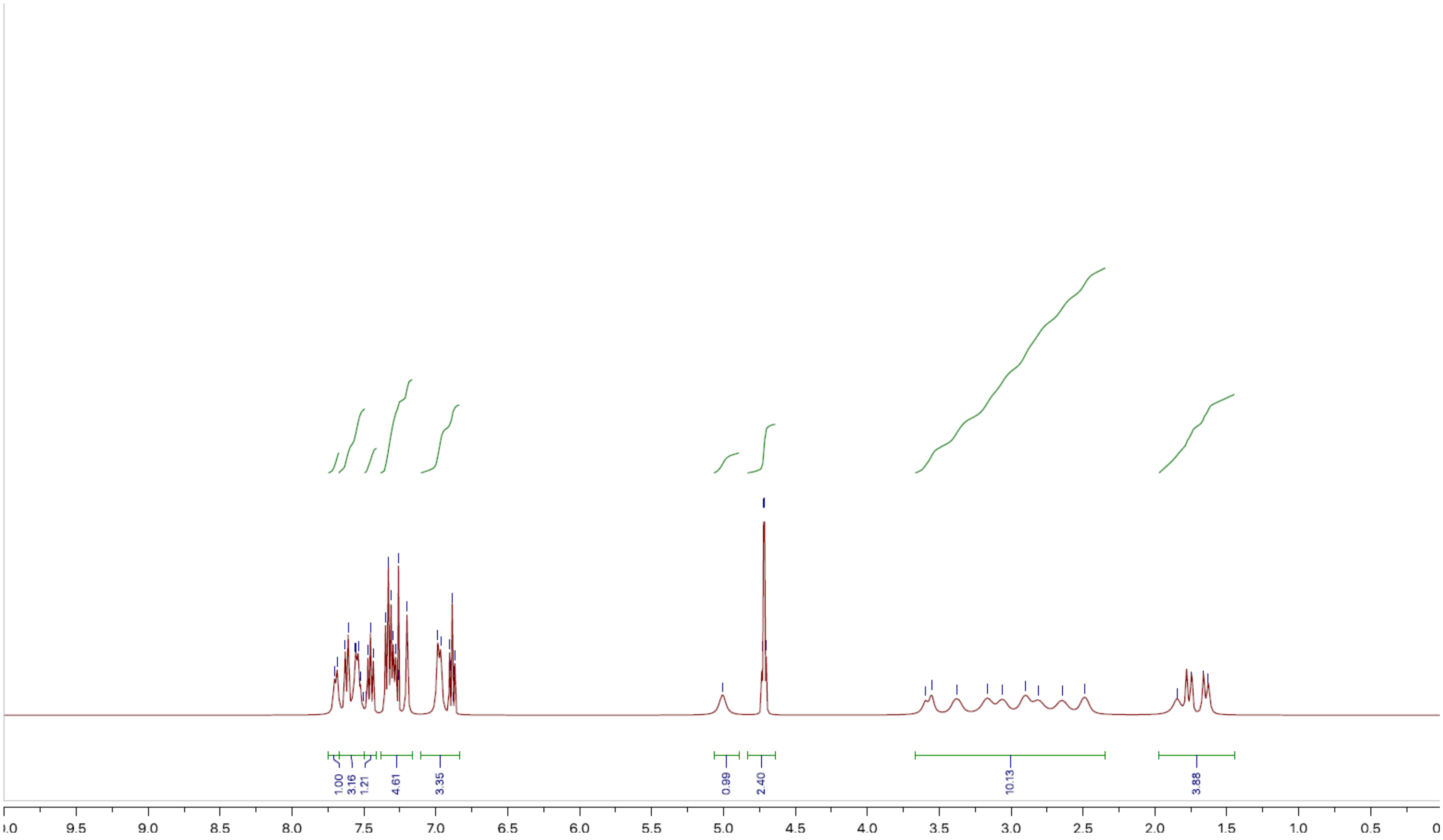
1.00
3.16
1.21
4.61
3.35
0.99
2.40
10.13
3.88
9.5
9.0
8.5
8.0
7.5
7.0
6.5
6.0
5.5
5.0
4.5
4.0
3.5
3.0
2.5
2.0
1.5
1.0
0.5

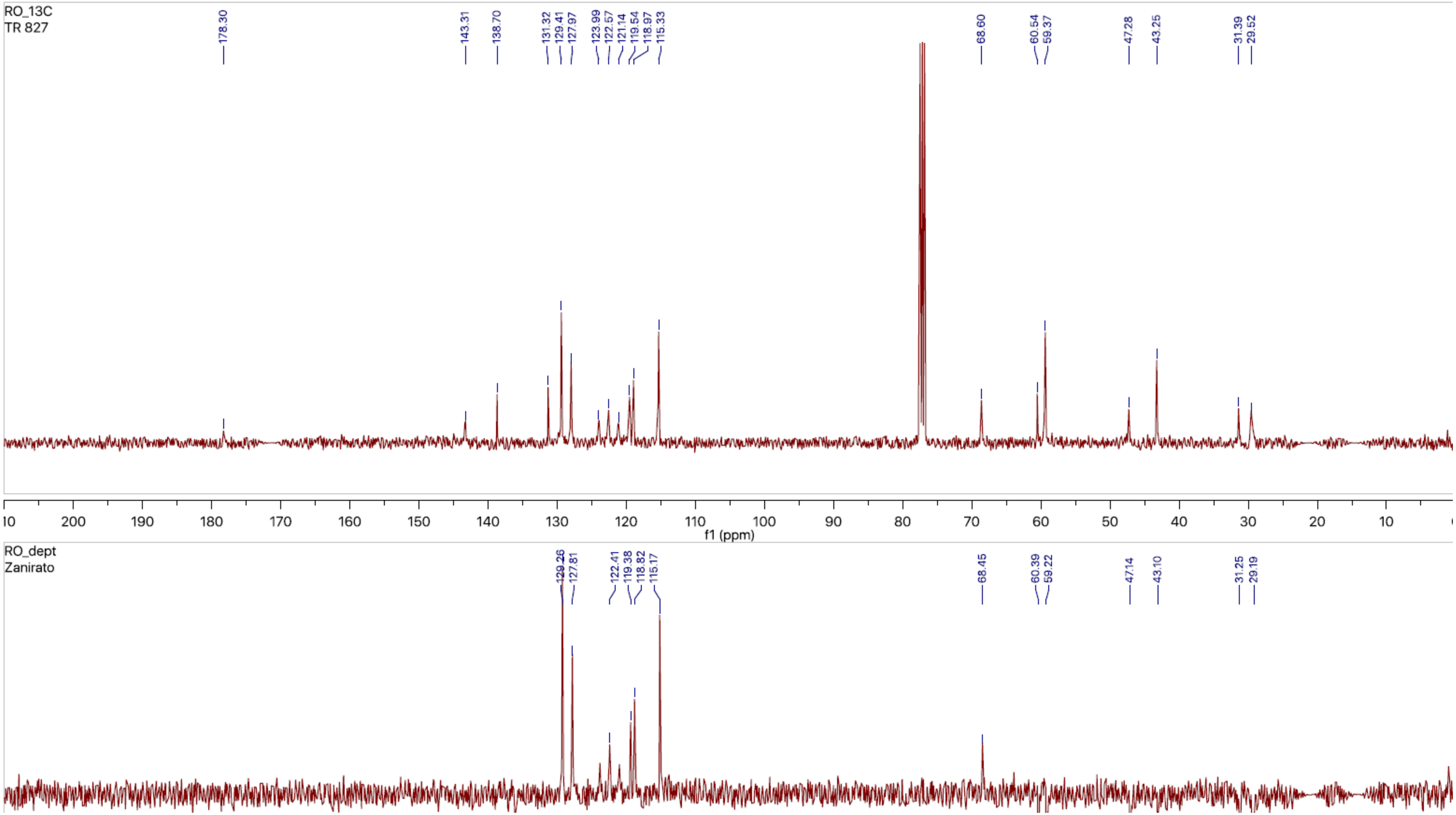
RO_13C
TR 827
178.30
143.31
138.70
131.32
129.41
127.97
123.99
122.57
121.14
119.54
118.97
115.33
68.60
60.54
59.37
47.28
43.25
31.39
29.52
200
190
180
170
160
150
140
130
120
110
100
90
80
70
60
50
40
30
20
10
f1 (ppm)
RO_dept
Zanirato
129.26
127.81
122.41
119.38
118.82
115.17
68.45
60.39
59.22
47.14
43.10
31.25
29.19

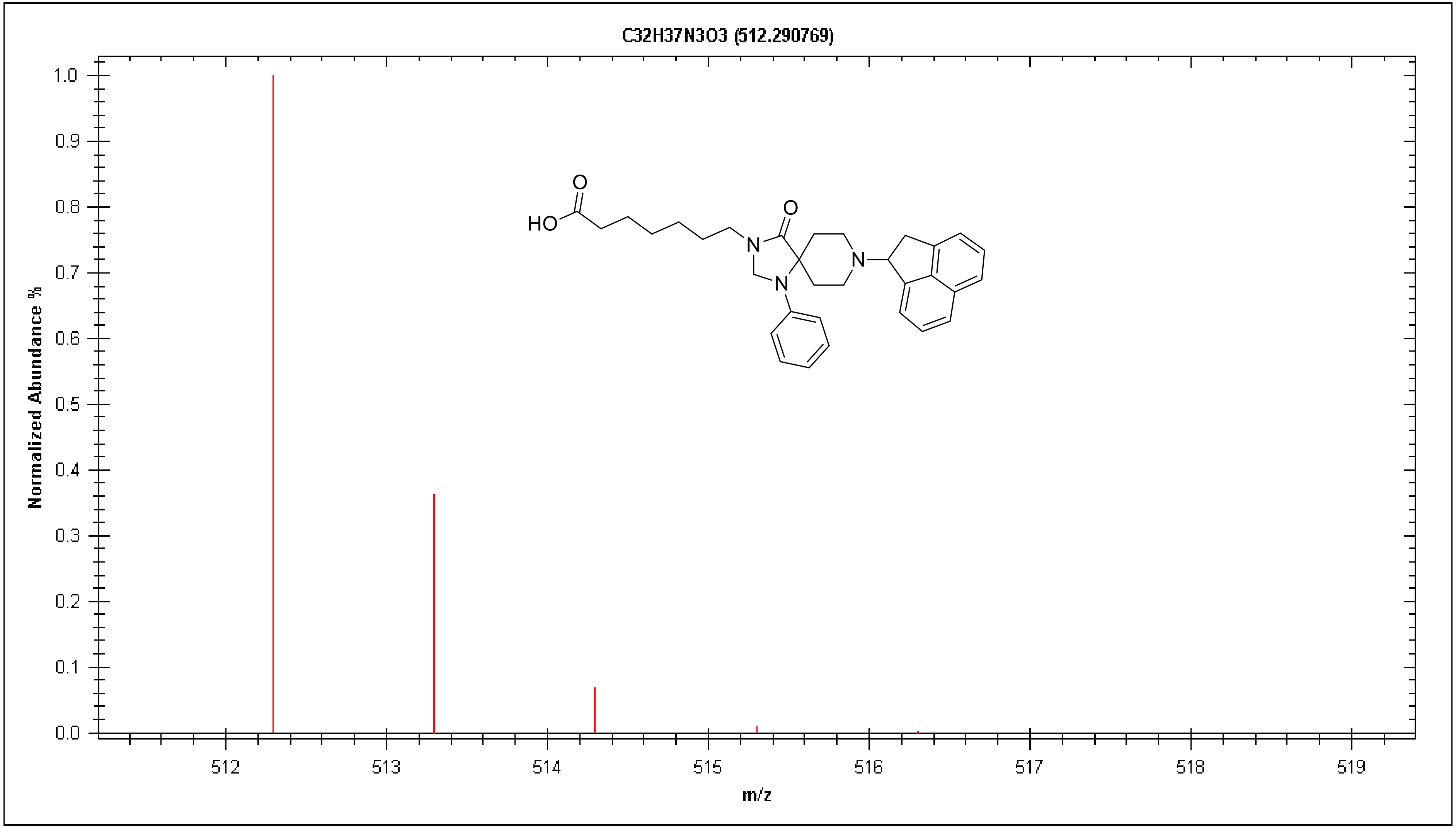

C32H37N3O3 (512.290769)
Normalized Abundance %
1.0
0.9
0.8
0.7
0.6
0.5
0.4
0.3
0.2
0.1
0.0
512
513
514
515
516
517
518
519
m/z
HO
O
O
N
N
N

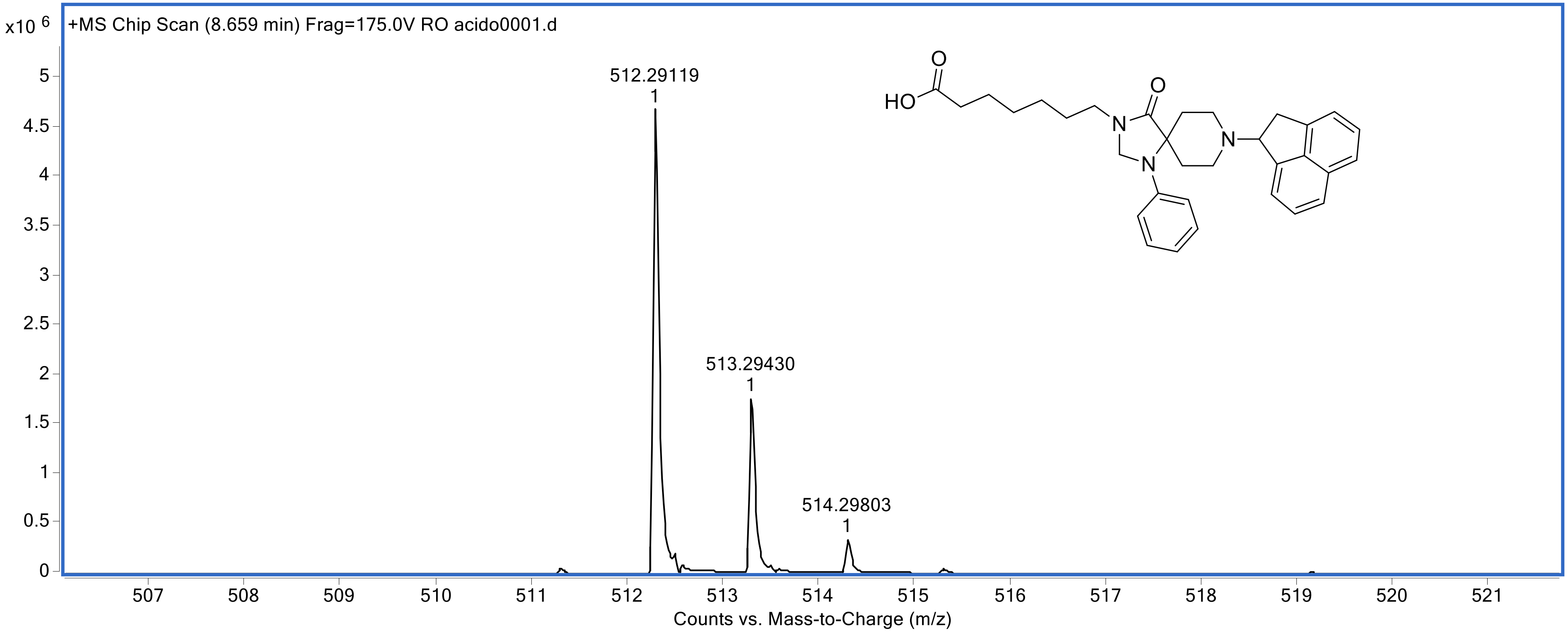

Calc. Mass	Mis. Mass	Error ppm
512.29077	512.29111	0.665638

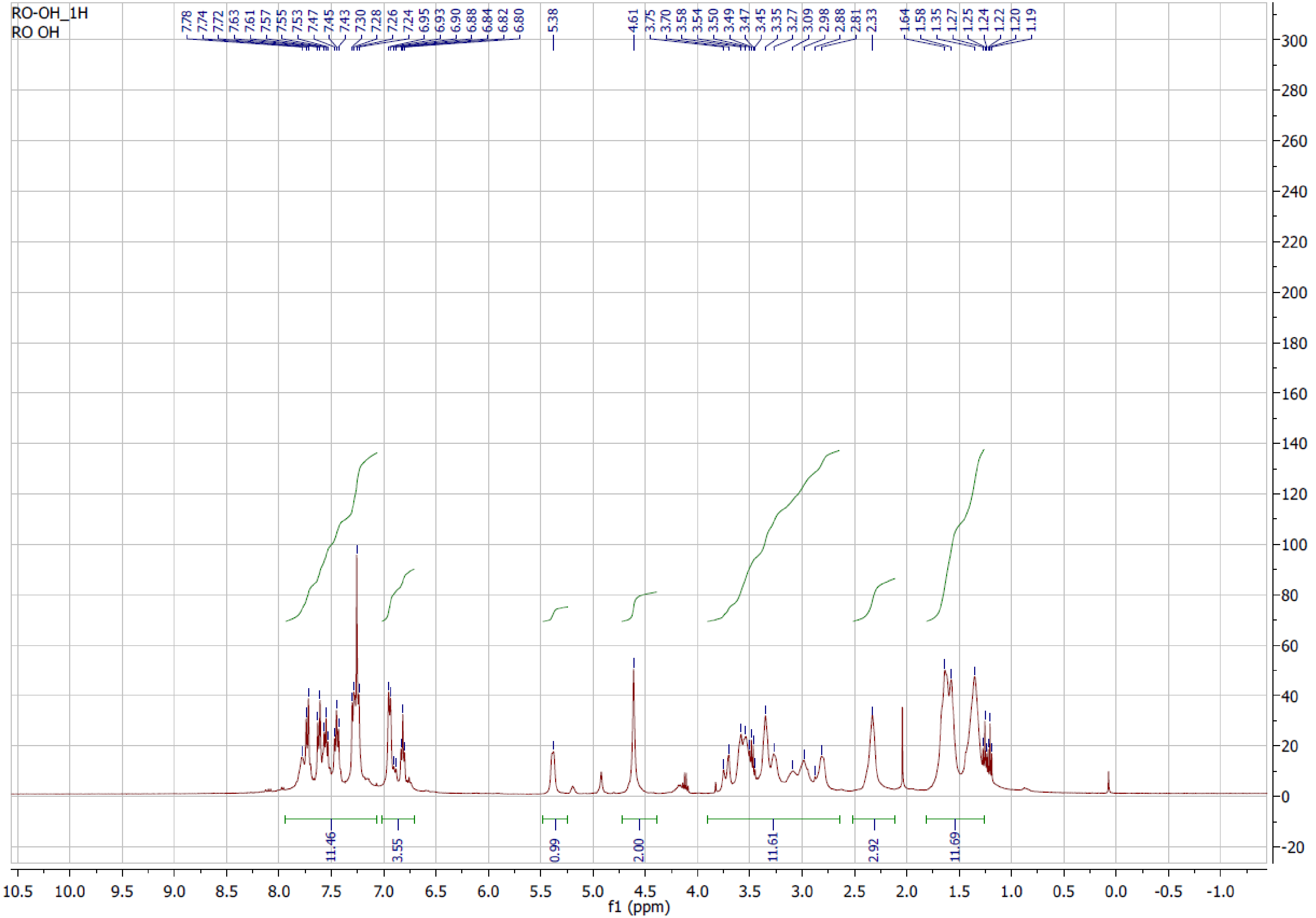
RO-OH_1H
RO OH
7.78 7.74 7.72 7.63 7.61 7.57 7.55 7.53 7.47 7.45 7.43 7.30 7.28 7.26 7.24 6.95 6.93 6.90 6.88 6.84 6.82 6.80
5.38
4.61 3.75 3.70 3.58 3.54 3.50 3.49 3.47 3.45 3.35 3.27 3.09 2.98 2.88 2.81 2.33
1.64 1.58 1.35 1.27 1.25 1.24 1.22 1.20 1.19
11.46
3.55
0.99
2.00
11.61
2.92
11.69
f1 (ppm)

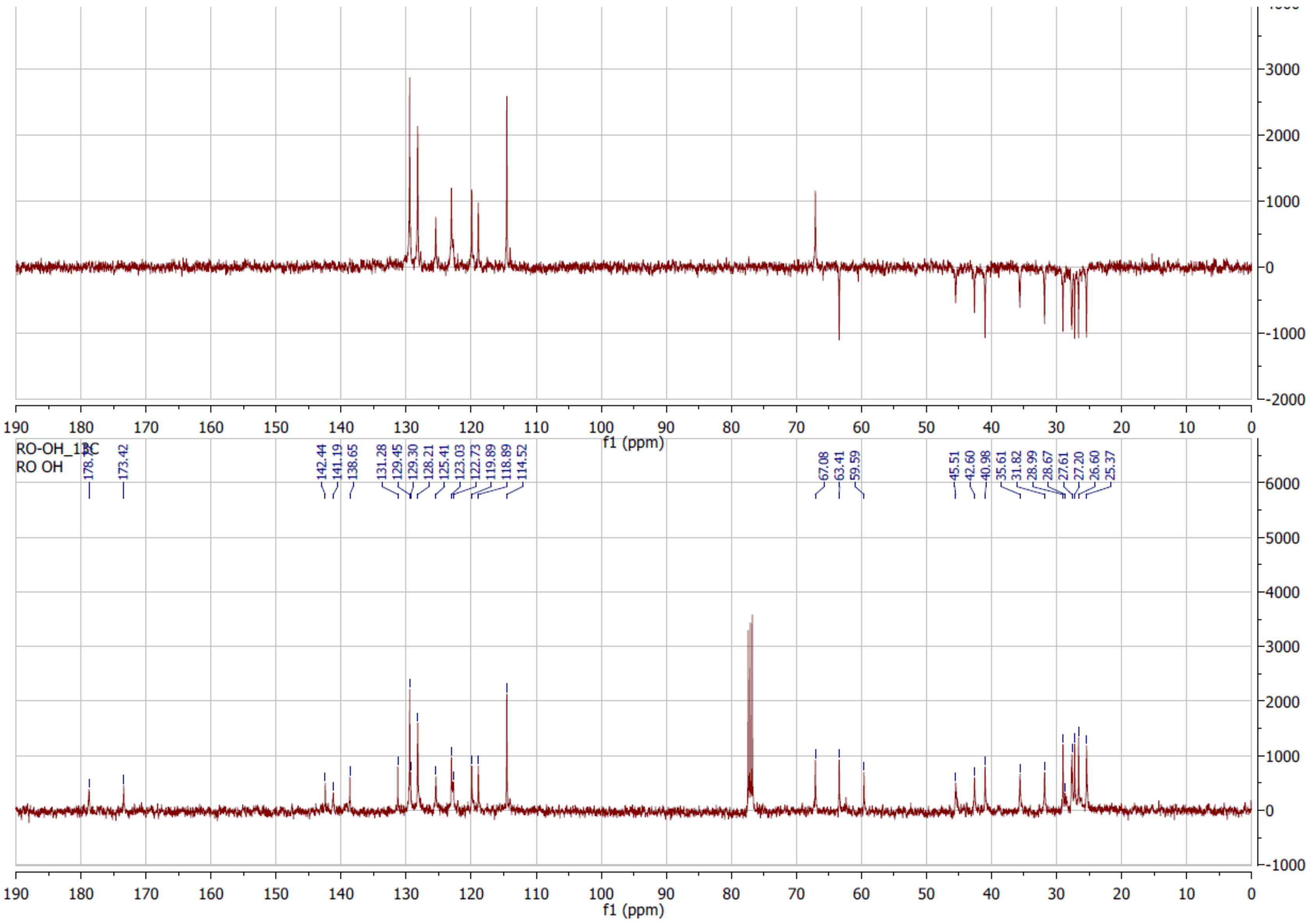
RO-OH_13C
RO OH
178.
173.42
142.44
141.19
138.65
131.28
129.45
129.30
128.21
125.41
123.03
122.73
119.89
118.89
114.52
67.08
63.41
59.59
45.51
42.60
40.98
35.61
31.82
28.99
28.67
27.61
27.20
26.60
25.37
f1 (ppm)
f1 (ppm)

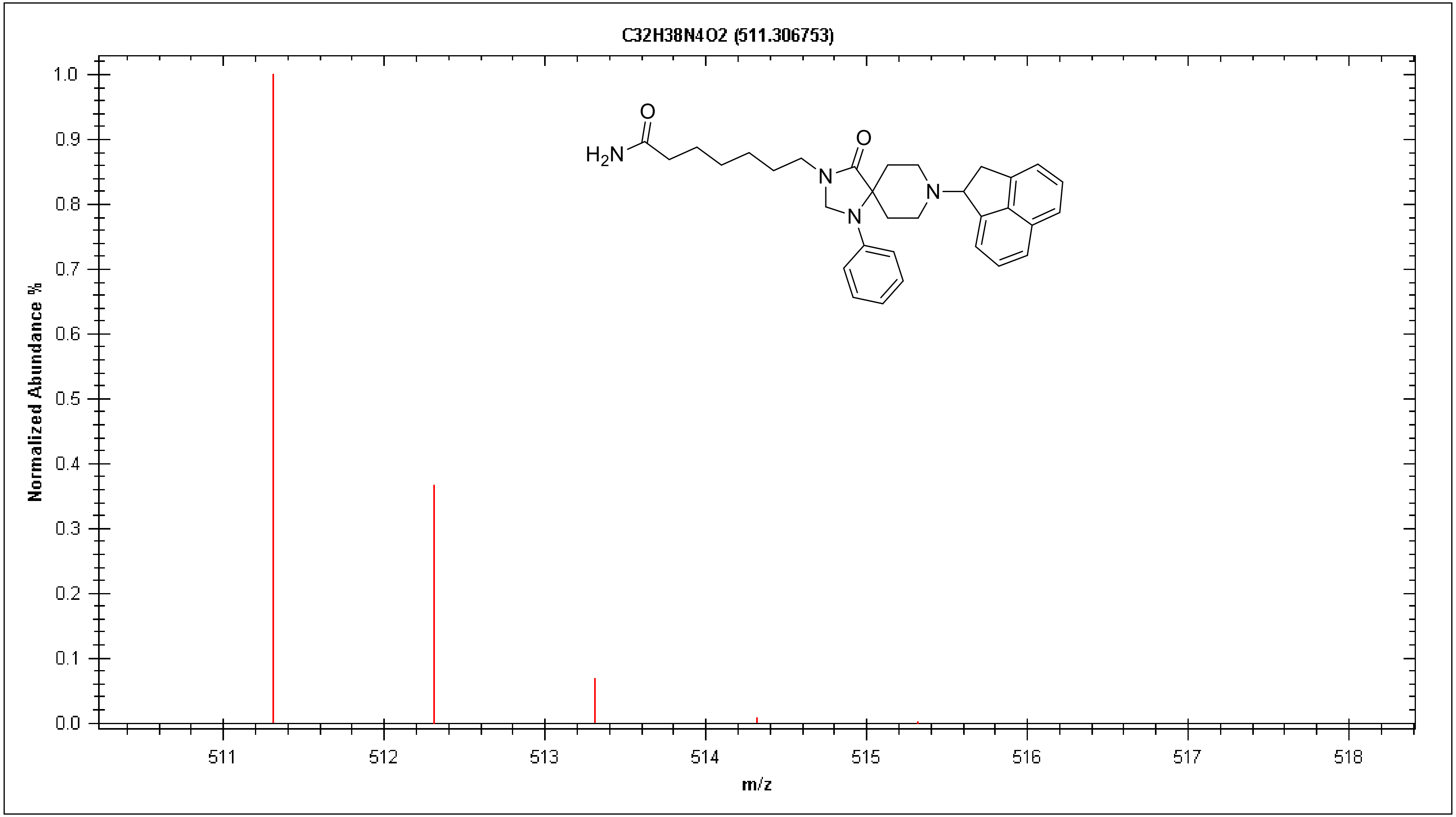
C32H38N4O2 (511.306753)
Normalized Abundance %
m/z
H2N
O
N
N
N
1.0
0.9
0.8
0.7
0.6
0.5
0.4
0.3
0.2
0.1
0.0
511
512
513
514
515
516
517
518

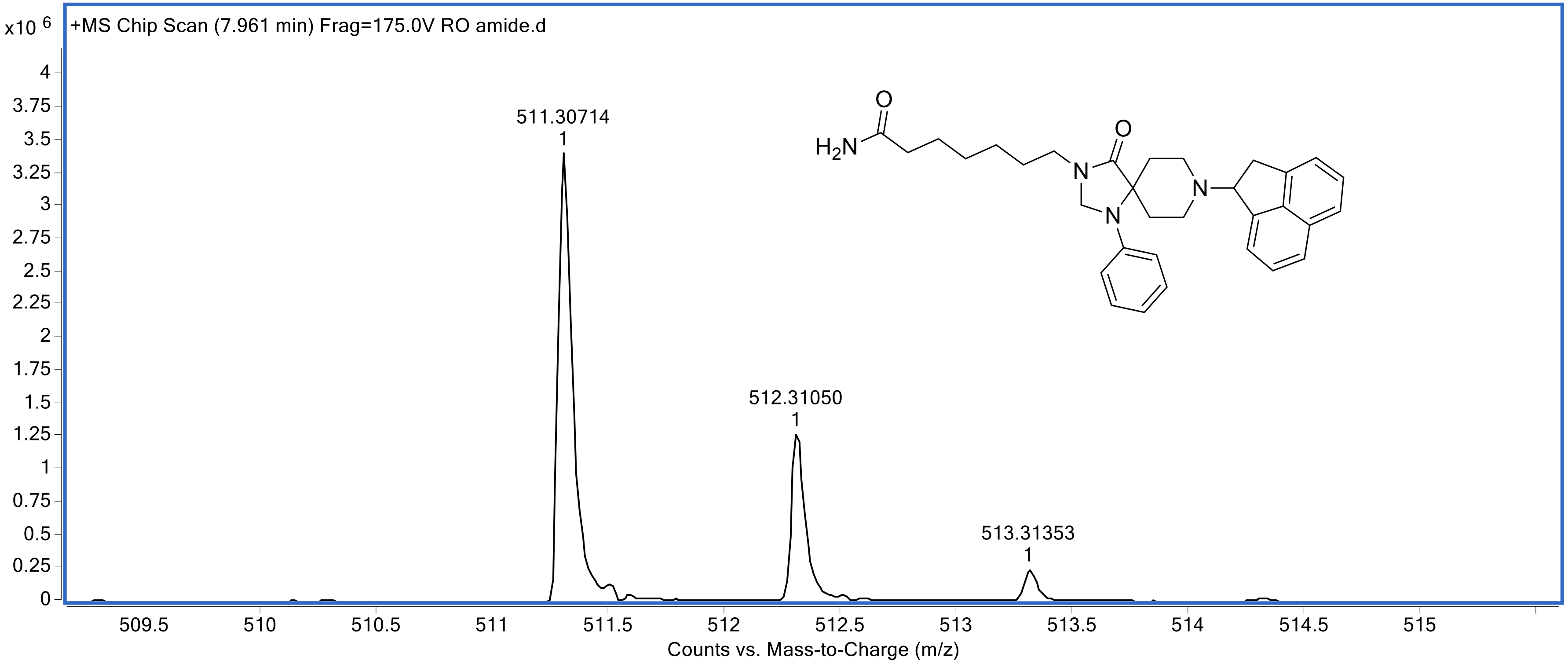

Calc. Mass	Mis. Mass	Error ppm
511.306753	511.30714	0.756884

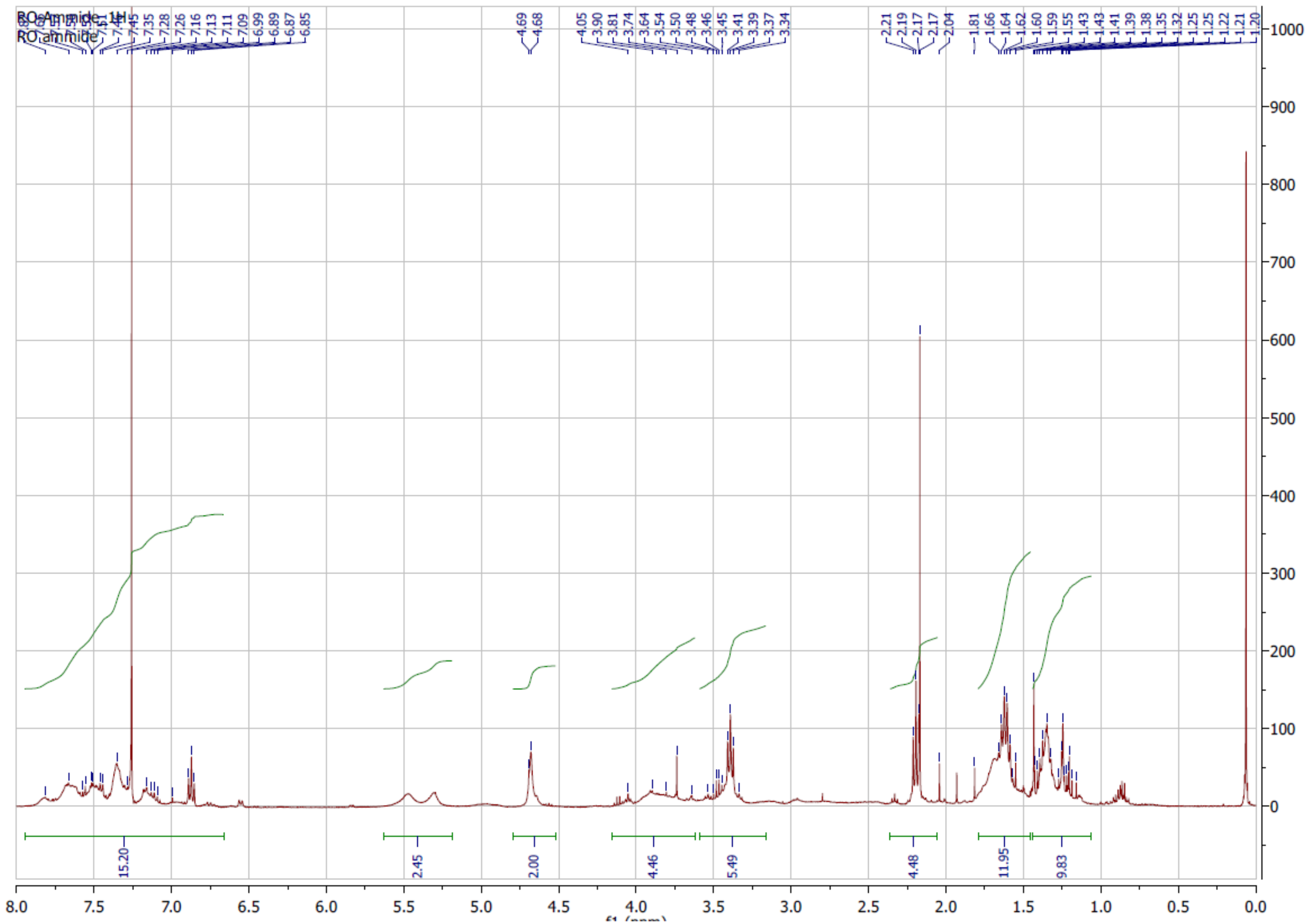

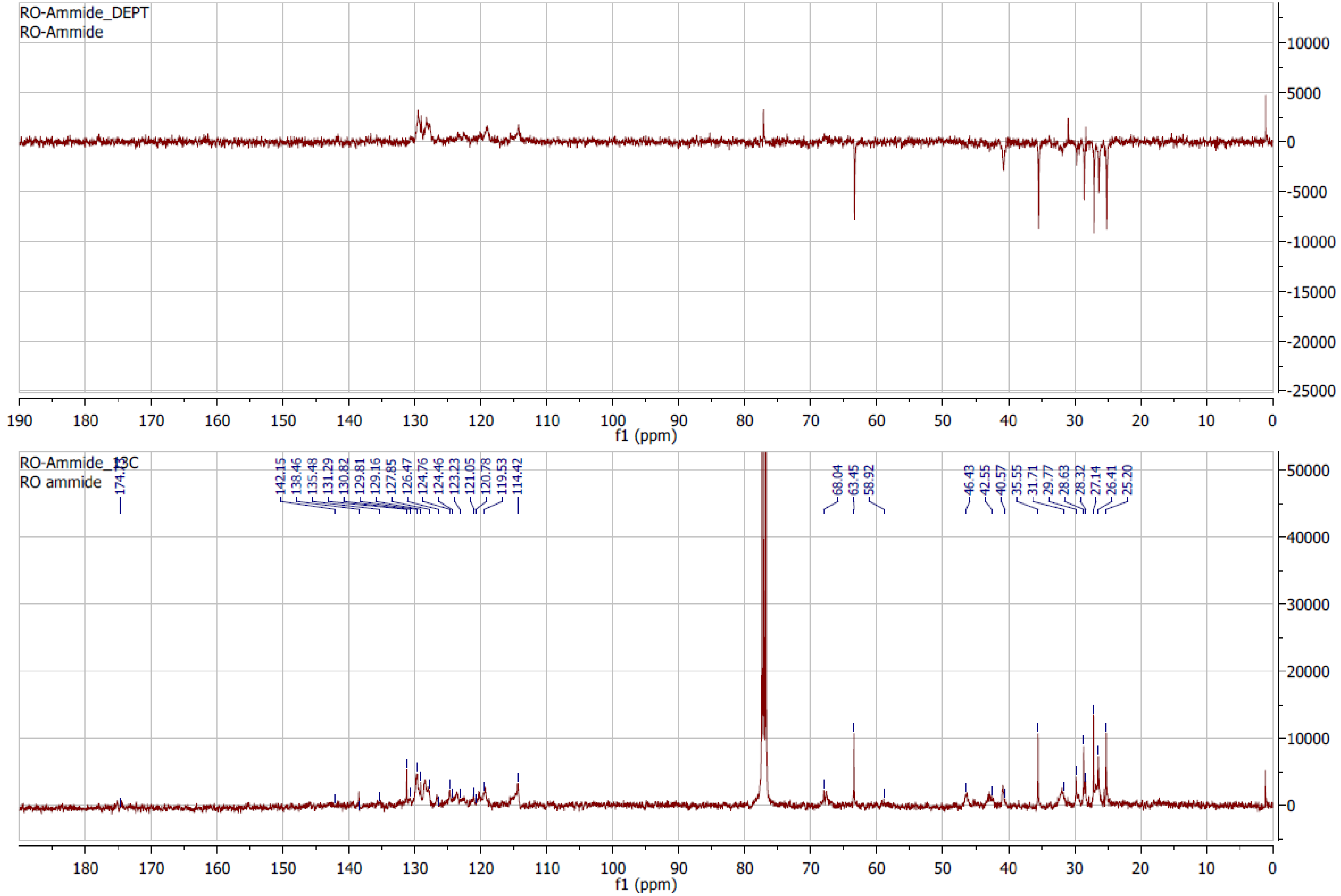
RO-Ammide_DEPT
RO-Ammide
f1 (ppm)
RO-Ammide_13C
RO ammide
142.15
138.46
135.48
131.29
130.82
129.81
129.16
127.85
126.47
124.76
124.46
123.23
121.05
120.78
119.53
114.42
68.04
63.45
58.92
46.43
42.55
40.57
35.55
31.71
29.77
28.63
28.32
27.14
26.41
25.20
f1 (ppm)

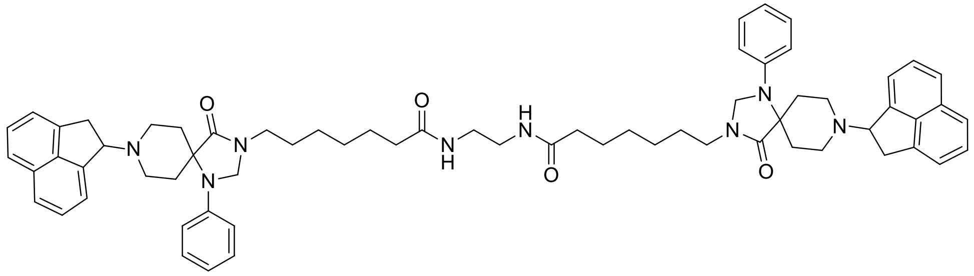

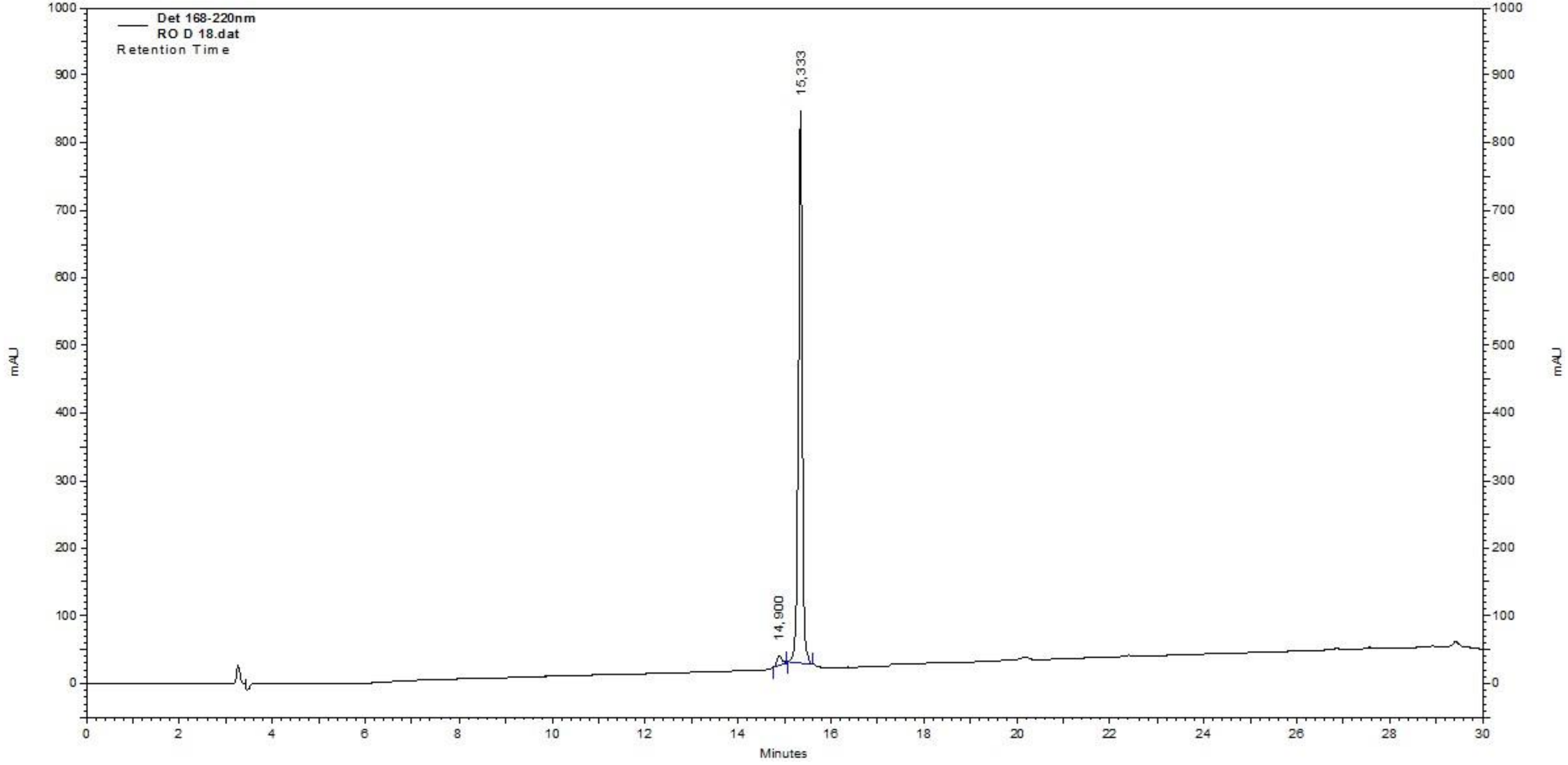

Det 168-220nm
RO D 18.dat
Retention Time
15,333
14,900
mAU
mAU
Minutes

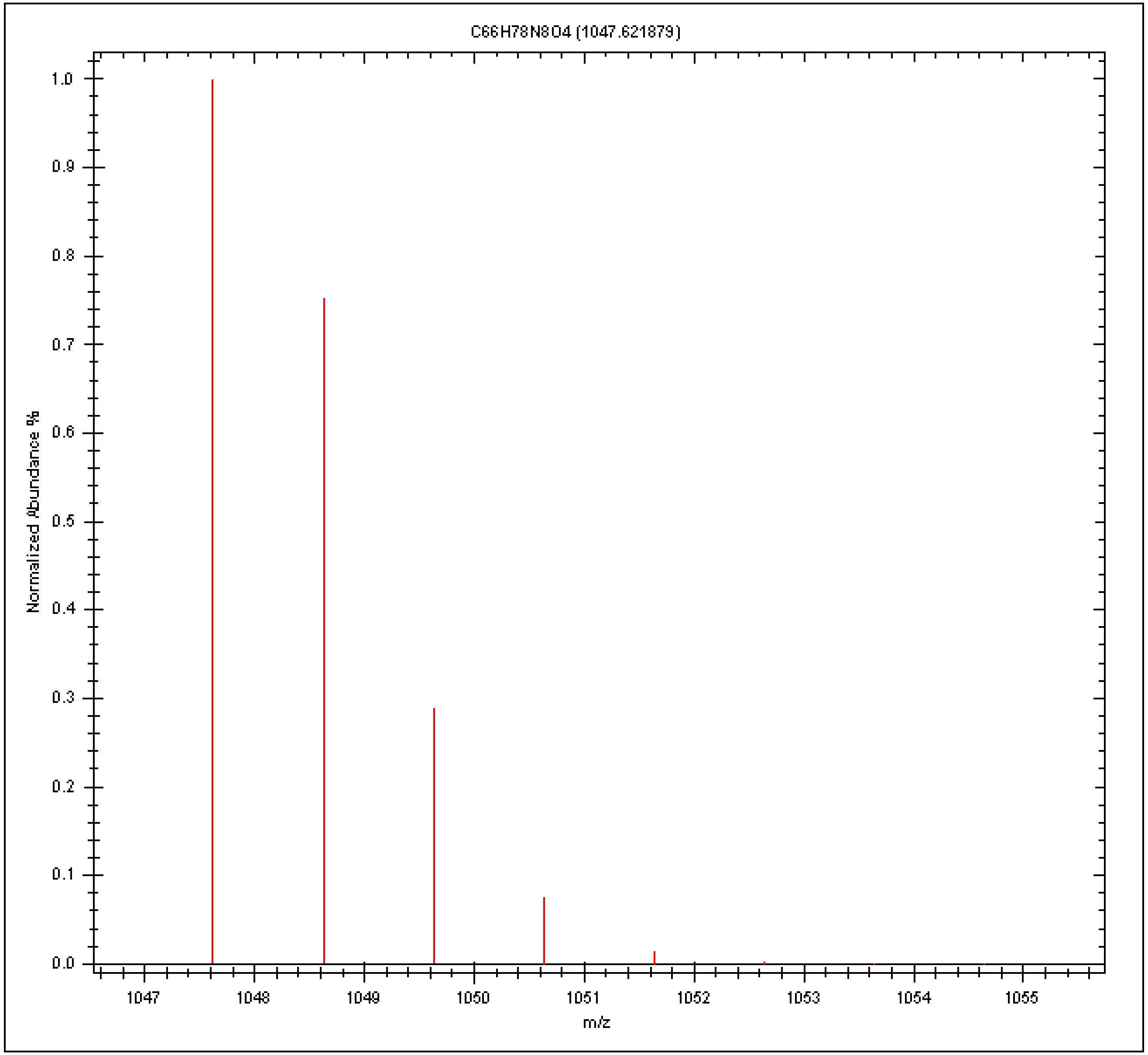

C66H78N8O4 (1047.621879)
Normalized Abundance %
1.0
0.9
0.8
0.7
0.6
0.5
0.4
0.3
0.2
0.1
0.0
1047
1048
1049
1050
1051
1052
1053
1054
1055
m/z

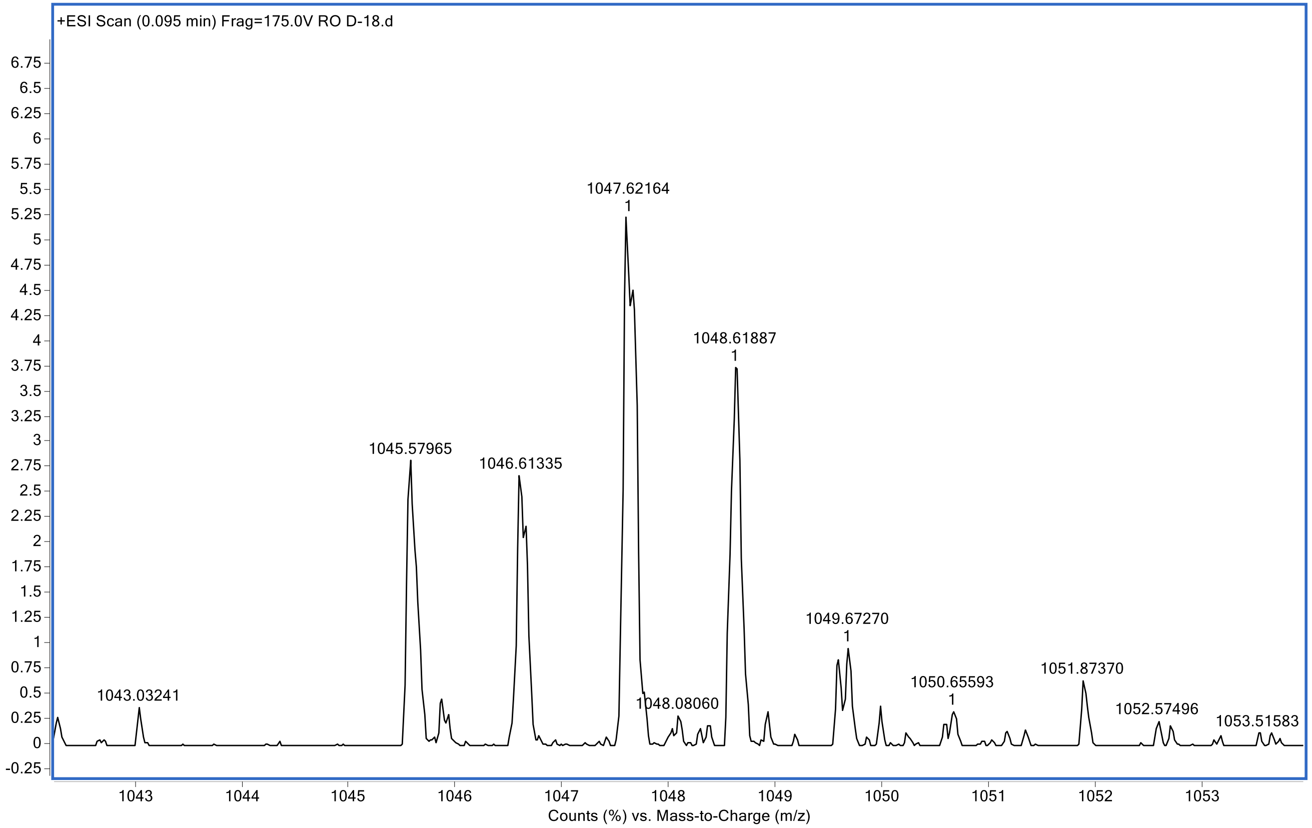
+ESI Scan (0.095 min) Frag=175.0V RO D-18.d
1043.03241
1045.57965
1046.61335
1047.62164
1
1048.08060
1048.61887
1
1049.67270
1
1050.65593
1
1051.87370
1052.57496
1053.51583
Counts (%) vs. Mass-to-Charge (m/z)

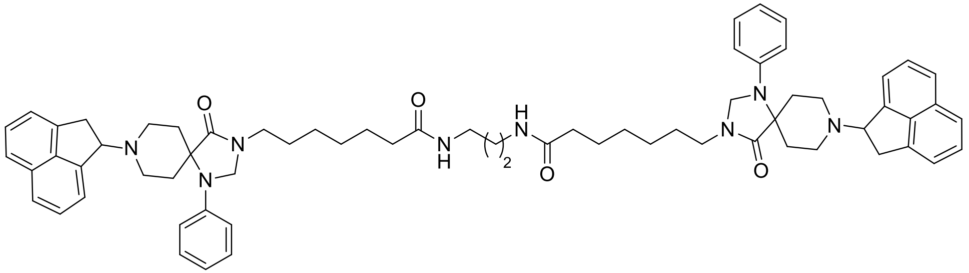
O
O
N
N
N
H
H
N
2
O
N
N
O
N
N

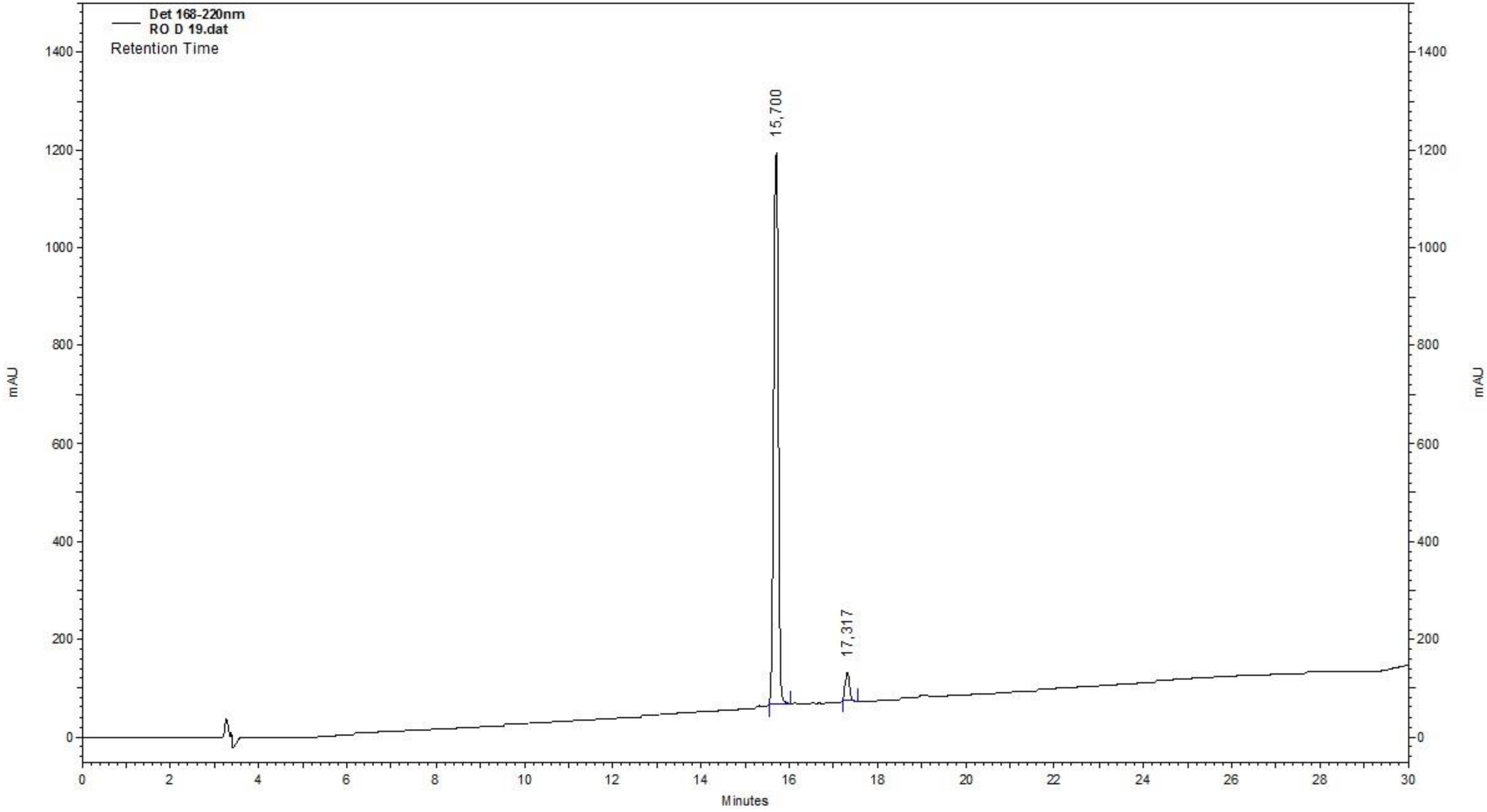
Det 168-220nm
RO D 19.dat
Retention Time
15,700
17,317
mAU
Minutes
0
2
4
6
8
10
12
14
16
18
20
22
24
26
28
30
1400
1200
1000
800
600
400
200
0

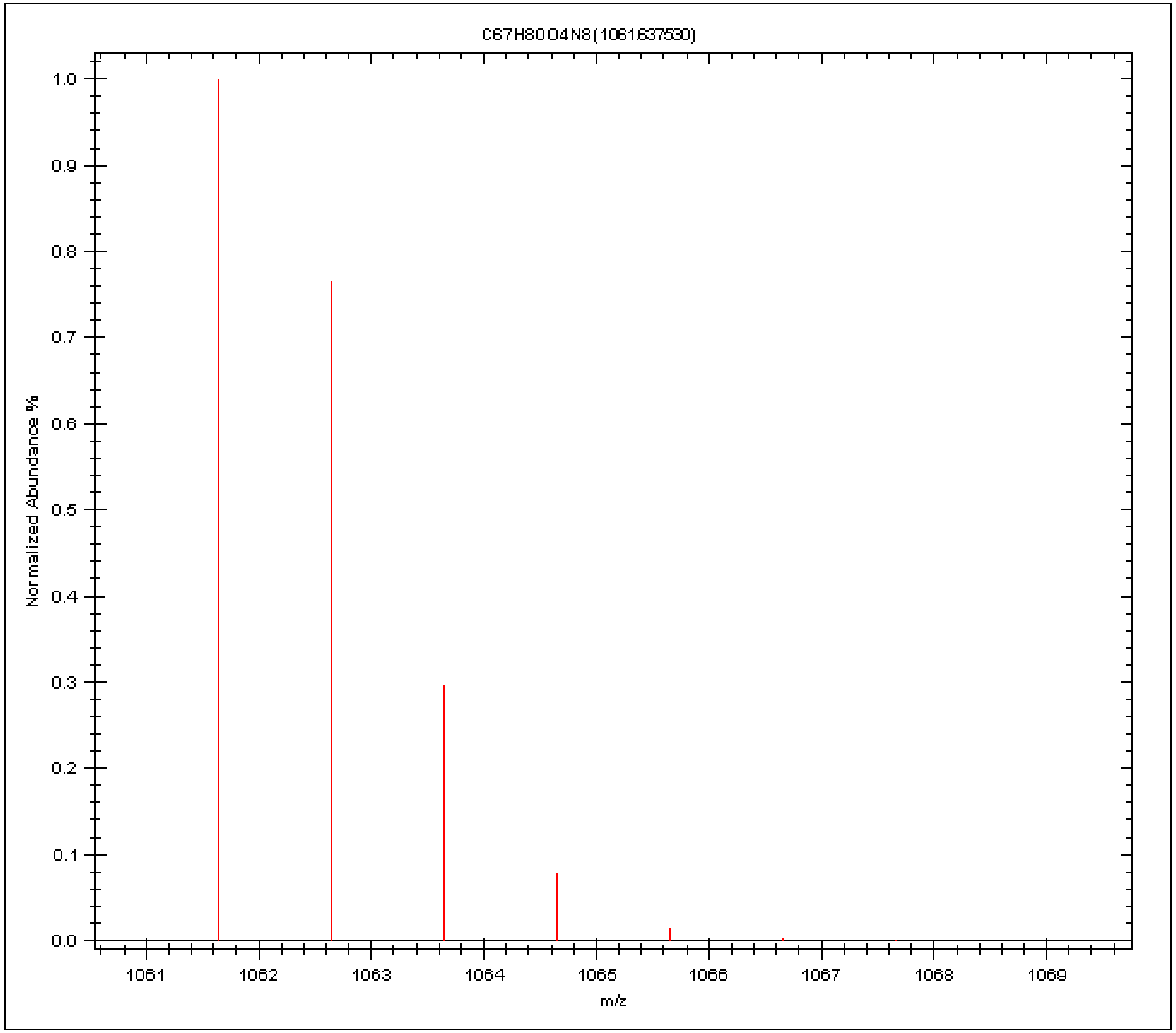
C67H80O4N8(1061.637530)
Normalized Abundance %
m/z
1.0
0.9
0.8
0.7
0.6
0.5
0.4
0.3
0.2
0.1
0.0
1061
1062
1063
1064
1065
1066
1067
1068
1069

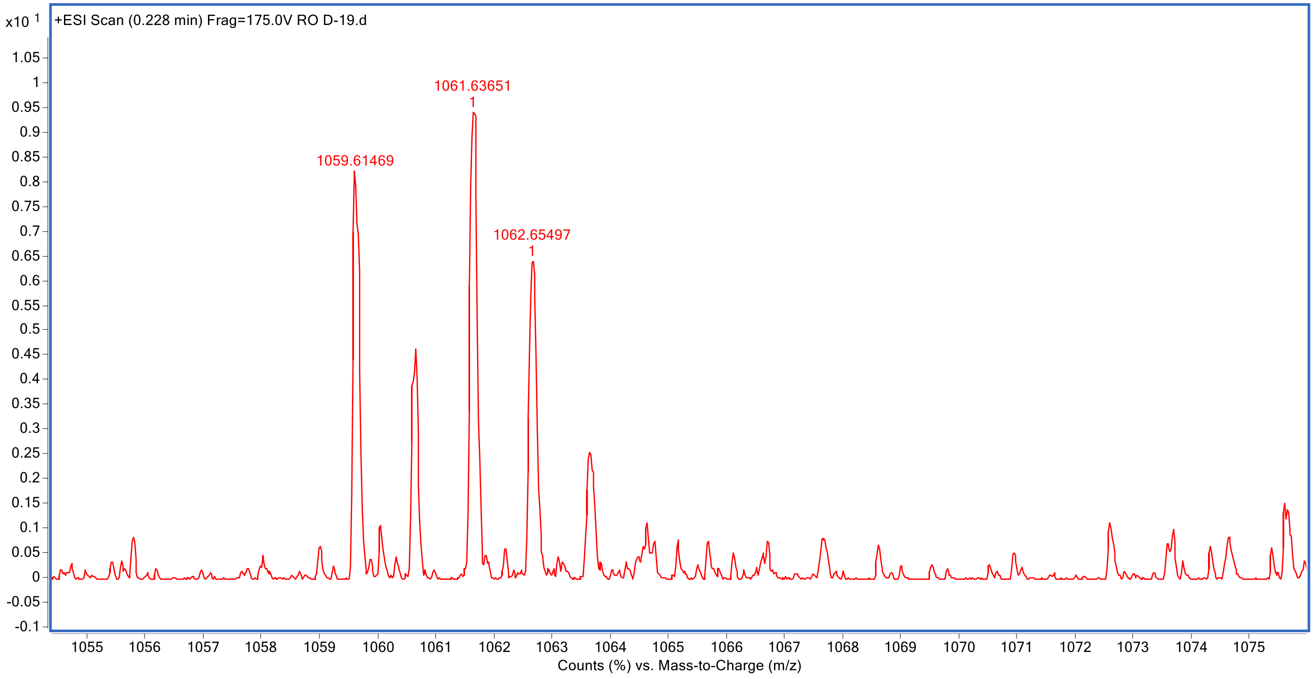

x10 1
+ESI Scan (0.228 min) Frag=175.0V RO D-19.d
1059.61469
1061.63651
1
1062.65497
1
Counts (%) vs. Mass-to-Charge (m/z)

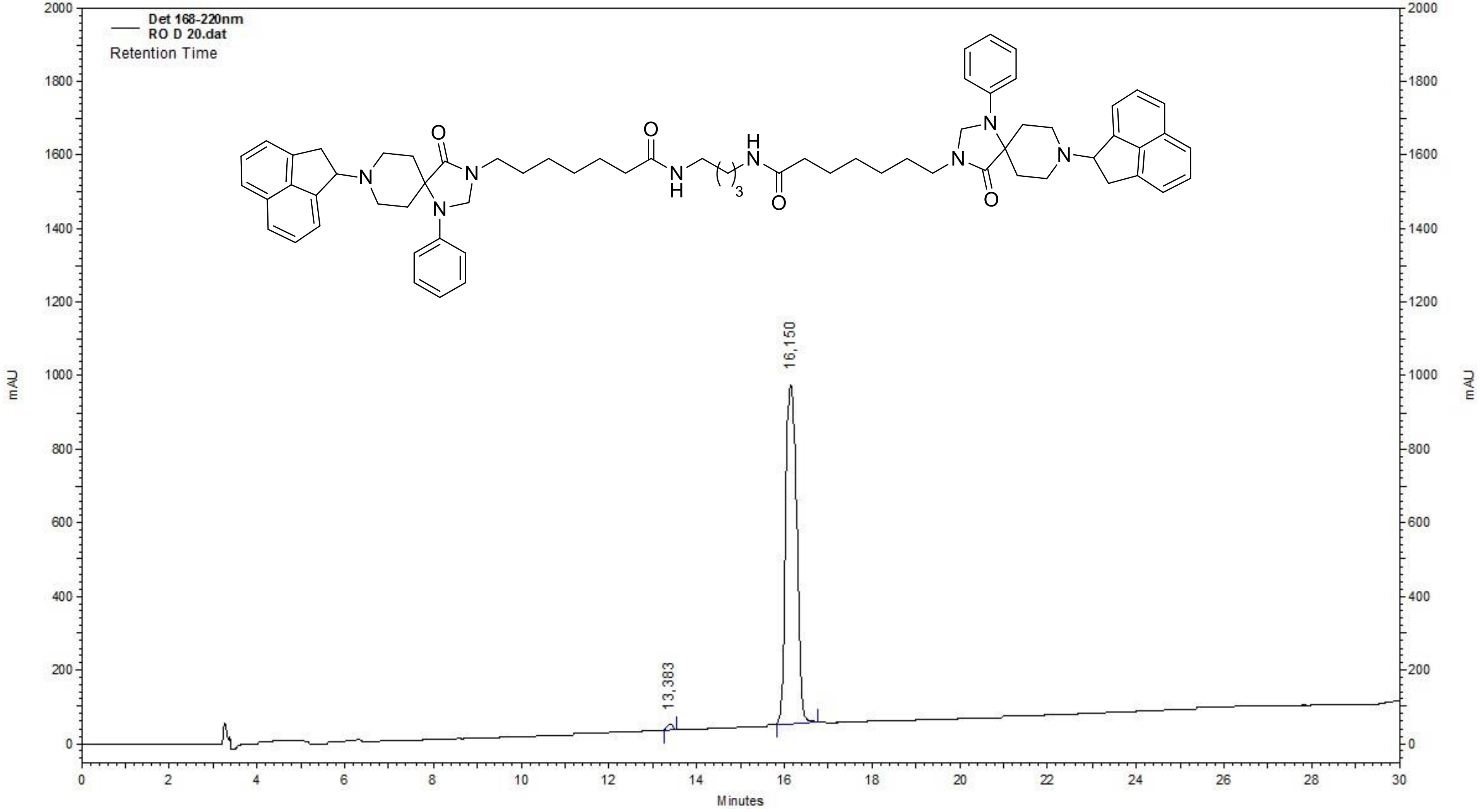

Det 168-220nm
RO D 20.dat
Retention Time
16,150
13,383
mAU
Minutes

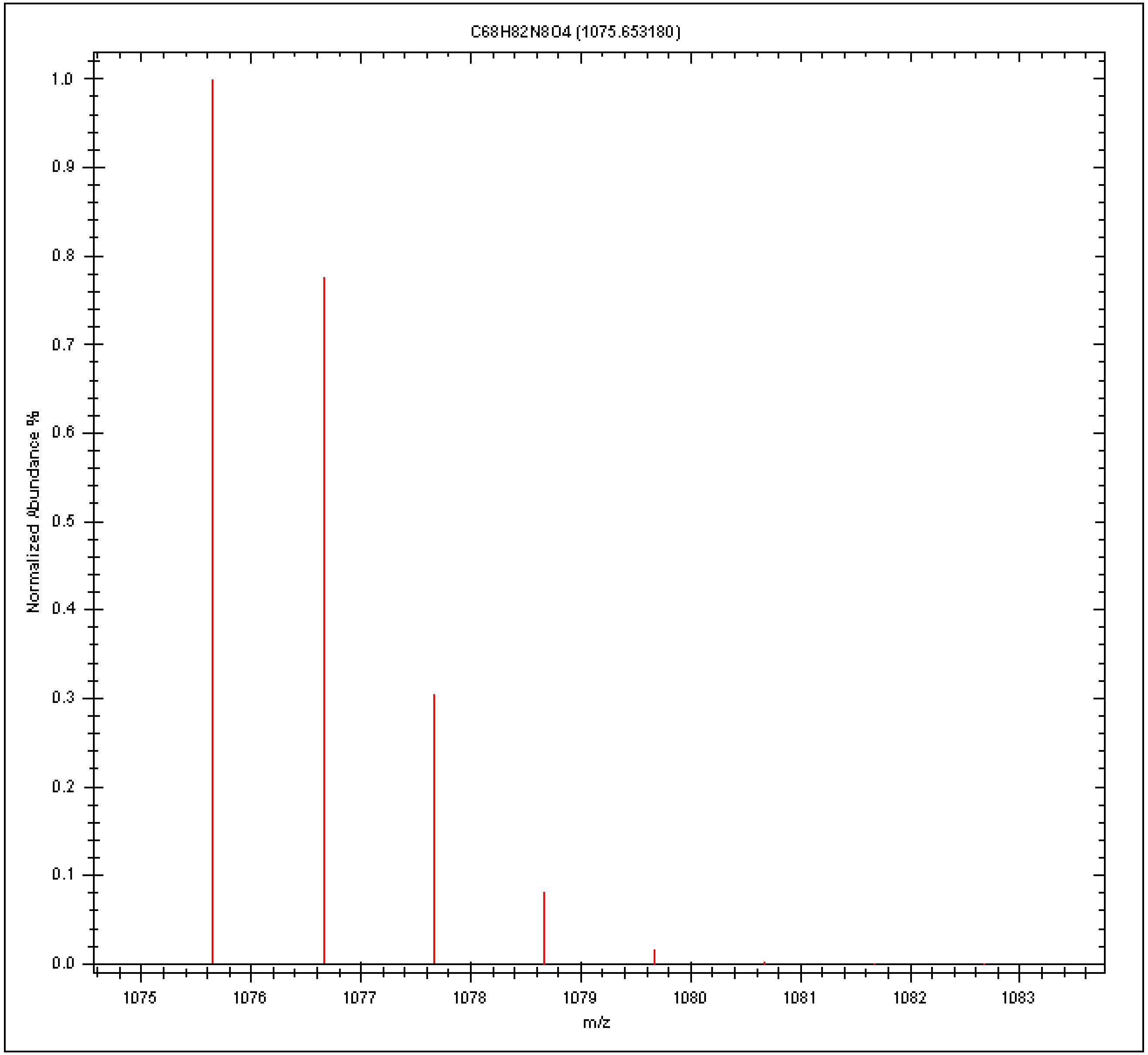

C68H82N8O4 (1075.653180)
Normalized Abundance %
1.0
0.9
0.8
0.7
0.6
0.5
0.4
0.3
0.2
0.1
0.0
1075
1076
1077
1078
1079
1080
1081
1082
1083
m/z

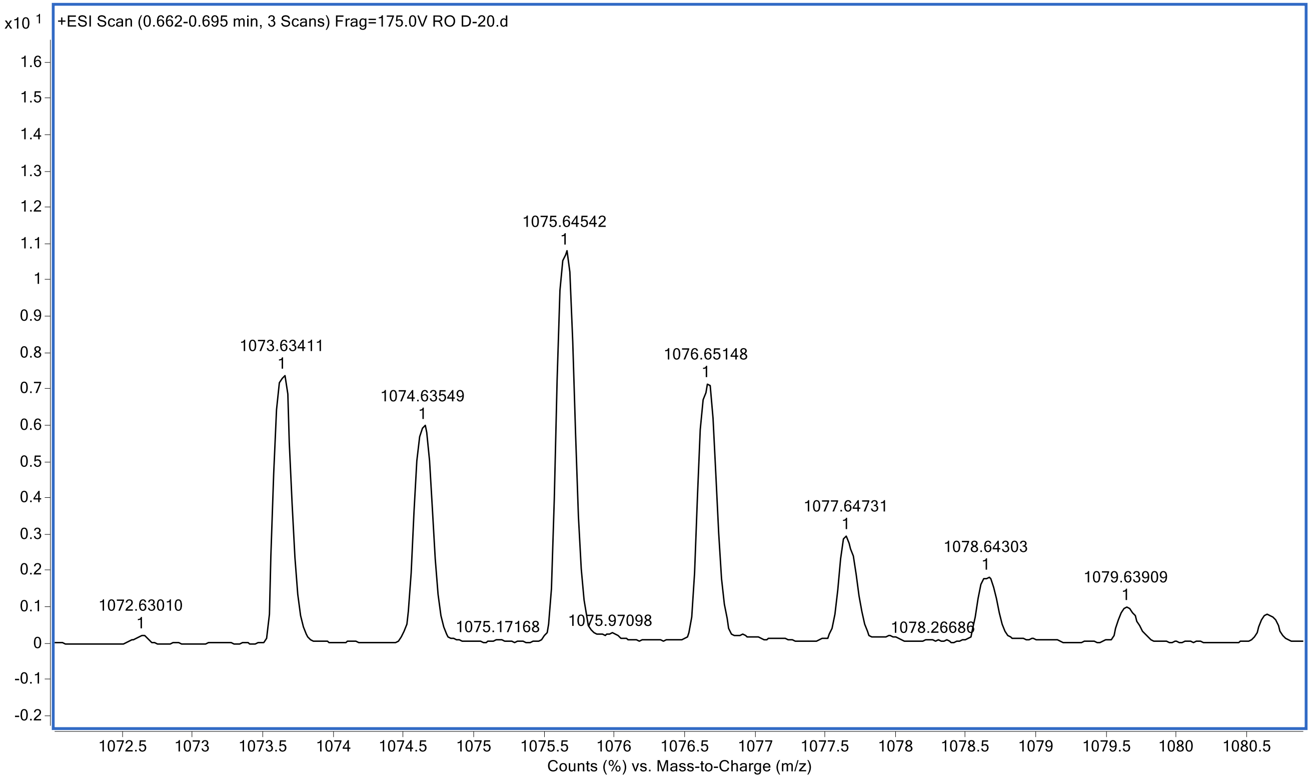
x10 1
+ESI Scan (0.662-0.695 min, 3 Scans) Frag=175.0V RO D-20.d
1072.63010
1073.63411
1074.63549
1075.17168
1075.64542
1075.97098
1076.65148
1077.64731
1078.26686
1078.64303
1079.63909
Counts (%) vs. Mass-to-Charge (m/z)

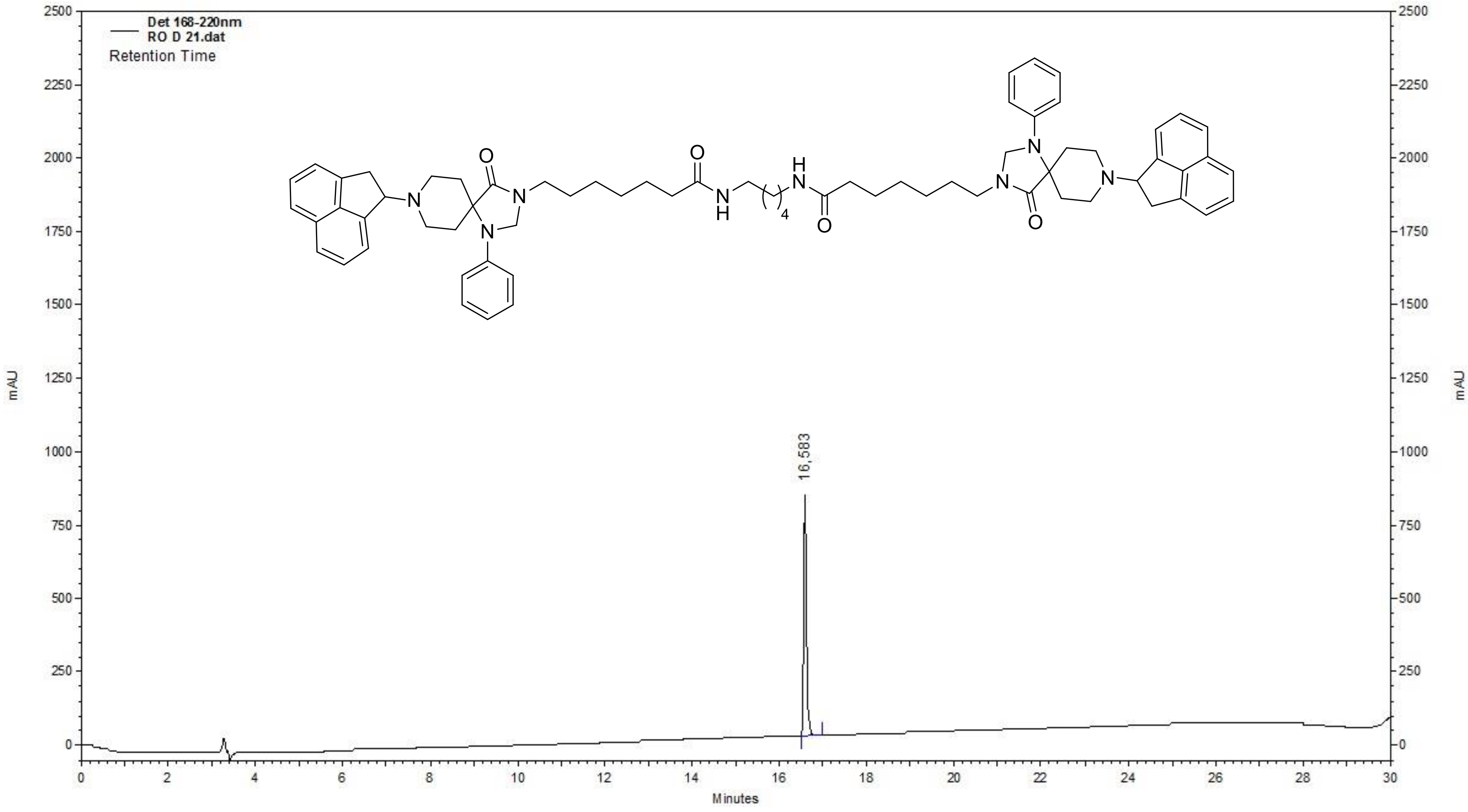
Det 168-220nm
RO D 21.dat
Retention Time
16,583
mAU
Minutes
O
N
H
4

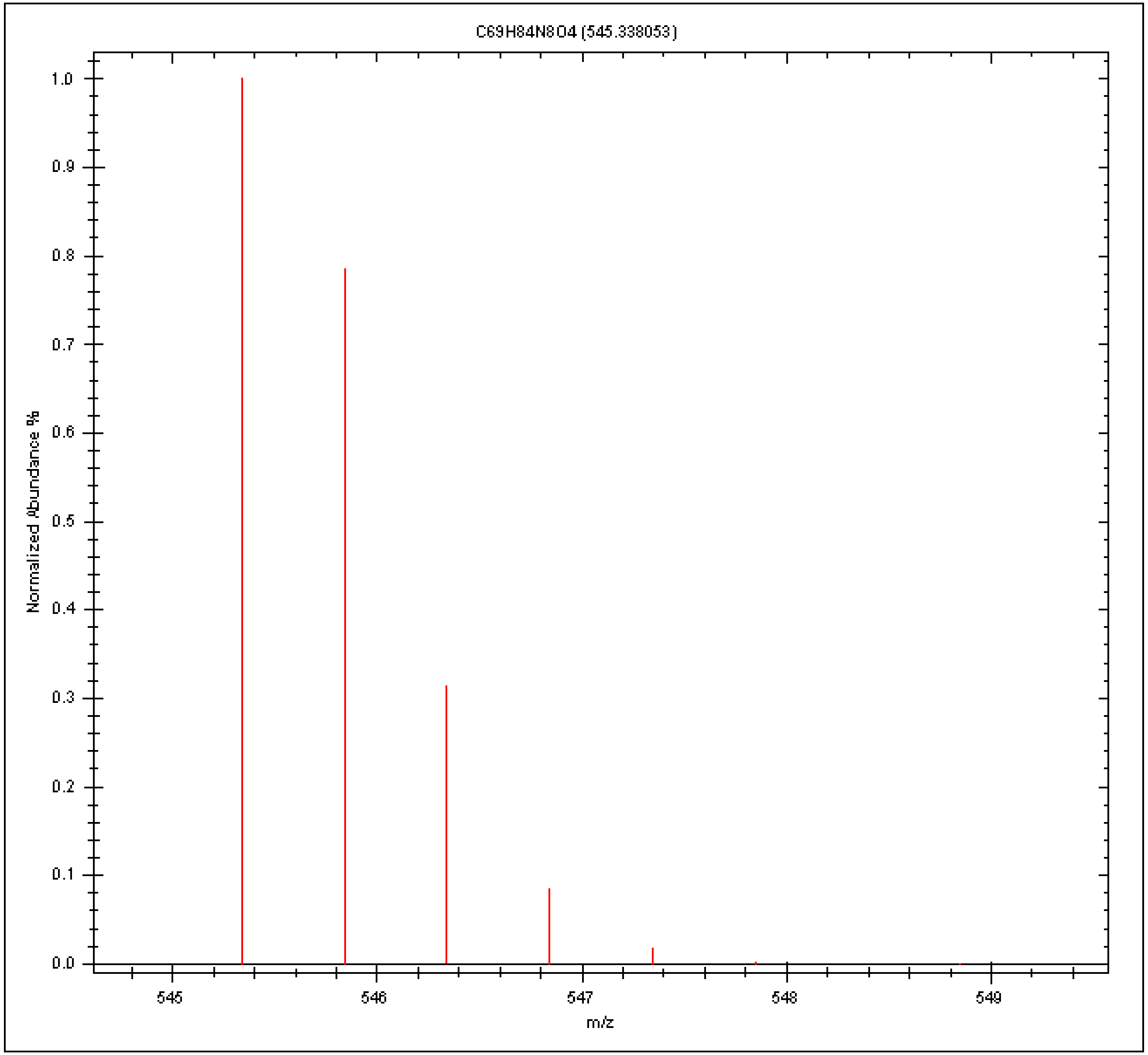

C69H84N8O4 (545.338053)
1.0
0.9
0.8
0.7
0.6
0.5
0.4
0.3
0.2
0.1
0.0
Normalized Abundance %
545
546
547
548
549
m/z

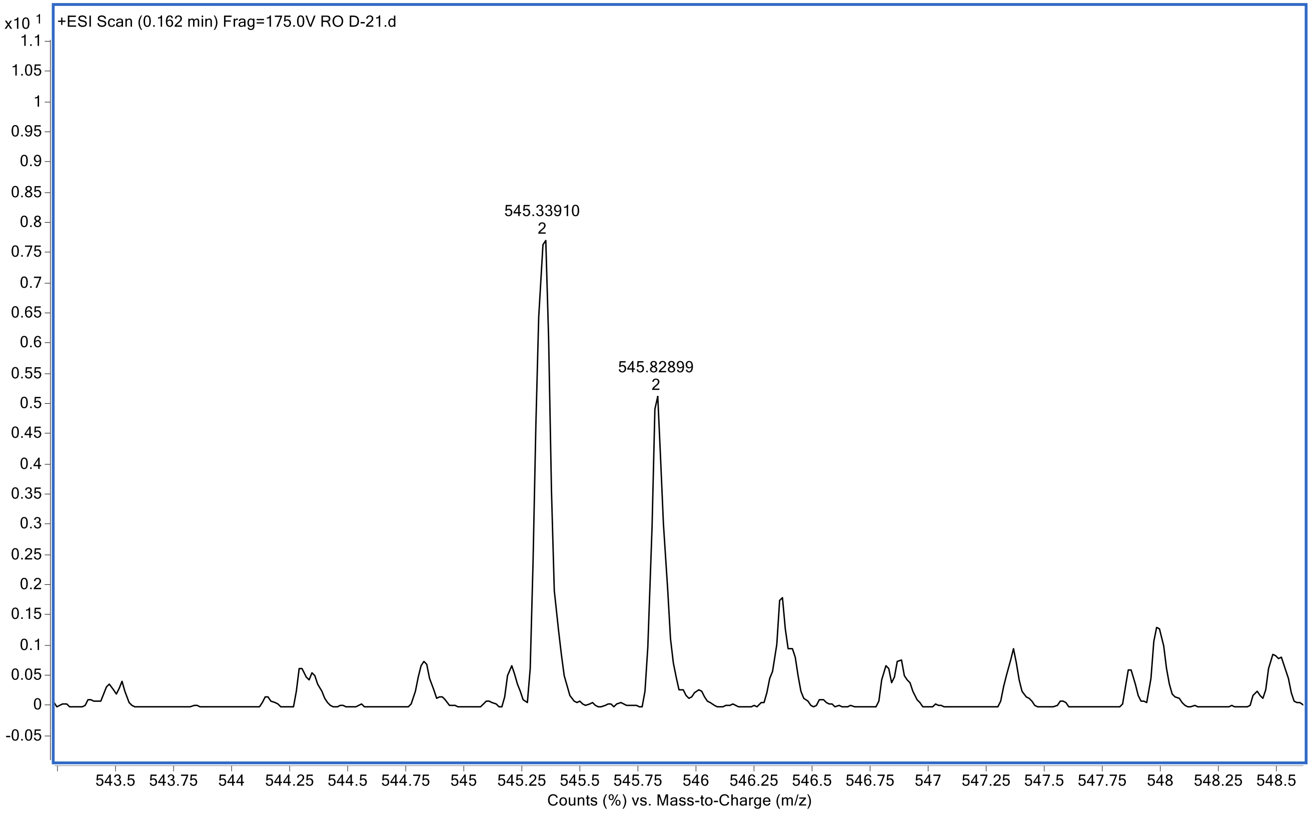
x10 1
+ESI Scan (0.162 min) Frag=175.0V RO D-21.d
545.33910
2
545.82899
2
Counts (%) vs. Mass-to-Charge (m/z)

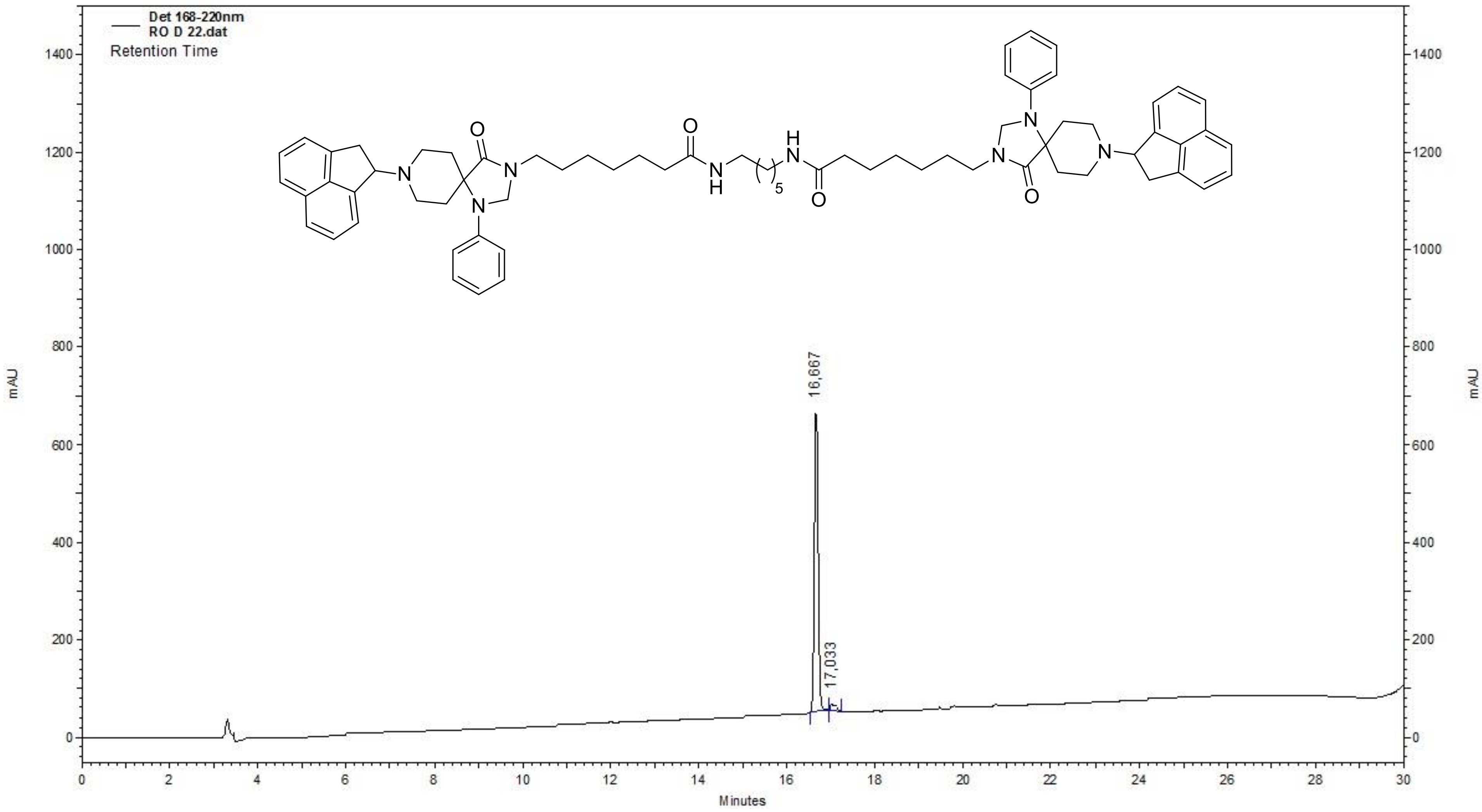

Det 168-220nm
RO D 22.dat
Retention Time
1400
1200
1000
800
600
400
200
0
mAU
16,667
17,033
0
2
4
6
8
10
12
14
16
18
20
22
24
26
28
30
Minutes

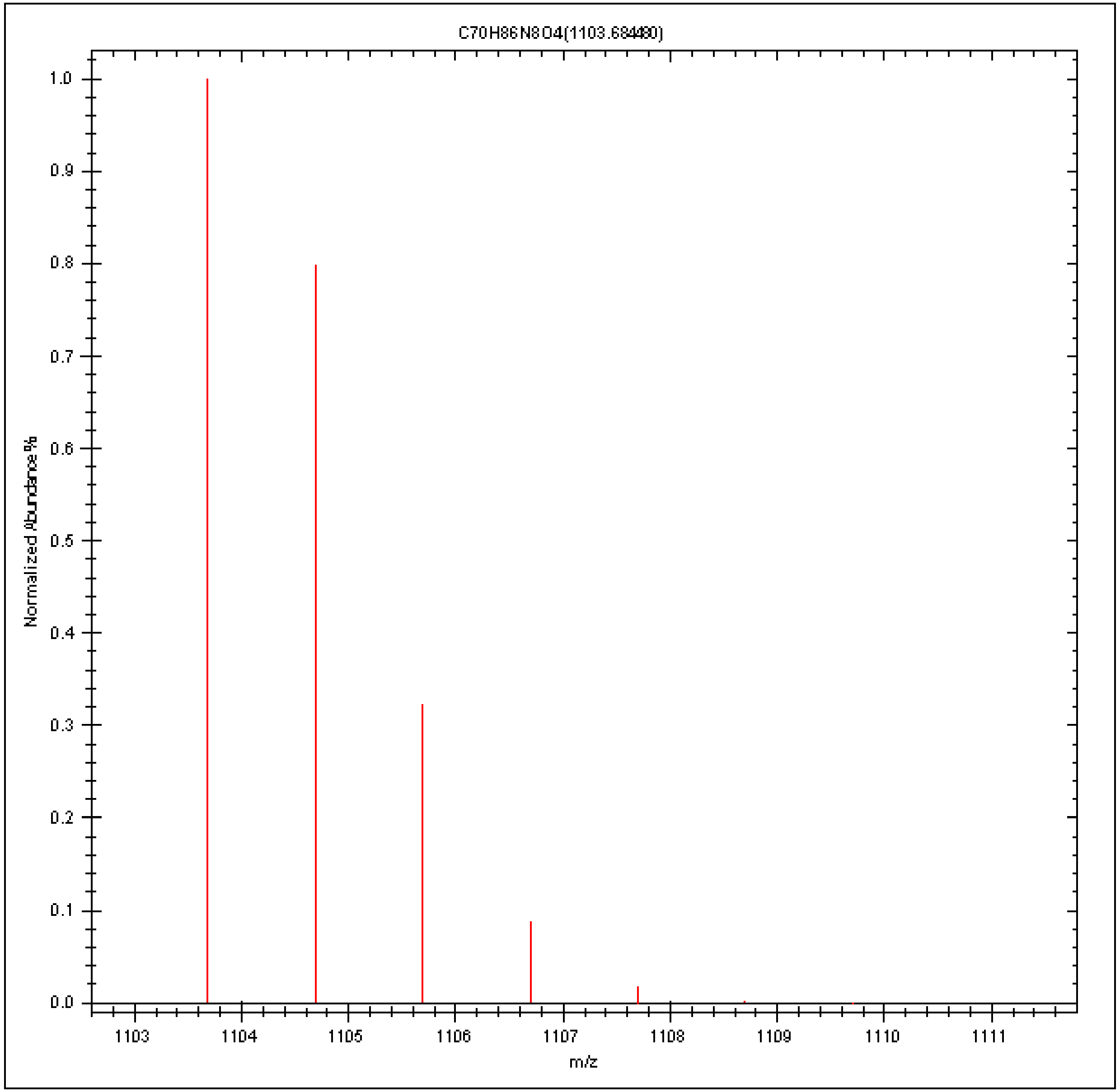
C70H86N8O4(1103.68480)
Normalized Abundance %
1.0
0.9
0.8
0.7
0.6
0.5
0.4
0.3
0.2
0.1
0.0
1103
1104
1105
1106
1107
1108
1109
1110
1111
m/z

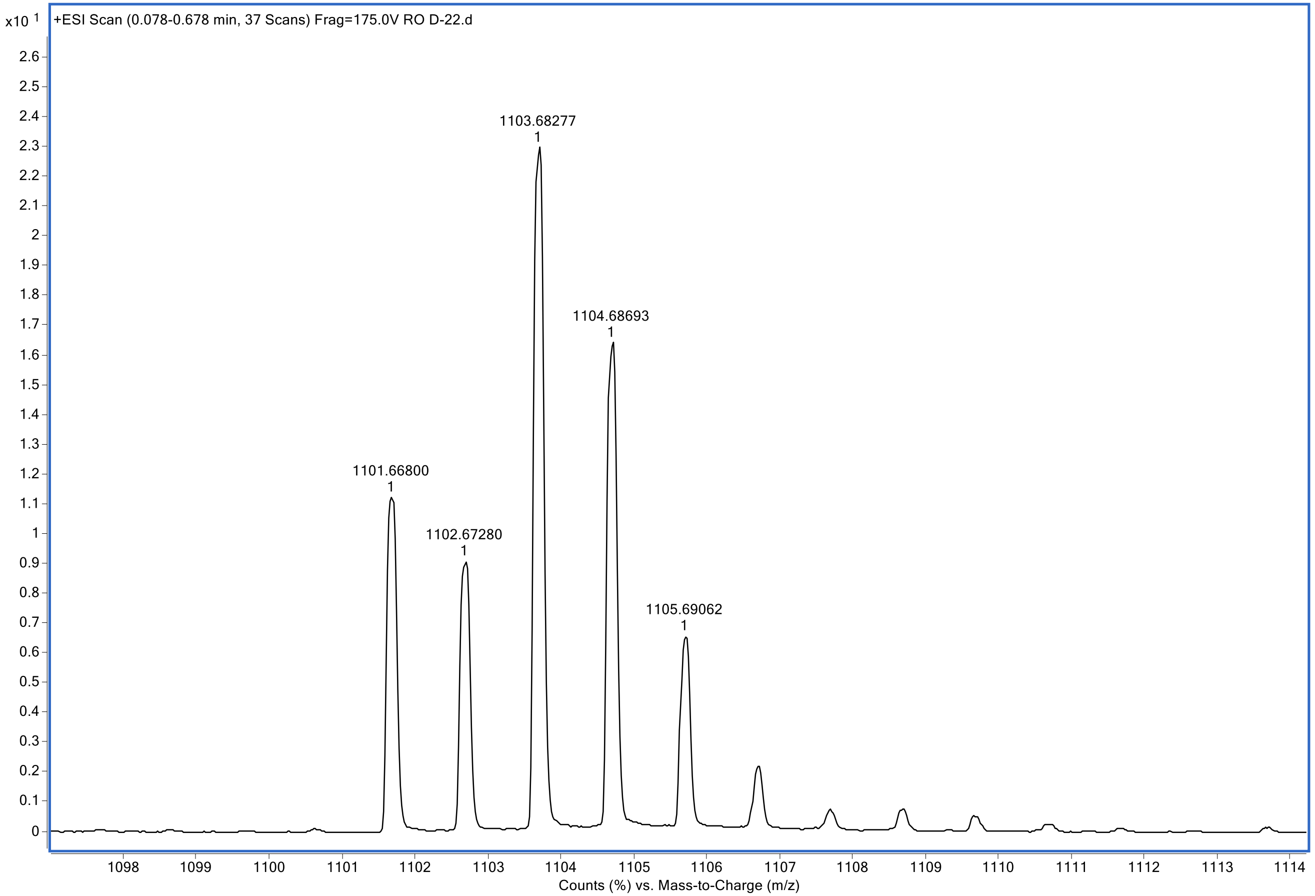
x10 1
+ESI Scan (0.078-0.678 min, 37 Scans) Frag=175.0V RO D-22.d
1103.68277
1
1104.68693
1
1101.66800
1
1102.67280
1
1105.69062
1
Counts (%) vs. Mass-to-Charge (m/z)

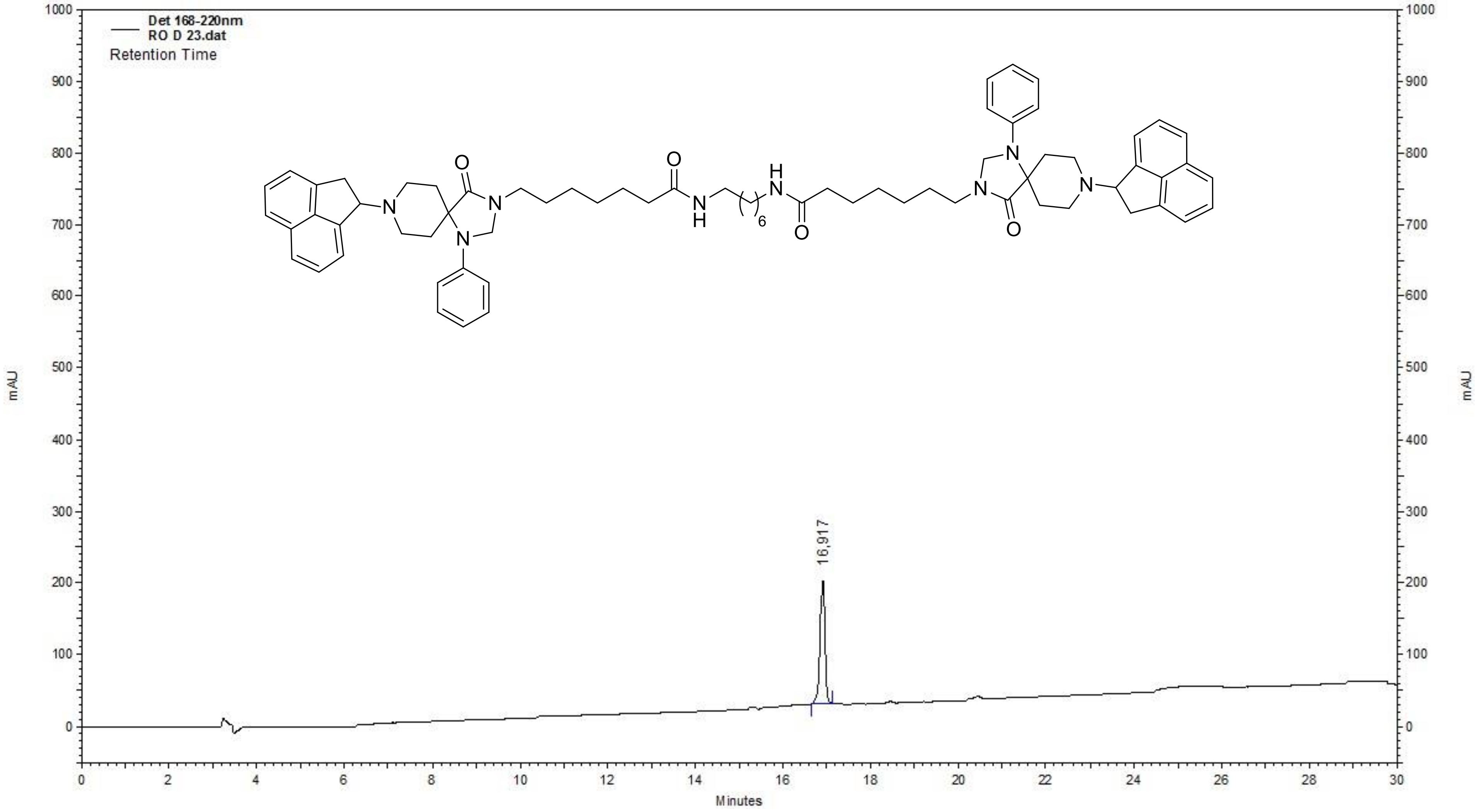

Det 168-220nm
RO D 23.dat
Retention Time
16,917
mAU
Minutes

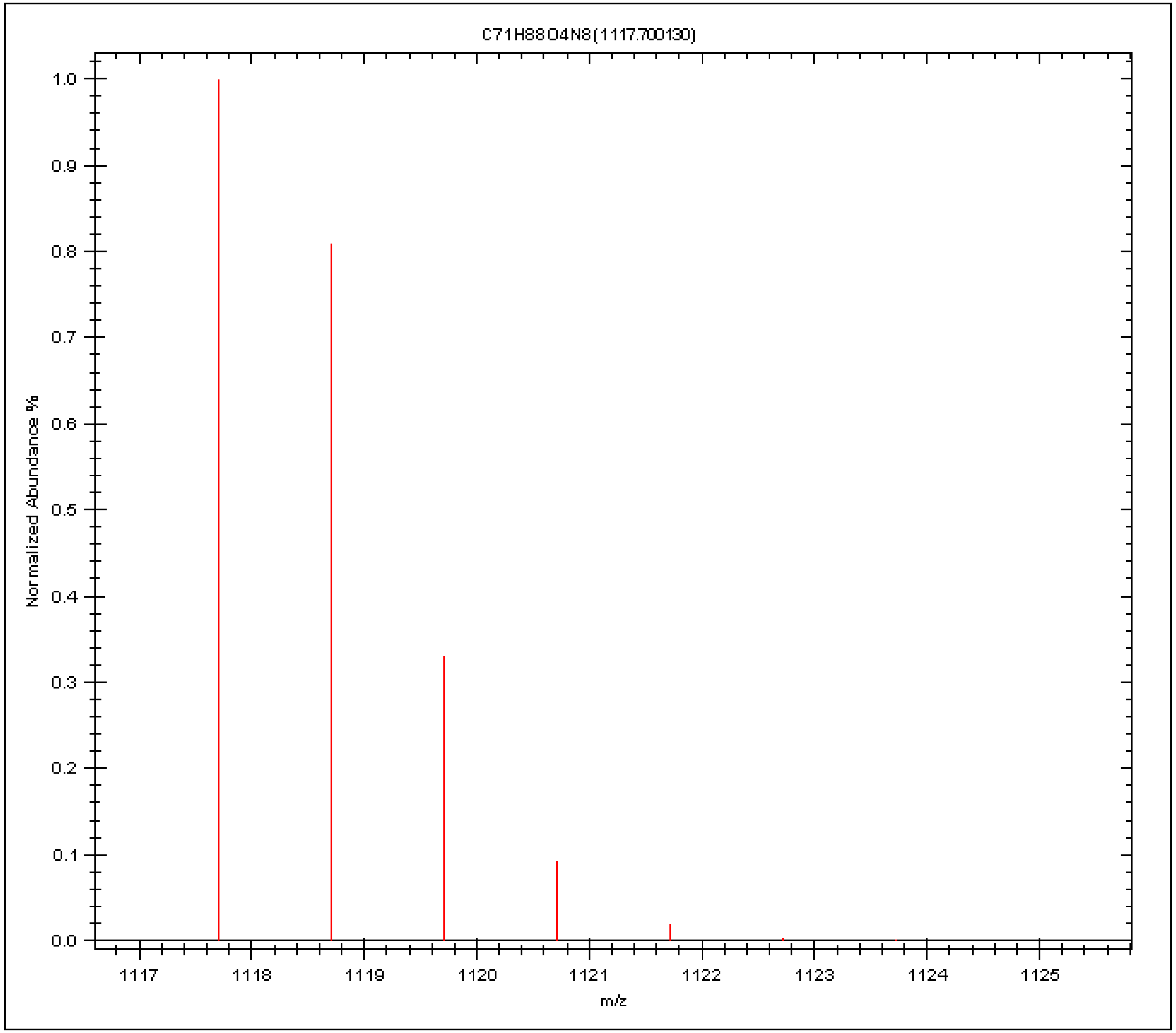

C71H88O4N8(1117.700130)
Normalized Abundance %
1.0
0.9
0.8
0.7
0.6
0.5
0.4
0.3
0.2
0.1
0.0
1117
1118
1119
1120
1121
1122
1123
1124
1125
m/z

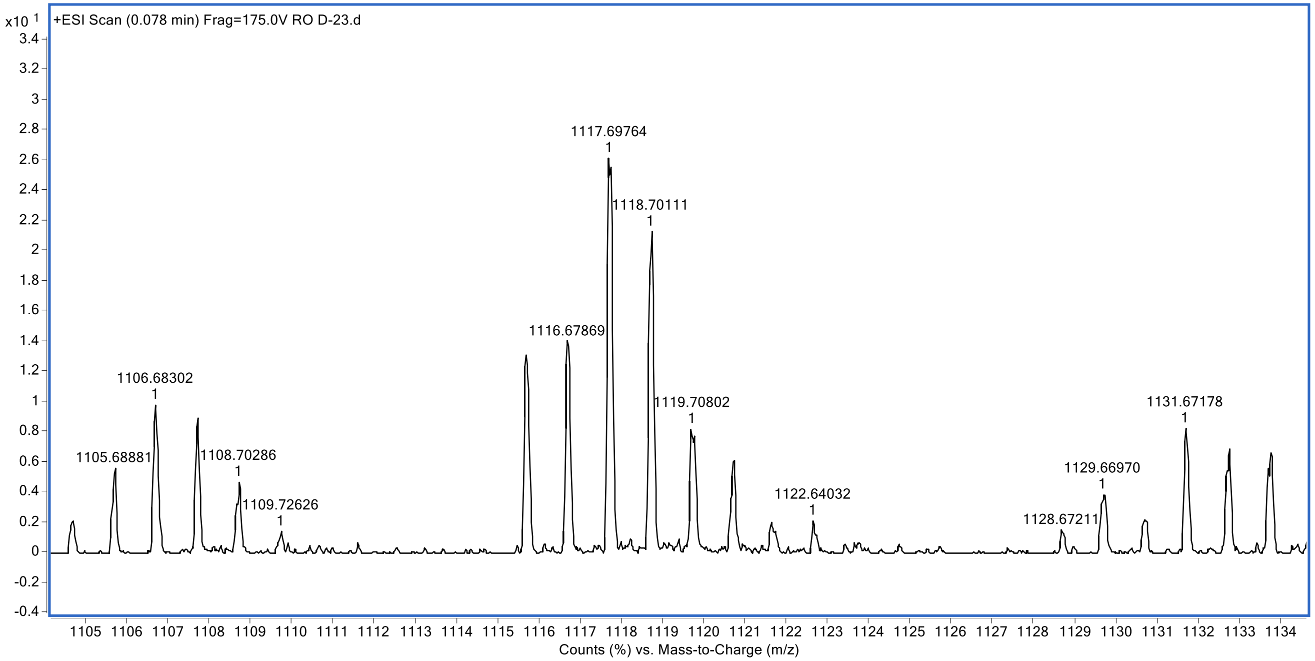

x10 1
+ESI Scan (0.078 min) Frag=175.0V RO D-23.d
1105.68881
1106.68302
1108.70286
1109.72626
1116.67869
1117.69764
1118.70111
1119.70802
1122.64032
1128.67211
1129.66970
1131.67178
Counts (%) vs. Mass-to-Charge (m/z)

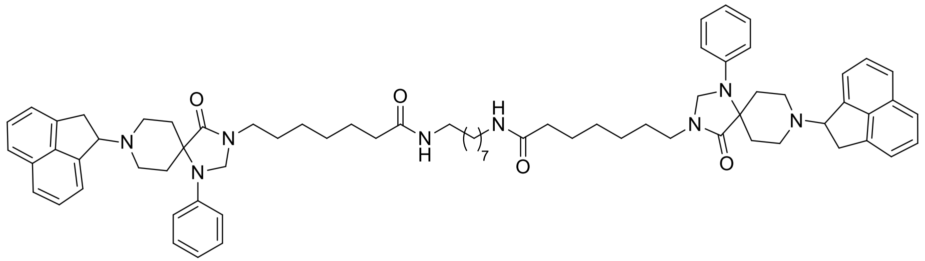

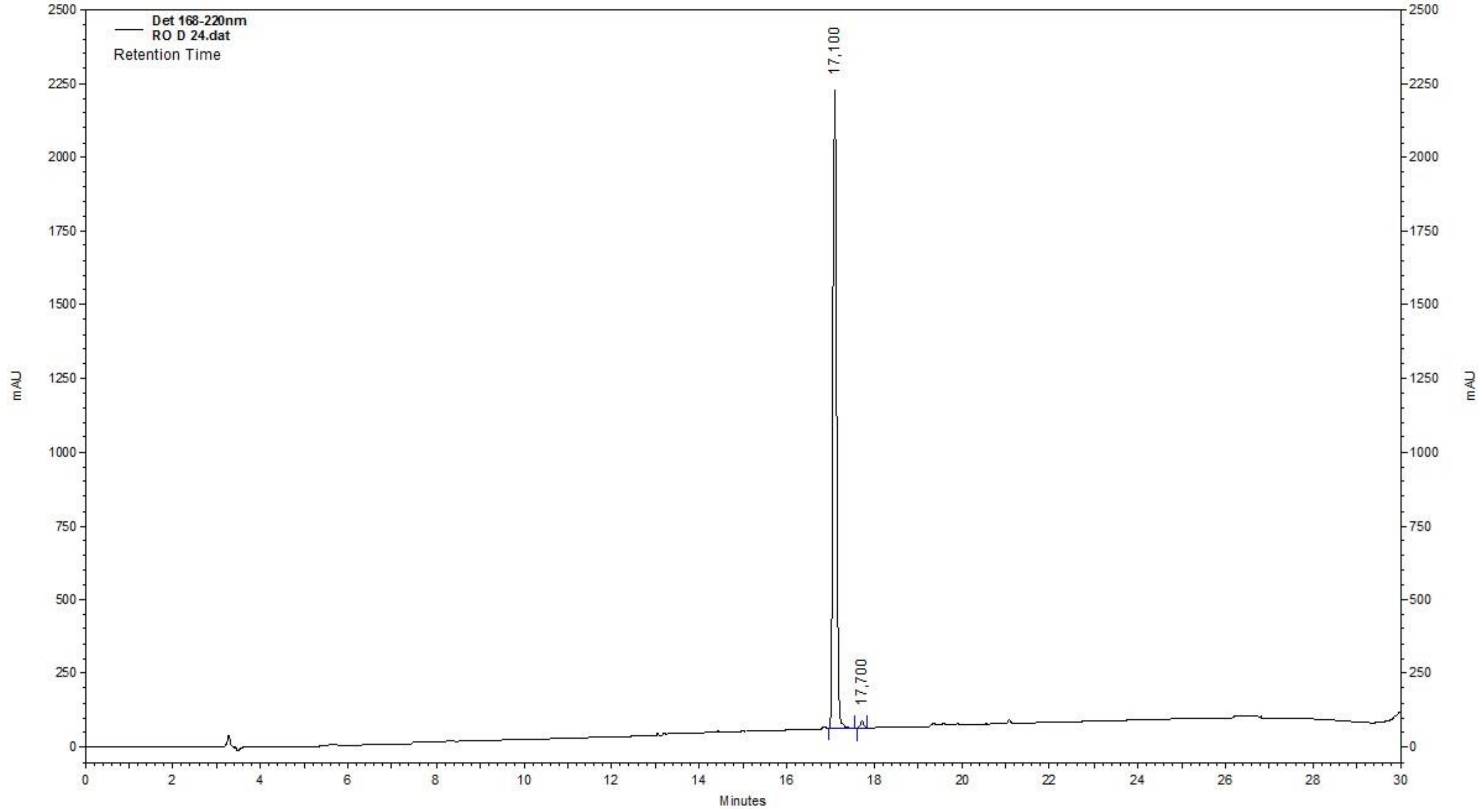

Det 168-220nm
RO D 24.dat
Retention Time
17,100
17,700
mAU
Minutes

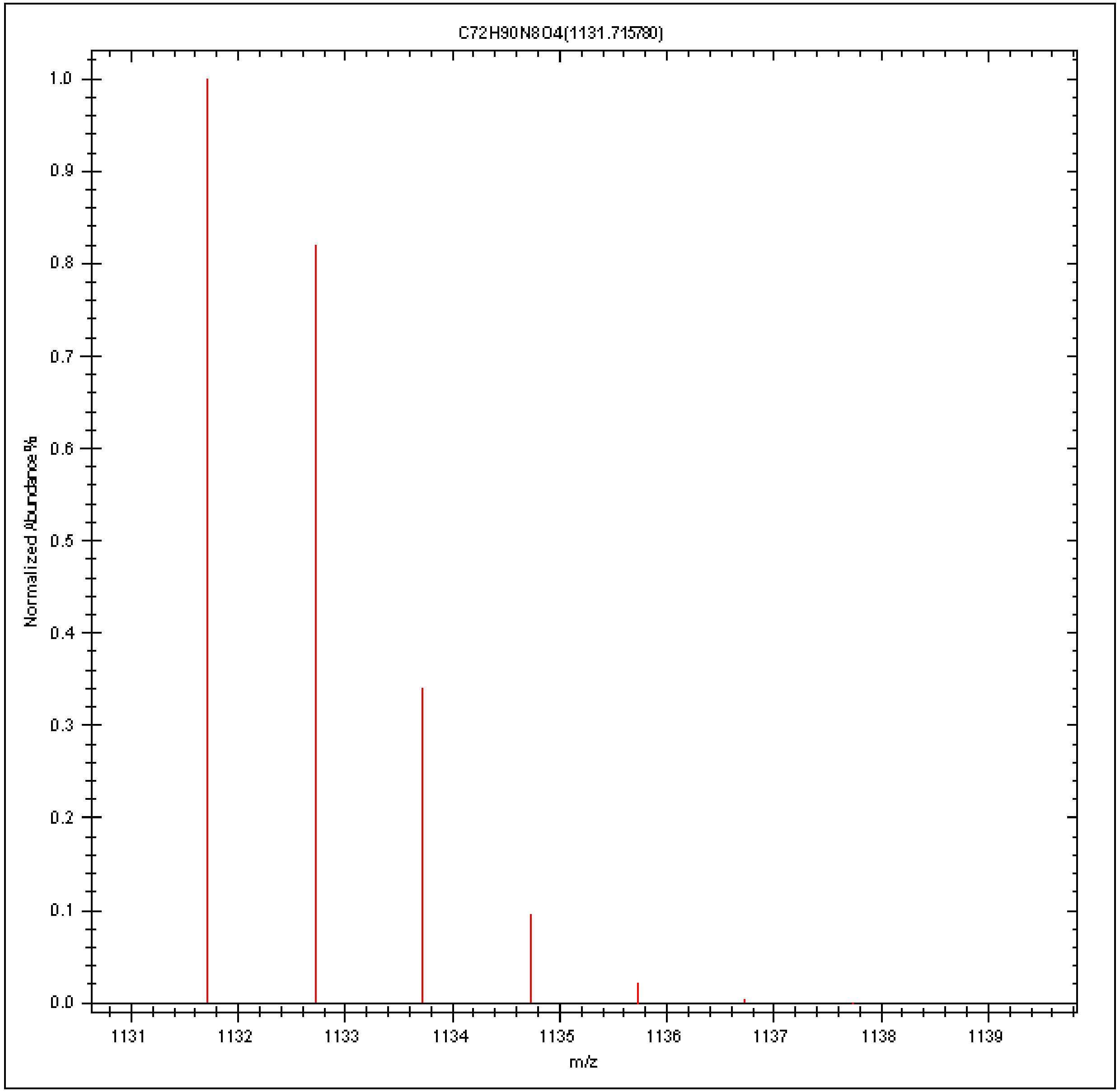
C72H90N8O4(1131.715780)
Normalized Abundance%
1.0
0.9
0.8
0.7
0.6
0.5
0.4
0.3
0.2
0.1
0.0
1131
1132
1133
1134
1135
1136
1137
1138
1139
m/z

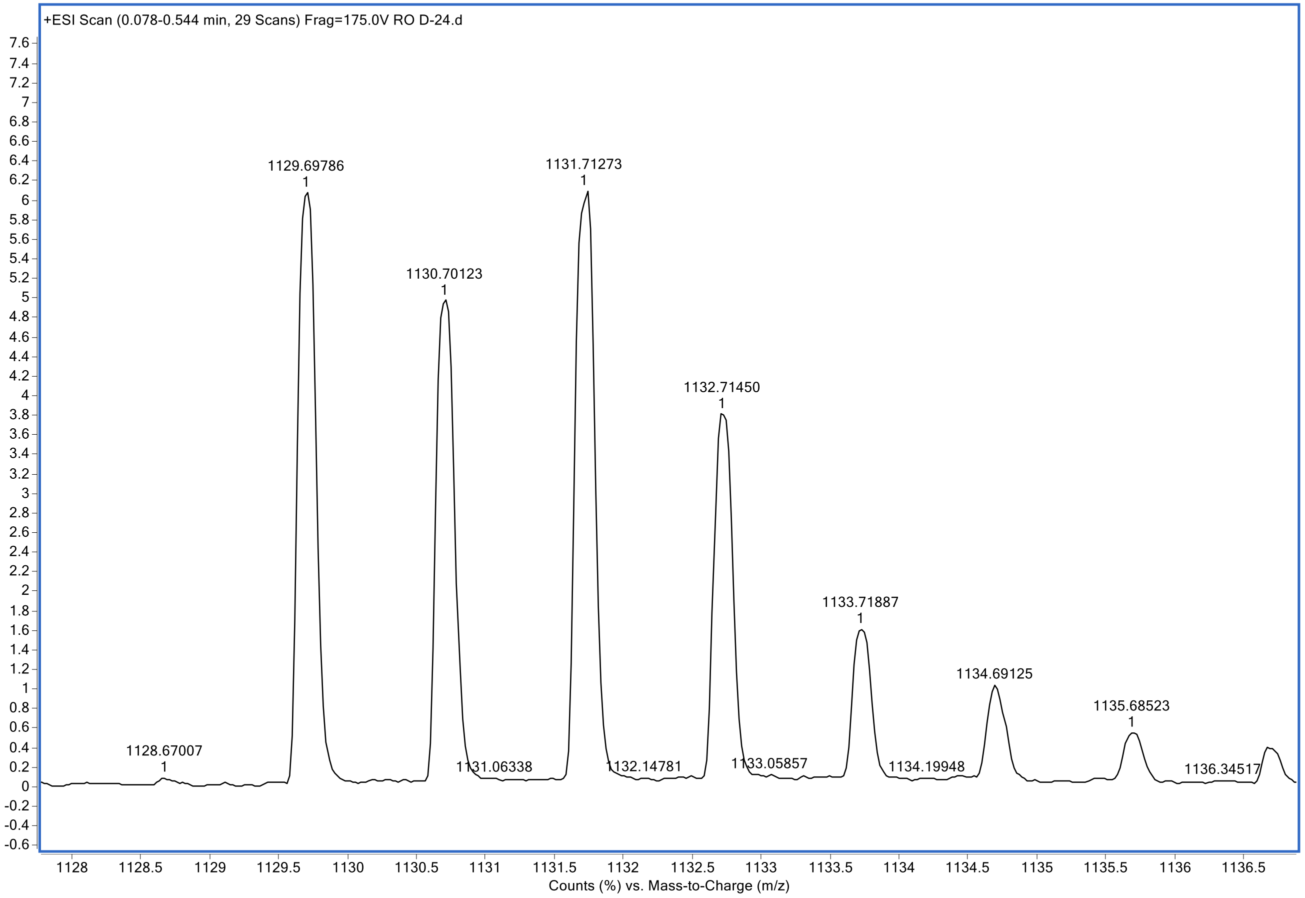

+ESI Scan (0.078-0.544 min, 29 Scans) Frag=175.0V RO D-24.d
1129.69786
1
1130.70123
1
1131.71273
1
1132.71450
1
1133.71887
1
1134.69125
1135.68523
1
1128.67007
1
1131.06338
1132.14781
1133.05857
1134.19948
1136.34517
Counts (%) vs. Mass-to-Charge (m/z)

Table S4. NMR Resonance Assignments[a] of Peptide **11**, in SDS-d_{25} 200 mM Solution.

Residue	NH ($^3J_{HN\text{-}H\alpha}$; $\Delta\delta/\Delta T$)[a]	$C^{\alpha}H$	$C^{\beta}H$	Others
Phe[1]		4.34	3.14, 3.27	7.30(δ); 7.20(ε); 6.94(ζ)
Gly[2]	8.34 (6,2; -4)	3.75		
Gly[3]	6.90 (7,9; 6,5)	3.61, 3.86		
Phe[4]	7.97 (7,3; -4,1)	4.73	3.05, 3.36	7.38(δ); 7.26(ε); 7.12(ζ)
Thr[5]	8.30 (6,98; -7.5)	4.33	4.33	1.20(γ)
Gly[6]	8.19 (6,6; -6,2)	3.89, 4.10		
Ala[7]	7.94 (4.8; -3,4)	4.16	1.39	
Arg[8]	8.19 (4,6; -5,6)	4.21	1.87	1.67(γ); 3.15(δ); 7.15(ε); 6.50, 6.85(η)
Lys[9]	7.88 (4,8; -2,2)	4.11	1.83	1.43(γ); 1.68(δ); 2.98(ε); 7.42(ζ)
Ser[10]	7.98 (4,8; -3,8)	4.34	3.90	
Ala[11]	7.91 (6,2; -0.2)	4.30	1.40	
Arg[12]	7.83 (6,8; -2,0)	4.22	1.81	1.66(γ); 3.16(δ); 7.14(ε)

[a] Temperature coefficients are in ppb/K. $^3J_{HN\text{-}H\alpha}$ are in Hz. Linker signals: HN 7.84 ppm; CH_2 α 3.50 ppm; CH_2 β 2.82 ppm

Table S5. NOE derived upper limit constraints for peptide **11**.

						Upper limit	Violation
1	PHE	HA	1	PHE	QD	6.01	
1	PHE	HA	2	GLY	HN	2.80	
1	PHE	HB2	2	GLY	HN	3.73	
1	PHE	HB3	2	GLY	HN	3.73	
1	PHE	QD	2	GLY	HN	6.72	
1	PHE	QD	2	GLY	HA1	7.62	
1	PHE	QD	2	GLY	HA2	7.62	
1	PHE	QD	3	GLY	HN	7.62	
1	PHE	QD	3	GLY	HA1	7.62	
1	PHE	QE	2	GLY	HN	7.62	
1	PHE	QE	3	GLY	HN	7.62	
1	PHE	QE	3	GLY	HA1	7.62	
1	PHE	QE	3	GLY	HA2	7.62	
1	PHE	HZ	4	PHE	QD	7.62	
1	PHE	HZ	4	PHE	QE	7.62	
1	PHE	HZ	4	PHE	HZ	5.50	
2	GLY	HN	3	GLY	HN	3.42	
2	GLY	HN	4	PHE	HN	4.60	
2	GLY	QA	4	PHE	HN	3.85	
3	GLY	HN	4	PHE	HN	3.30	
3	GLY	HA1	4	PHE	HN	3.08	
3	GLY	HA1	4	PHE	QD	7.62	
3	GLY	HA2	4	PHE	QD	7.62	
4	PHE	HN	4	PHE	HB2	2.93	
4	PHE	HN	4	PHE	HB3	3.14	
4	PHE	HN	4	PHE	QD	6.57	
4	PHE	HN	4	PHE	QE	7.62	
4	PHE	HN	5	THR	HN	3.52	
4	PHE	HN	5	THR	QG2	6.22	

4	PHE	HA	4	PHE	QD	7.62	
4	PHE	HB2	5	THR	HN	3.70	
4	PHE	HB2	6	GLY	HN	4.23	
4	PHE	HB3	5	THR	HN	3.70	
4	PHE	HB3	6	GLY	HN	4.20	
4	PHE	QD	5	THR	HN	7.28	
4	PHE	QD	5	THR	QG2	8.65	
4	PHE	QD	6	GLY	HN	7.62	
5	THR	HN	5	THR	QG2	4.51	
5	THR	HN	6	GLY	HN	3.33	
5	THR	HN	6	GLY	HA1	4.17	
5	THR	HN	6	GLY	HA2	4.17	
5	THR	HN	6	GLY	QA	3.64	
5	THR	HN	7	ALA	HN	3.86	
5	THR	HA	5	THR	QG2	3.43	
5	THR	HA	6	GLY	HN	3.24	0.34
5	THR	QG2	6	GLY	HN	5.32	
5	THR	QG2	7	ALA	HN	6.43	
6	GLY	HN	7	ALA	HN	2.86	
6	GLY	HN	7	ALA	QB	4.45	
6	GLY	QA	7	ALA	HN	3.23	0.23
6	GLY	QA	9	LYS	QB	3.66	
6	GLY	QA	9	LYS	QD	6.16	
7	ALA	HN	7	ALA	QB	3.77	
7	ALA	HA	8	ARG	HN	3.40	0.24
7	ALA	HA	10	SER	QB	3.56	
7	ALA	QB	8	ARG	HN	4.30	
8	ARG	HN	8	ARG	HB2	3.67	
8	ARG	HN	8	ARG	HB3	3.67	
8	ARG	HN	8	ARG	QB	3.21	
8	ARG	HN	8	ARG	HG2	4.91	
8	ARG	HN	8	ARG	HG3	4.91	

8	ARG	HN	8	ARG	QG	4.07	
8	ARG	HN	8	ARG	QD	5.94	
8	ARG	HN	9	LYS	HN	3.33	
8	ARG	HA	8	ARG	QB	2.68	
8	ARG	HA	8	ARG	HG2	3.86	
8	ARG	HA	8	ARG	HG3	3.86	
8	ARG	HA	8	ARG	QG	3.31	
8	ARG	HA	8	ARG	QD	5.42	
8	ARG	HA	8	ARG	HE	5.34	
8	ARG	HA	9	LYS	HN	3.21	
8	ARG	HA	10	SER	HN	3.73	
8	ARG	HA	11	ALA	HN	4.72	
8	ARG	HA	11	ALA	QB	4.82	
8	ARG	QG	8	ARG	HE	3.33	
8	ARG	QG	8	ARG	QH2	4.61	
9	LYS	HN	9	LYS	HB2	3.33	
9	LYS	HN	9	LYS	HB3	3.33	
9	LYS	HN	9	LYS	QB	2.81	
9	LYS	HN	9	LYS	QD	5.85	
9	LYS	HN	10	SER	HN	3.08	
9	LYS	HA	9	LYS	QB	2.64	
9	LYS	HA	9	LYS	QD	5.45	
9	LYS	HA	9	LYS	QE	6.38	
9	LYS	HA	9	LYS	QZ	6.31	
9	LYS	HA	10	SER	HN	3.50	0.37
9	LYS	HA	12	ARG	HN	3.95	
9	LYS	HB2	9	LYS	QE	6.38	
9	LYS	HB2	10	SER	HN	3.79	
9	LYS	HB3	9	LYS	QE	6.38	
9	LYS	HB3	10	SER	HN	3.79	
9	LYS	QB	10	SER	HN	3.09	
9	LYS	QG	9	LYS	QZ	7.19	

9	LYS	QD	10	SER	HN	6.38	
10	SER	HN	10	SER	HB2	3.14	
10	SER	HN	10	SER	HB3	3.14	
10	SER	HN	11	ALA	QB	4.88	
10	SER	HA	10	SER	HB2	2.90	
10	SER	HA	10	SER	HB3	2.90	
10	SER	HA	11	ALA	HN	2.99	0.66
10	SER	QB	12	ARG	HN	4.15	
11	ALA	HN	11	ALA	QB	4.02	
11	ALA	HA	12	ARG	HN	3.08	
11	ALA	QB	12	ARG	HN	4.33	
12	ARG	HN	12	ARG	HA	2.83	
12	ARG	HN	12	ARG	HB2	3.14	
12	ARG	HN	12	ARG	HB3	3.14	
12	ARG	HN	12	ARG	QG	4.86	
12	ARG	HN	12	ARG	QD	6.07	
12	ARG	HN	12	ARG	NE	5.41	
12	ARG	HA	12	ARG	QD	5.23	
12	ARG	HA	12	ARG	NE	5.41	
12	ARG	QB	12	ARG	NE	4.05	

Table S6.Torsion angles of peptide **11** conformers.

Residue	φ^a	ψ	$\chi 1$
Family #1			
1	-	-171.6 ± 1.9	23.7± 6.1
2	-97.5± 17.5	-0.6± 6.5	
3	-91.8± 1.6	26.4± 0.5	
4	-116.7± 6.2	-64.7± 2.7	-118.6± 2.7
5	-175.4± 0.0	34.4± 0.1	17.4± 0.0
6	-112.1± 0.1	-15.6± 0.1	
7	-62.3± 0.2	-25.4± 0.2	39.5± 105.2
8	-81.6± 0.1	0.6± 1.0	-58.0± 5.1
9	-100.4± 1.3	-23.0± 0.2	-117.4± 0.0
10	-100.6± 0.3	-61.4± 0.6	-121.6± 0.1
11	-77.8± 1.6	3.1± 2.2	51.9± 87.7
12	-51.4± 55.2	-29.7± 95.4	-150.9± 1.8
Family #2			
1	-	-176.9± 10.7	-176.6± 60.3
2	149.0± 18.1	25.8± 37.4	
3	-103.3± 14.8	28.6± 3.6	
4	-110.6± 8.0	-65.7± 2.7	-118.8± 2.8
5	-175.4± 0.0	34.4± 0.1	17.4± 0.0
6	-112.1± 0.0	-15.6± 0.0	
7	-62.3± 0.1	-25.3± 0.2	74.4± 22.1
8	-81.5± 0.2	0.3± 0.2	-56.8± 4.6
9	-100.0± 0.2	-23.1± 0.0	-117.5± 0.0
10	-100.6± 0.2	-61.2± 0.3	-121.6± 0.1

11	-78.4± 0.4	3.7± 0.7	-79.3± 79.9
12	-24.6± 34.2	115.8± 17.5	-161.8± 24.4

[a]Angles value (degree) of the mean structure (± standard deviation) of the 5 lowest energy conformers of family #1 and 5 of family #2 of peptide **11**.

Figure S1. Secondary shifts of the α protons of peptide **11** in SDS solution.[a]

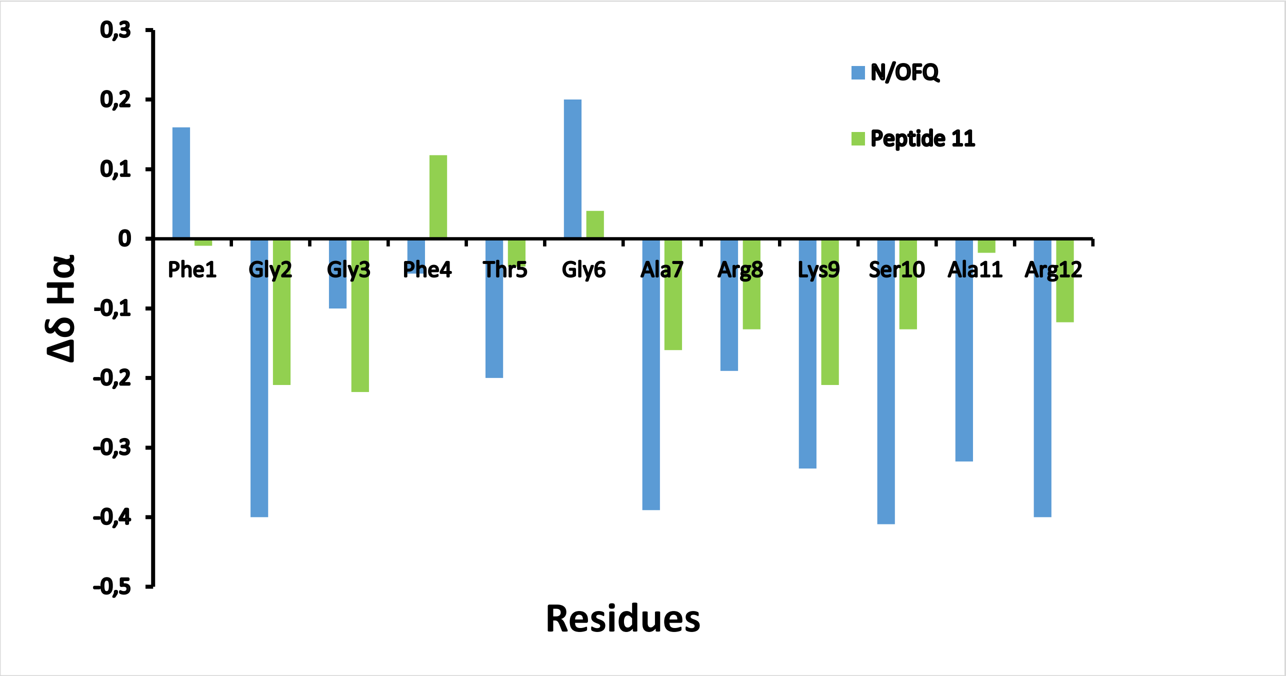

[a] N/OQF chemical shifts were taken from Gemma, A. et al. *Org. Biomol. Chem.* **2011**, *9*, 6133-6142. Reference Hα chemical shift values were taken from Andersen, N. H. et al. FEBS Lett. **1996**, *399*, 47-52.
